# Supplementary material for: Examining the physical and psychological effects of combining multimodal feedback with continuous control in prosthetic hands
Source: Sci Rep. 2025 Jan 29;15:3690. doi: 10.1038/s41598-025-87048-x (PMC11779825; doi:10.1038/s41598-025-87048-x)
Supplement: Supplementary file 1 — Supplementary Information 1. [file 41598_2025_87048_MOESM1_ESM.pdf]

Supplementary information

This PDF file includes:

Section S1 - Full Implementation Details of the Discrete and Continuous Controllers
Section S2 - Full Implementation Details of the Haptic Feedback Armband
Section S3 - Full Setup Details of the Clinical Dexterity Assessments
Figs. S12 to S22
Tables S2 and S4
Transcripts S1 to S10
References[72] to[75]

Other Supplementary Material for this manuscript includes the following:

Movie S1 - Controller Implementation Details
Movie S2 - Demonstration of Prosthetic Hand Control
Movie S3 - Session 1
Movie S4 - Session 2
Movie S5 - Session 3
Movie S6 - Summary Video

S1 Full Implementation Details of the Discrete and Continuous Controllers

The discrete and continuous controllers in this work share a common methodology. First, a series of 'reference' EMG data are recorded. Then, live EMG signals are compared to the reference data via a distance metric. In discrete control, the closest reference (as measured by the distance metric) is selected as the desired action. Meanwhile, in continuous control, a combination of references is formed to approximate the live EMG signal in such a way as to minimise the distance between the approximated data and the live data. The computed weightings of the reference combination correspond to the motion of controllable degrees of freedom of the prosthetic hand.

Preprocesing Muscle Activity

In this work, EMG signals are sampled at 8 points around the forearm of a user at a frequency of 200 Hz with a Myo armband. Since this is below the Nyquist frequency of EMG signals of 1000 Hz[72], temporal features of an incoming EMG signal $s(k)$ at control step k are discarded by taking the mean absolute value (MAV) $I(k)$ over $W = 10$ samples:

$$I(k) = \frac{1}{W}\sum_{w=1}^{W} |s(k+w-W)|. \tag{S1.1}$$

This gives a response time of approximately 50 ms, below the 200 ms maximum delay stipulated in literature[74].

Because temporal features are not kept, we focus on the spatial relationships between incoming EMG signals. Given that there are 8 electrodes equally spaced around the forearm, we consider each processed sample to be two-dimensional $x = [I, \theta]^T$, comprised of the intensity I computed in S1.1, at circumferential position around the forearm θ. Because circumferential position around the forearm wraps, samples belong to a cylindrical space, shown in Fig. S1.

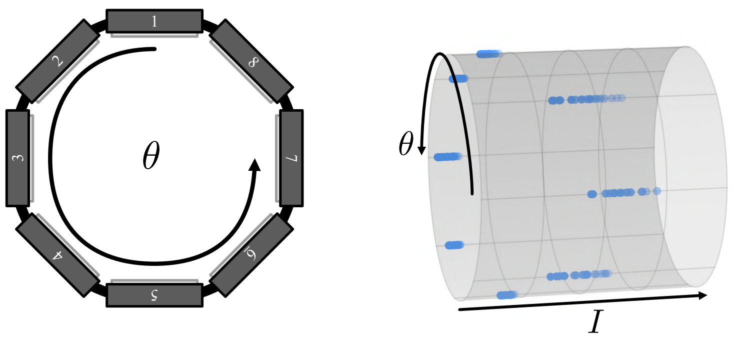

Figure S1. Cylindrical EMG Space. Left: the circumferential position θ of each of the 8 electrodes in the Myo armband. Right: Illustrative cylindrical space that EMG samples lie on, with the I axis corresponding to the intensity of MAV samples, and θ axis corresponding to the circumferential position of the sample around the forearm.

Kernel Density Estimation

As introduced in[15], Kernel Density Estimation (KDE) is used to estimate the underlying probability distribution that produces observed EMG signals. The intensity dimension, I, is limited by the minimum and maximum sensing limits of the electrodes, so a truncated Gaussian kernel is used:

$$p(I|\mu, \sigma, I_l, I_u) = \mathrm{TrG}(I|\mu, \sigma, I_l, I_u) \propto \frac{\exp\left(-\frac{(I-\mu)^2}{2\sigma^2}\right)}{\Phi(\frac{I_u-\mu}{\sigma}) - \Phi(\frac{I_l-\mu}{\sigma})}, \tag{S1.2}$$

where $\Phi(a) = \frac{1}{2}(1-\mathrm{erf}(-\frac{a}{\sqrt{2}}))$. An example of the truncated nature of this kernel is shown in Fig. S2, Left. The circumferential position dimension, θ, is wrapped, so a von Mises distribution, also known as a circular Gaussian, is used:

$$p(\theta|\nu, \kappa) = \mathrm{vM}(\theta|\nu, \kappa) \propto \exp\Big(\kappa \cos\left(2\pi(\theta-\nu)\right)\Big), \tag{S1.3}$$

where we have scaled the circumferential position $\theta - \nu$ such that a full rotation around the forearm is equal to 1. An example of the wrapped nature of this kernel is shown in Fig. S2, Middle.

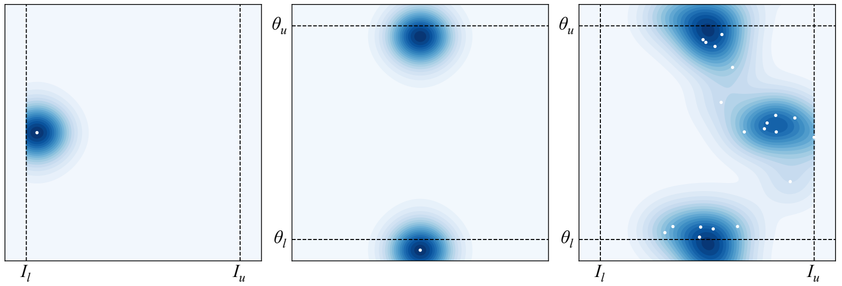

Figure S2. Kernel density estimation. Illustrative two-dimensional kernel density estimation plots, darker blue indicates a higher estimated probability, white points indicate observed data. The horizontal axis represents the intensity of observed samples, I, and the vertical axis represents the circumferential position of the sample around the forearm, θ. Left: example of the truncated Gaussian kernel used in the linear dimension. Middle: example of the von Mises kernel used in the wrapped dimension. Right: example generated KDE from multiple randomly sampled points.

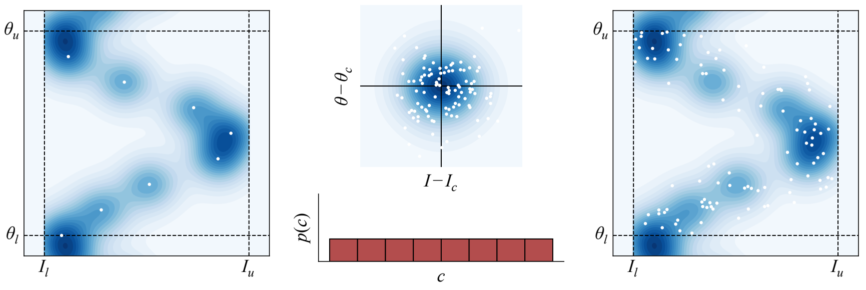

Figure S3. Sampling from a kernel density estimate. Illustrative example showing sampling from a kernel density estimate. Left: Original KDE and component data. Upper Middle: intensities sampled from the two dimensional kernel making up the KDE. Lower Middle: component assignment probabilities as a uniform distribution. Right: Data resampled from the KDE to obtain a more representative data sample.

The two dimensional kernel K_h formed from the outer product of the individual one dimensional kernels is used for kernel density estimation. After the estimate is produced (an example is shown in Fig. S2, Right), it is normalised to produce a valid probability distribution:

$$p(x) = \mathrm{KDE}(x, |\, \mathbf{x}_{c=1:C}) = \frac{1}{C}\sum_{c=1}^{C} K_h(x - x_c). \tag{S1.4}$$

Sampling from a KDE

A kernel density estimate forms a complex distribution that is typically difficult to sample from if evaluating the complete probability density function is required. A much more efficient way (illustrated in Fig. S3) to draw a sample $x_j = [I_j,\, \theta_j]^T$ from a KDE is to draw a sample $x_j^0 = [I_j^0, \theta_j^0]^T$ from the kernel distribution K_h (Fig. S3, upper middle), and randomly assign it to component $x_c = [I_c,\, \theta_c]^T$ with uniform probability (Fig. S3, lower middle):

$$x_j = x_j^0 + x_c, \quad x_j^0 \sim K_h, \quad c \in \{1, \ldots, C\}. \tag{S1.5}$$

Conditionally sampling from a multivariate KDE is more complex, because it requires the evaluation of the probability density function of the KDE at a given location in one or more dimensions. For the two dimensional KDE used in this work, sampling intensity I_j at a given circumferential location $\theta = \Theta$ can be achieved with a similar method to (S1.5), and is illustrated in Fig. S4. First, evaluating the conditional probability of each component at Θ according to the Kernel used:

$$p(\theta = \Theta)_{c=1:C} = [\mathrm{vM}(\theta = \Theta | \nu,\, \kappa)_1, \ldots, \mathrm{vM}(\theta = \Theta | \nu,\, \kappa)_C]^T. \tag{S1.6}$$

These probabilities are then normalised to form the basis of a multinomial distribution (Fig. S4, lower middle) from which to assign intensity samples I_j^0 drawn from the kernel distribution along the intensity axis (Fig. S4, upper middle):

$$I_j = I_j^0 + I_c, \quad I_j^0 \sim \mathrm{TrG}(I | \mu, \sigma, I_\mathrm{l}, I_\mathrm{u}), \quad c \sim \mathrm{Mult}(c |\, p(\theta = \Theta)_{c=1:C}). \tag{S1.7}$$

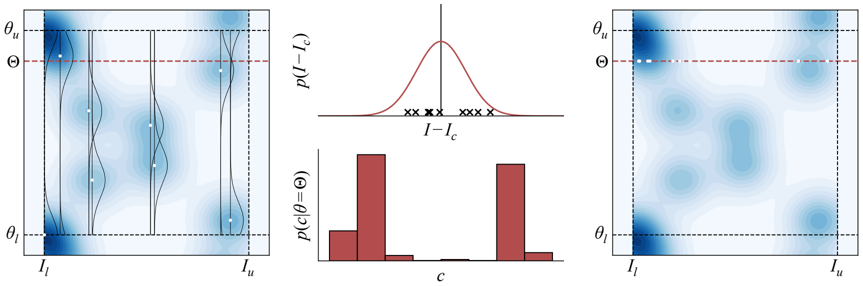

Figure S4. Conditionally sampling from a kernel density estimate. Illustrative example showing conditional sampling from a kernel density estimate at the point $\theta = \Theta$. Left: Original KDE and test components, with individual component kernel probabilities along the θ axis shown. Upper Middle: intensities sampled from the truncated Gaussian kernel. Lower Middle: component assignment probabilities as a multinomial distribution computed from the component kernel probabilities at $\theta = \Theta$. Right: KDE resampled at $\theta = \Theta$.

Although not used in this work, this can be easily formulated for sampling circumferential location θ_j at a given intensity $I = \mathscr{I}$, given

$$p(I = \mathscr{I})_{c=1:C} = [\mathrm{TrG}(I = \mathscr{I}|\mu, \sigma, I_\mathrm{l}, I_\mathrm{u})_1, \ldots, \mathrm{TrG}(I = \mathscr{I}|\mu, \sigma, I_\mathrm{l}, I_\mathrm{u})_C]^T, \tag{S1.8}$$

then sampling:

$$\theta_j = \theta_j^0 + \theta_c, \quad \theta_j^0 \sim \mathrm{vM}(\theta|\nu, \kappa), \quad c \sim \mathrm{Mult}(c|\, p(I = \mathscr{I})_{c=1:C}). \tag{S1.9}$$

Computing the Wasserstein Distance

The Wasserstein distance is a distance metric between two distributions, in which the optimal transport plan to transform one distribution to the other is found. The q-th Wasserstein distance between probability distributions p_X and p_Y is defined as:

$$W_q(p_X, p_Y) = (\inf \mathbb{E}[d(X,Y)^q])^{1/q}, \quad X \sim p_X, Y \sim p_Y, \tag{S1.10}$$

where $d(X,Y)$ is a distance metric between random variables X and Y. In this work, given individual samples from X and Y, $x_i = [I_i^{(x)}, \theta_i^{(x)}]^T$ and $y_j = [I_j^{(y)}, \theta_j^{(y)}]^T$, the cylindrical space on which muscle activity exists is used as the basis of the distance metric:

$$d(x_i, y_j, \beta) = \sqrt{(I_i^{(x)} - I_j^{(y)})^2 + \alpha(\theta_i^{(x)}, \theta_j^{(y)}, \beta)^2}, \tag{S1.11}$$

where $\alpha(\theta_i^{(x)}, \theta_j^{(y)})$ is the minimum unit circumferential distance between points $\theta_i^{(x)}$ and $\theta_j^{(y)}$ scaled by β, defined as $\alpha(\theta_i^{(x)}, \theta_j^{(y)}, \beta) = \beta(((\theta_i^{(x)} - \theta_j^{(y)} + 0.5) \bmod 1) - 0.5)$. This distance in cylindrical space is illustrated in S5. Scaling the distance by β allows the Wasserstein distance calculation to favour one dimension over the other; in the context of the controllers presented in this work, this allows a controller to favour intensity changes over circumferential position changes (which is useful when robustness to muscle weakness is required), or to favour circumferential position changes over intensity changes (which is useful when robustness to electrode position changes is required). In this work, β is set to 3.0 empirically.

As detailed in[73], the Wasserstein distance between p_X and p_Y aims to find a flow $F = [f_{i,j}]$, between each sample x_i and y_j. When n samples are drawn from each distribution, the flow is that which minimises the following optimal transport cost:

$$\mathrm{WORK}(X,Y,F) = \sum_{i=1}^{n} \sum_{j=1}^{n} d_{i,j} f_{i,j}, \tag{S1.12}$$

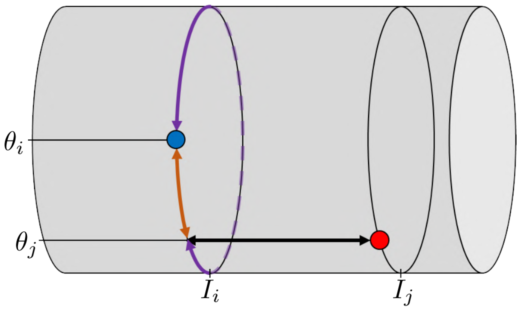

Figure S5. Measuring the distance between samples in cylindrical space. The distance between two points, $[I_i, \theta_i]^T$ and $[I_j, \theta_j]^T$, sampled from a cylindrical space. Alternative options to calculate the circumferential distance between the points are shown in orange and purple, respectively.

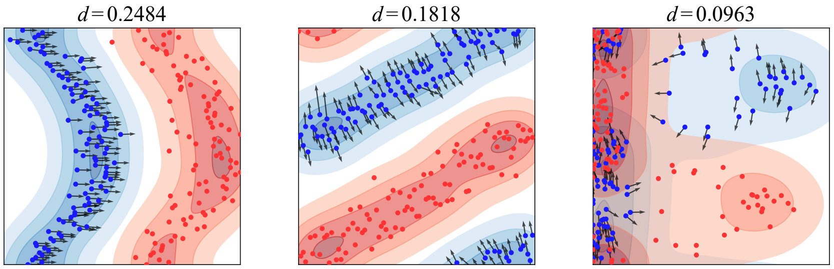

Figure S6. Illustrative example of the computed Wasserstein distance between two distributions. The Wasserstein distance is computed from datapoints resampled from two distributions (here coloured blue and red). Black arrows show the direction of point mass correspondences between sampled data from each distribution that produce the optimal transport solution. Left: offset in the linear intensity dimension. Middle: offset in the wrapped circumferential position dimensions. Right: 'toy' muscle activity data showing two independent muscles activating, while others remain inactive.

subject to:

$$f_{i,j} \geq 0 \qquad 1 \leq i \leq n, \quad 1 \leq j \leq n \tag{S1.13}$$

$$\sum_{j=1}^{n} f_{i,j} \leq 1 \qquad 1 \leq i \leq n \tag{S1.14}$$

$$\sum_{i=1}^{n} f_{i,j} \leq 1 \qquad 1 \leq j \leq n \tag{S1.15}$$

$$\sum_{i=1}^{n} \sum_{j=1}^{n} f_{i,j} = n. \tag{S1.16}$$

When the optimal flow has been found by solving the optimal transport problem, the Wasserstein distance is the normalised optimal work:

$$W_2(p_X, p_Y) = \frac{\sum_{i=1}^{n} \sum_{j=1}^{n} d_{i,j} f_{i,j}}{\sum_{i=1}^{n} \sum_{j=1}^{n} f_{i,j}}. \tag{S1.17}$$

For a multi-dimensional distribution, the number of calculations performed to solve the optimal transport problem rapidly grows with number of samples beyond computational feasibility. To reduce computational complexity, Sinkhorn divergences can be used to significantly improve calculation speed — this work is not intended to cover this approximation, and[75] provides a good overview of the practical methods used to calculate the Wasserstein distance.

An illustrative example of computing the Wasserstein distance between two sets of samples can be seen in Fig. S6, showing offsets in the intensity dimension (left), circumferential direction (middle), and both dimensions (right).

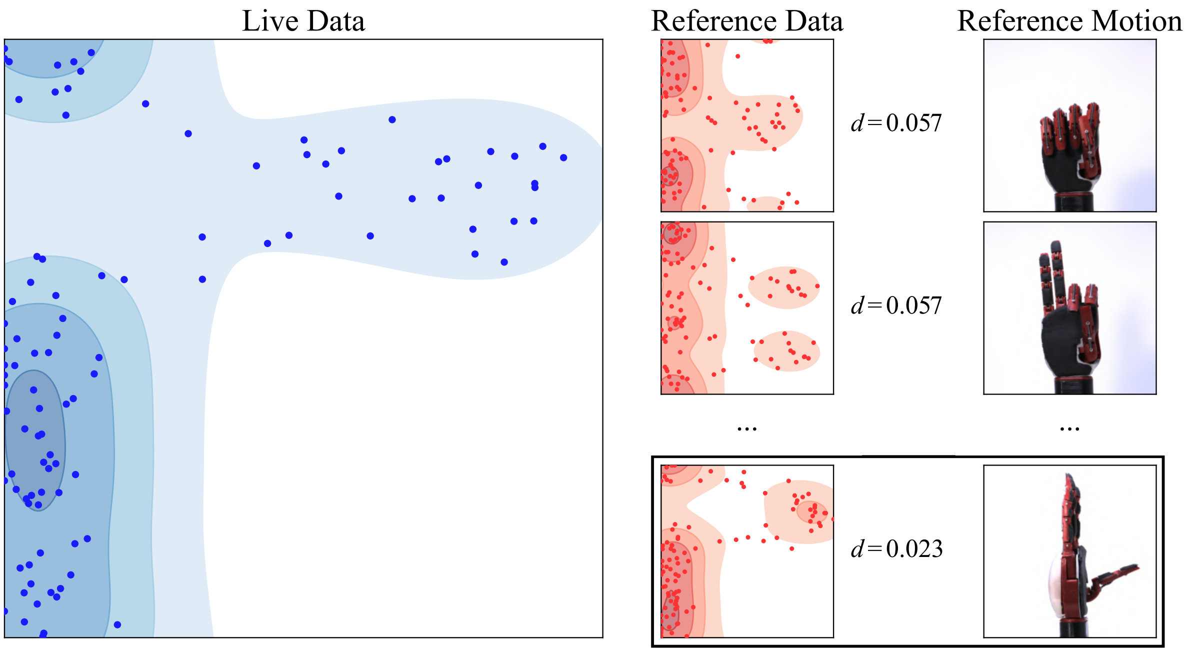

Figure S7. Illustrative example of the discrete control process. At each control step, the Wasserstein distance between the live data and each of the reference data are computed. If the Wasserstein distance falls below a threshold this reference is accepted as the correct motion.

Discrete Control

To achieve discrete control, a distance-based classifier is used to select the reference that is closest to the live distribution of EMG signals. Given R pre-recorded references, candidate reference a is selected:

$$a = \text{argmin}\{W_2(p_X, p_{Y_1}), \cdots, W_2(p_X, p_{Y_R})\}. \tag{S1.18}$$

Given a set of reference joint angles of the prosthesis $Q = [q_1, \ldots, q_R]^T$, the candidate action is only processed as the command action q at control step $k+1$ if it falls below a distance threshold d_l:

$$q(k+1) = \begin{cases} q_a & W_2(p_X, p_{Y_a}) < d_l \\ q(k) & \text{otherwise} \end{cases}. \tag{S1.19}$$

Because calculating the Wasserstein distance involves randomly sampling from each KDE, the distance between two identical distribution will not be exactly zero. The value of the distance threshold is chosen in order to ensure that the distance between two distributions is statistically significant. That is, knowing the variance of the kernel distribution making up the KDE, the distance threshold is calculated such that the p value of the one-tailed student's t-test score at this distance is less than 0.05 when two identical, but offset, sets of samples are used (i.e. when the Wasserstein distance between the two distributions is minimum).

Continuous Control

In this work, we approach continuous control from a generative approach, rather than a discriminative approach. Continuous control is achieved by considering how to reconstruct the distribution of live EMG signals from a linear combination of reference distributions. Given that EMG signals can be modelled as the output of a linear, time invariant system[63], the resultant intensity of multiple active motor units is the superposition of their individual outputs. Given that reference distributions are recorded by activating independent groups of muscles, the observed intensity of live EMG at a given circumferential position $\theta = \theta_n$ around the forearm can be approximated as a weighted linear combination of each reference intensity at that location:

$$\hat{I}\Big|_{\theta=\theta_n} = \sum_{r=1}^{R} w_r I_r \Big|_{\theta=\theta_n}, \tag{S1.20}$$

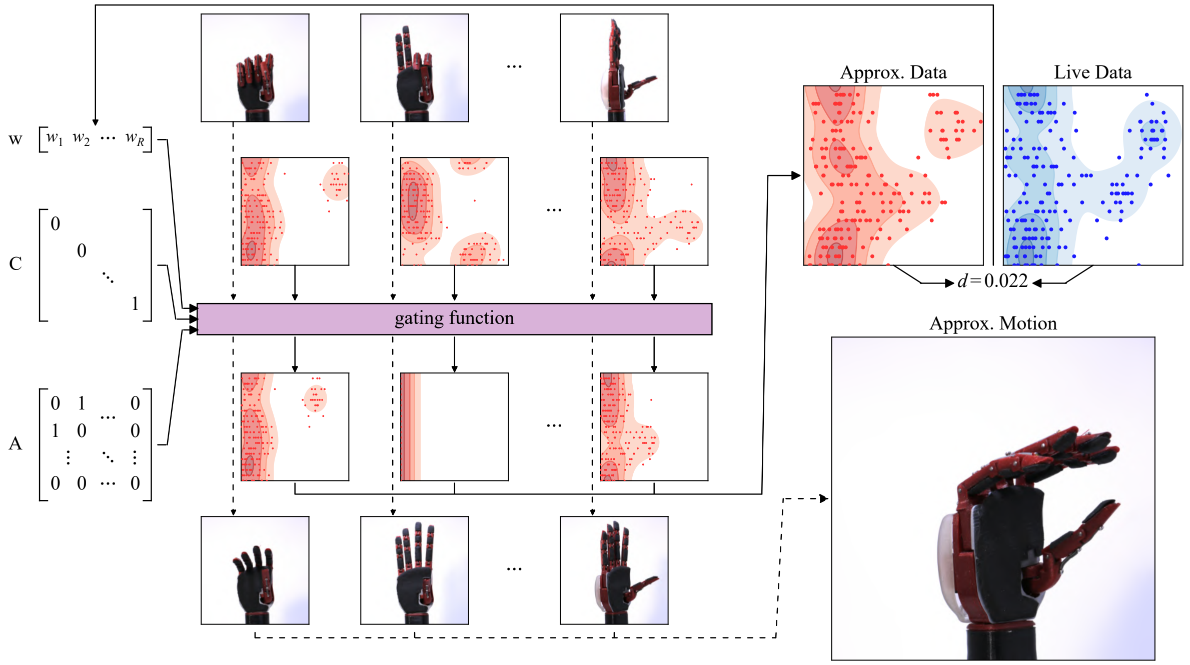

Figure S8. Illustrative example of the continuous control process. At each control step, an approximation of the live data is formed by performing a weighted sum of the reference data in the intensity dimension that minimises the Wasserstein distance between the approximate and live data. A gating function is used to prevent antagonistic pairs of reference data from activating simultaneously.

where w_r is the weighting applied to reference r. Repeating this process at many circumferential positions $\Theta = [\theta_1, \ldots, \theta_N]^T$ yields a series of approximate intensities that follow the conditional distribution

$$p_{\hat{I}|\theta=\Theta} = p(\hat{I}|\theta = \Theta, w), \tag{S1.21}$$

where $w = [w_1, \ldots, w_R]^T$ is the vector of weights applied to each reference. The resultant distribution, $p_{\hat{X}}$, is equal to:

$$p_{\hat{X}} = p(\hat{I}|\theta = \Theta, w)\, p(\theta = \Theta). \tag{S1.22}$$

To measure how well these weightings approximate the live data, the Wasserstein distance between the live distribution and the approximate distribution, conditionally sampled at $\theta = \Theta$, can then be used as a loss function:

$$\mathscr{L}_W = W_2(p_X, p_{\hat{X}}). \tag{S1.23}$$

The approximate distribution $p_{\hat{X}}$ is formed via the summation of many random variables (each reference intensity I_r is a random variable composed of many KDE components, each with their own intensity), meaning the resultant probability density function is the convolution of each of the corresponding probability density functions. This approximate distribution must then be sampled in order to calculate the Wasserstein distance. It is computationally infeasible to perform these calculations at every control step, so an alternative approach is considered. It is equivalent mathematically to, rather than compute the resultant probability density function then conditionally sample it, conditionally sample each reference probability density function then perform the weighted sum of these samples:

$$\hat{I}\Big|_{\theta=\Theta} = \sum_{r=1}^{R} w_r I_r \Big|_{\theta=\Theta}, \quad \hat{I} \sim p_{\hat{I}|\theta=\Theta}, \quad I_r \sim p_{I_r|\theta=\Theta}. \tag{S1.24}$$

If the reference library contains references involving muscles that form an antagonistic pair, then only one reference from the pair should contribute to the resultant distribution at any time. To ensure that this is the case, a gating function is used, such

that when one reference in the pair is high, the other reference is suppressed, and vice versa. The gating function chosen is an inverted sigmoid function, which outputs zero when w_i is high, and one when w_i is low:

$$g_r(w_r) = 1 - \frac{1}{1+\exp\left(-k(w_r - w_0)\right)}. \tag{S1.25}$$

For a reference library that is comprised of a combination of antagonistic references and complementary references, two flagging matrices are used to identify allowable combinations of samples. The first is a antagonistic flag matrix, $A \in \mathbb{R}^{R \times R}$, which is zero-filled with unit flags at entries A_{r_1,r_2} and A_{r_2,r_1} to indicate that references r_1 and r_2 make an antagonistic pair. The second is an isolated flag matrix, $C \in \mathbb{R}^{R \times R}$, which is also zero-filled, but with unit flags at entries C_{r_1,r_1} indicating that reference r_1 is isolated and does not form an antagonistic pair with any other reference in the library. The final weight of reference r_1 is equal to:

$$\bar{w}_{r_1} = \Big(C_{r_1,r_1}(1 - g_{r_1}(w_{r_1})) + \sum_{r_2=1}^{R} A_{r_1,r_2} g_{r_2}(w_{r_2})\Big) w_{r_1}. \tag{S1.26}$$

Note that C_{r_1,r_1} is multiplied by $1 - g_{r_1}(w_{r_1})$ in order to balance the gradients of the antagonistic flag sigmoid. In vector notation where $g = [g(w_1), \ldots, g(w_R)]^T$:

$$\bar{w} = \mathrm{diag}(C(1-g) + Ag)w. \tag{S1.27}$$

At each control step, the weighting of each component distribution is optimised using the gradient of the Wasserstein distance between the approximate and live distributions. To warm start the optimisation, the optimised weightings from the previous control step are used to initialise the weightings of the current control step. Convergence to an optima is not guaranteed within the control step time period; in this case the most recent weightings are taken as the solution.

To convert computed weights to an output motion, each reference is mapped to a set of prosthesis joint angles $Q = [q_1, \ldots, q_R]^T$. Complementary references are mapped to independent motions; for example, the motion of the wrist and fingers. Antagonistic references are mapped to overlapping motions such that they cannot be activated at the same time; for example, tripod grip and power grip. The target position of the prosthesis $\hat{q}$ at the next control step, $k+1$, is the weighted sum of each reference, exponentially smoothed with its current target position:

$$\hat{q}(k+1) = \alpha \sum_{r=1}^{R} w_r q_r(k+1) + (1-\alpha)q(k), \tag{S1.28}$$

where α is the smoothing factor, chosen heuristically as 0.3. A summary block diagram of the entire continuous control process is shown in Fig. S8.

The most computationally intensive step in the control system is to calculate the Wasserstein distance. In the discrete controller, the Wasserstein distance must be calculated once for each reference at each control step. In the continuous controller, the distance calculation is only performed between the approximated data an the live data, however this is repeated in the inner weighting optimisation loop for each gradient descent step. In this work, a single gradient descent step is used per control step, meaning the controller is able to run at a rate of approximately 20 Hz, with higher rates expected with dedicated embedded hardware in the future.

S2 Full Implementation Details of the Haptic Feedback Armband

Proprioceptive feedback is rendered to the user to represent a combination of the position and force aspects of proprioception of the fingers and wrist of the OLYMPIC hand, which is equipped with sensors to detect finger position and force[65]. A modified version of the haptic feedback armband presented in[52] is used, rendering three modalities of feedback, each targeting a specific mechanoreceptor in the skin: normal displacement targets Merkel corpuscles, tangential position targets Meissner corpuscles, and vibration targets Pacinian corpuscles.

The modes of feedback, tangential position, normal displacement, and vibration, render proprioceptive finger position, finger force, and wrist position, respectively, and are illustrated in Fig. S9. For finger f, tangential position feedback x_f is given to the user as a direct mapping of motor position θ_f:

$$x_f = \theta_f. \tag{S2.1}$$

Normal displacement feedback z_f, is rendered to the user as the excess static force in the finger motor:

$$z_f = \begin{cases} \tau_f - \hat{\tau}_f(\theta_f) & \dot{\theta}_f < \dot{\theta}_l \\ 0 & \text{otherwise,} \end{cases} \tag{S2.2}$$

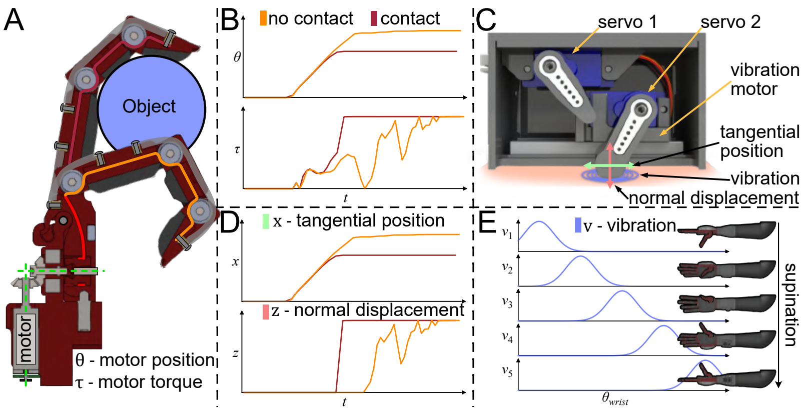

Figure S9. The motion of a the prosthetic hand and its corresponding sensory feedback modes. (*A*) A finger of the prosthetic hand shown freely closing (lighter, orange) and contacting an object (darker, purple). (*B*) Corresponding trajectories of the motor position θ and torque τ of the finger. (*C*) A section view of the corresponding haptic feedback module showing each mode of sensory feedback - tangential position (green), normal displacement (red), and vibration (blue). (*D*) The tangential position feedback x, representing finger position, and normal displacement feedback z, representing finger force, corresponding to the motor trajectories in (*B*). (*E*) Vibration feedback $v_1 - v_5$ of five modules, providing proprioceptive feedback of wrist pronation-supination.

where $\hat{\tau}_f(\theta_f)$ is the expected value of the motor force τ_f at motor position θ_f, calculated according to[66], and $\dot{\theta}_l$ is a motor speed threshold to prevent normal displacement from being applied prematurely.

Vibration feedback from multiple armband modules is used to provide proprioceptive position feedback from the wrist. Vibration feedback produced by each module v_f, is produced as a combination of Gaussian curves along the pronation/supination angle of the wrist, θ_{wrist}, equally spaced by $\Delta\theta$:

$$v_f \propto \exp\left(-\frac{(\theta_{\text{wrist}} - f\Delta\theta)^2}{2(\frac{1}{3}\Delta\theta)^2}\right), \tag{S2.3}$$

where the standard deviation of the Gaussian, $\frac{1}{3}\Delta\theta$, is chosen such that individual haptic modules reach their peak vibration intensity without overlap from other modules.

S3 Full Setup Details of the Clinical Dexterity Assessments

Box and Blocks Test

In the Box and Bocks Test, shown in Fig. S10, the participant is asked to move as many cubes in 1 minute from one box to another, over a partition. The Box and Blocks Test kit used in this study was a modified flat-pack version of the original kit detailed in[53]. The outer box is dimensioned 53.7 cm × 25.4 cm × 8.5 cm, with a 15.2 cm high partition dividing the outer box in two. 150 cubes with side-length 2.5 cm are used.

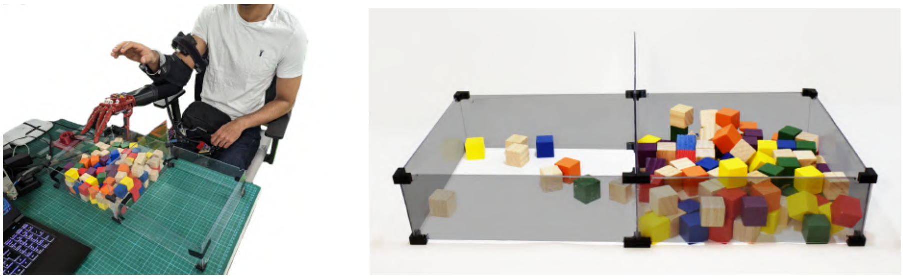

Figure S10. Box and Blocks Test. Participants must transfer as many blocks as possible from one half of the box to the other in 1 minute. Left: a participant without limb difference completing the Box and Blocks Test. Right: assembled Box and Blocks Test.

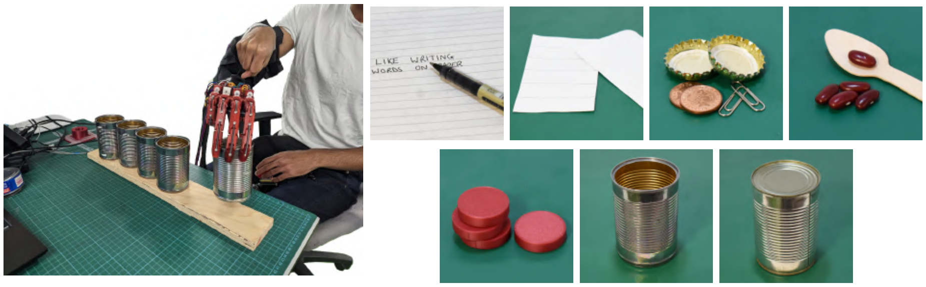

Figure S11. Jebsen-Taylor Hand Function Test. Left: a participant without limb difference completing the Jebsen-Taylor Hand Function Test. Right: Tasks from the Jebsen-Taylor Hand Function Test, from left to right, top row: (J1) writing, (J2) simulated page turning, (J3) small objects, (J4) simulated feeding. From left to right, bottom row: (J5) stacking checkers, (J6) large, light objects, and (J7) large, heavy objects.

Jebsen-Taylor Hand Function Test

The Jebsen-Taylor Hand Function Test is made up of 7 tasks, shown in Fig. S11: (J1) writing, (J2) simulated page turning, (J3) small objects, (J4) simulated feeding, (J5) stacking checkers, (J6) large, light objects, and (J7) large, heavy objects. The Jebsen-Taylor Hand Function Test kit used in this study was a recreated version of the standard kit, originally presented in[54]. The original kit contains an activity board comprised of two parts: a main board, and a vertical sub-board. The main board measures 28 cm $\times$ 103 cm, and has lines marked width-ways at 10 cm intervals along its length. The vertical sub-board measures 5 cm $\times$ 50 cm and has lines marked along its edge at 5 cm intervals, offset initially by 2.5 cm, and is located such that the face of the sub-board that is facing the participant is along the mid-line of the main board. In this work, only the vertical sub-board is used. The sub-board is lay flat, and acts as the main board for tasks J5, J6, and J7. For task J4, simulated feeding, the edge of the sub-board is used.

The components used in each task are as follows:

- J1 Writing: a sheet of paper and a ball-point pen.
- J2 Simulated Page Turning: 5 paper cards, each measuring 7.6 cm $\times$ 12.7 cm.
- J3 Small Objects: 2 paperclips, 2 bottle caps, and 2 one-pence coins, and 1 empty can (from J6).
- J4 Simulated Feeding: a spoon and 5 dried kidney beans.
- J5 Stacking Checkers: 4 checkers, each measuring 2.5 cm in diameter and 0.8 cm in thickness.
- J6 Large, Light Objects: 5 empty cans.
- J7 Large, Heavy Objects: 5 full cans, weighing approximately 450 g.

Supplementary Figures

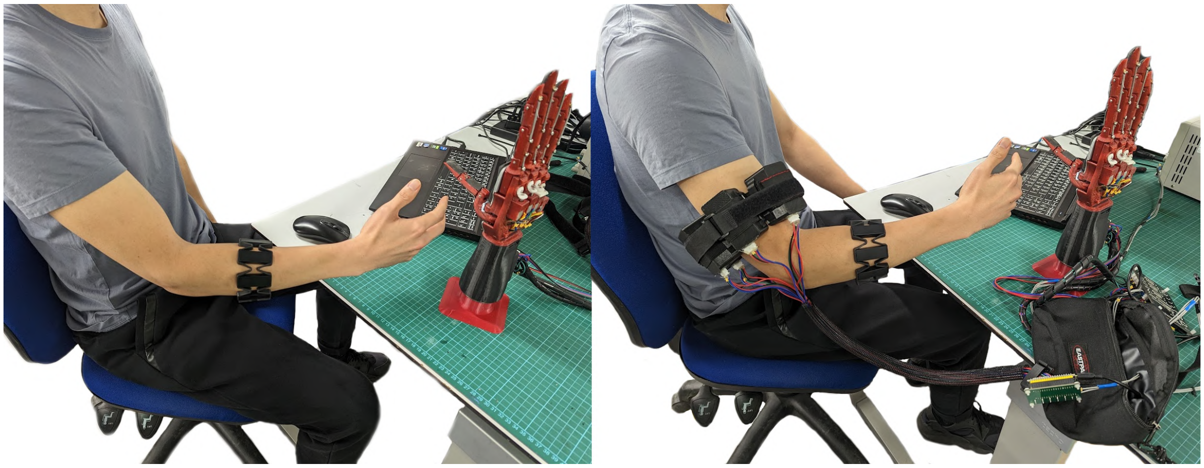

Stages 1 & 2

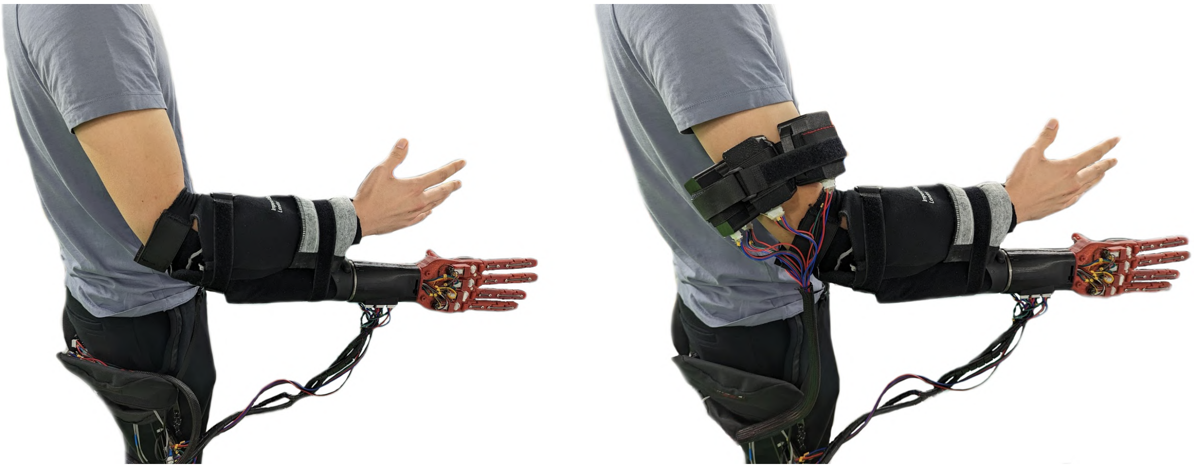

Stage 3

Figure S12. Experimental setup for participants without limb difference. Experimental setup for participants without limb difference for stages 1 and 2 (top) and 3 (bottom), in open-loop conditions (left) and closed-loop conditions (right).

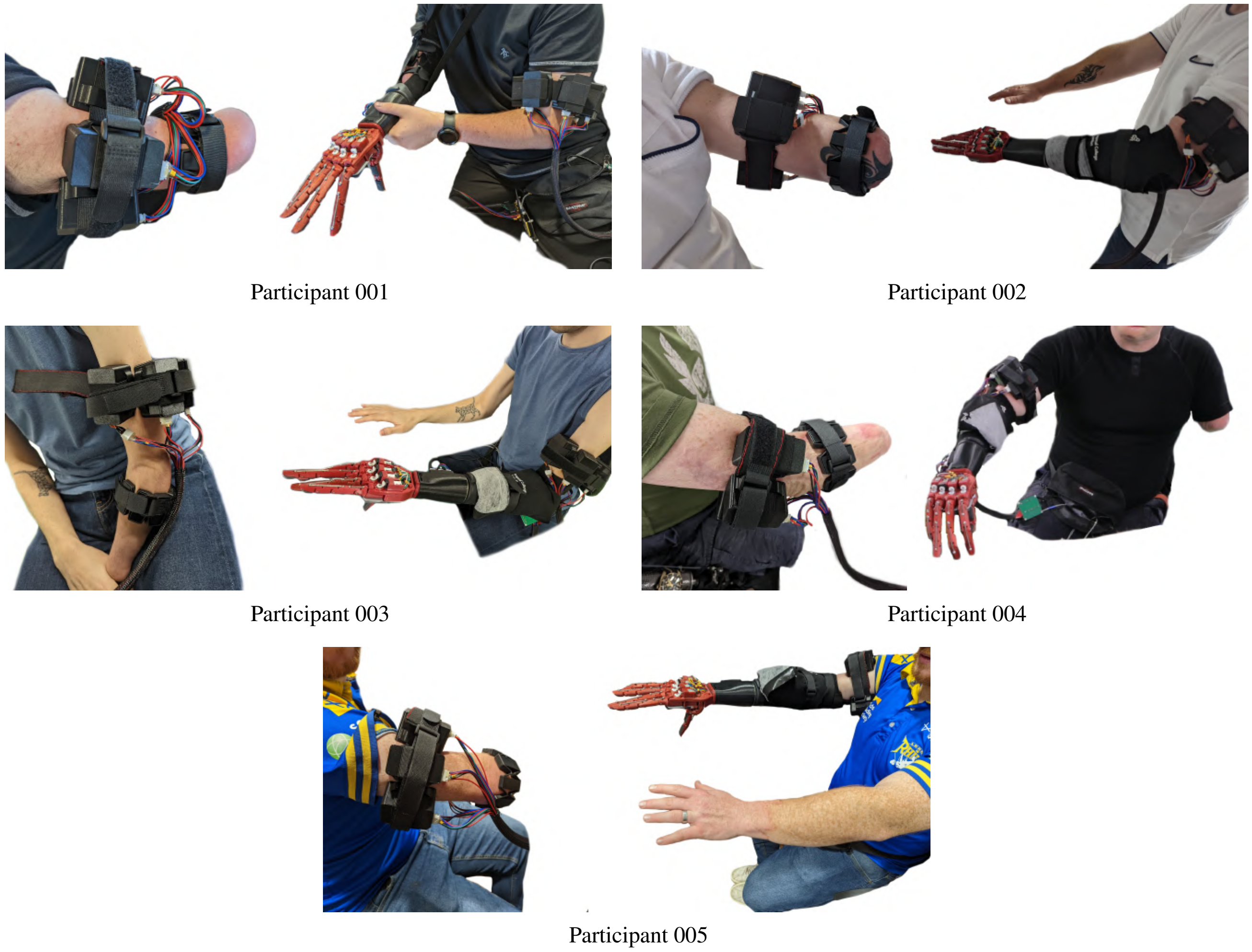

Figure S13. Experimental setup for each participant with limb difference. Experimental setup for each participant with limb difference for stages 1 and 2 (left), and 3 (right). During stages 1 and 2, participants wore only the electrode armband and haptic feedback armband, while the prosthetic hand was desk-mounted. During stage 3, participants also donned the prosthetic hand. Note that participant 001 used an elastic sling to support the prosthesis due to having a short residual limb, and wore the haptic feedback armband on the opposing arm during stage 3 due to space constraints.

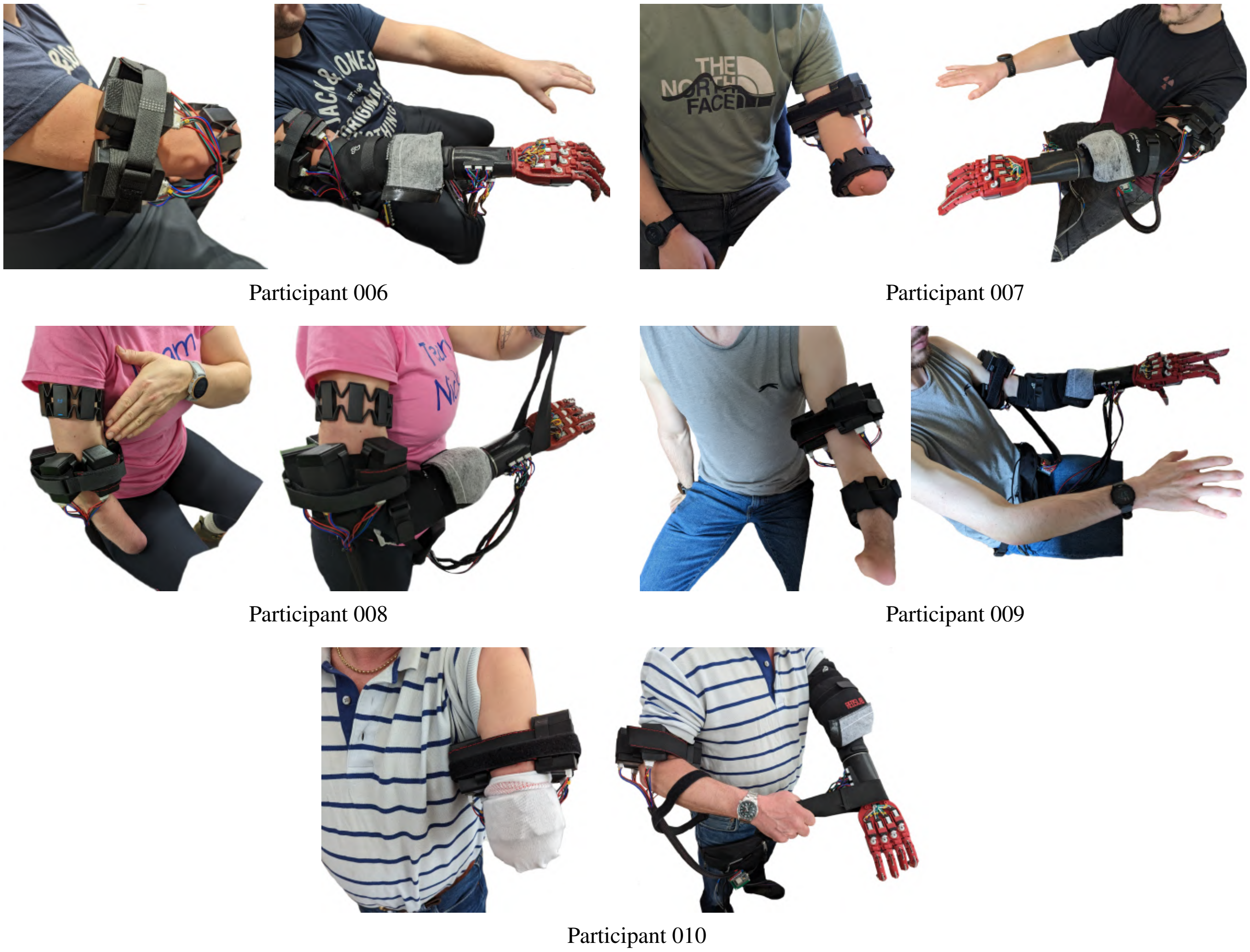

Figure S13. (Continued) Experimental setup for each participant with limb difference. Experimental setup for each participant with limb difference for stages 1 and 2 (left), and 3 (right). During stages 1 and 2, participants wore only the electrode armband and haptic feedback armband, while the prosthetic hand was desk-mounted. During stage 3, participants also donned the prosthetic hand. Note than an elastic belt held by the opposing arm to support the prosthesis was used for participants 007 and 010 due to having a short residual limb, and participant 008 due to having partial paralysis of the right arm and shoulder. Participant 008 had partial muscle reinnervation following nerve transplant, meaning EMG signals were collected from the reinnervated region of their upper arm.

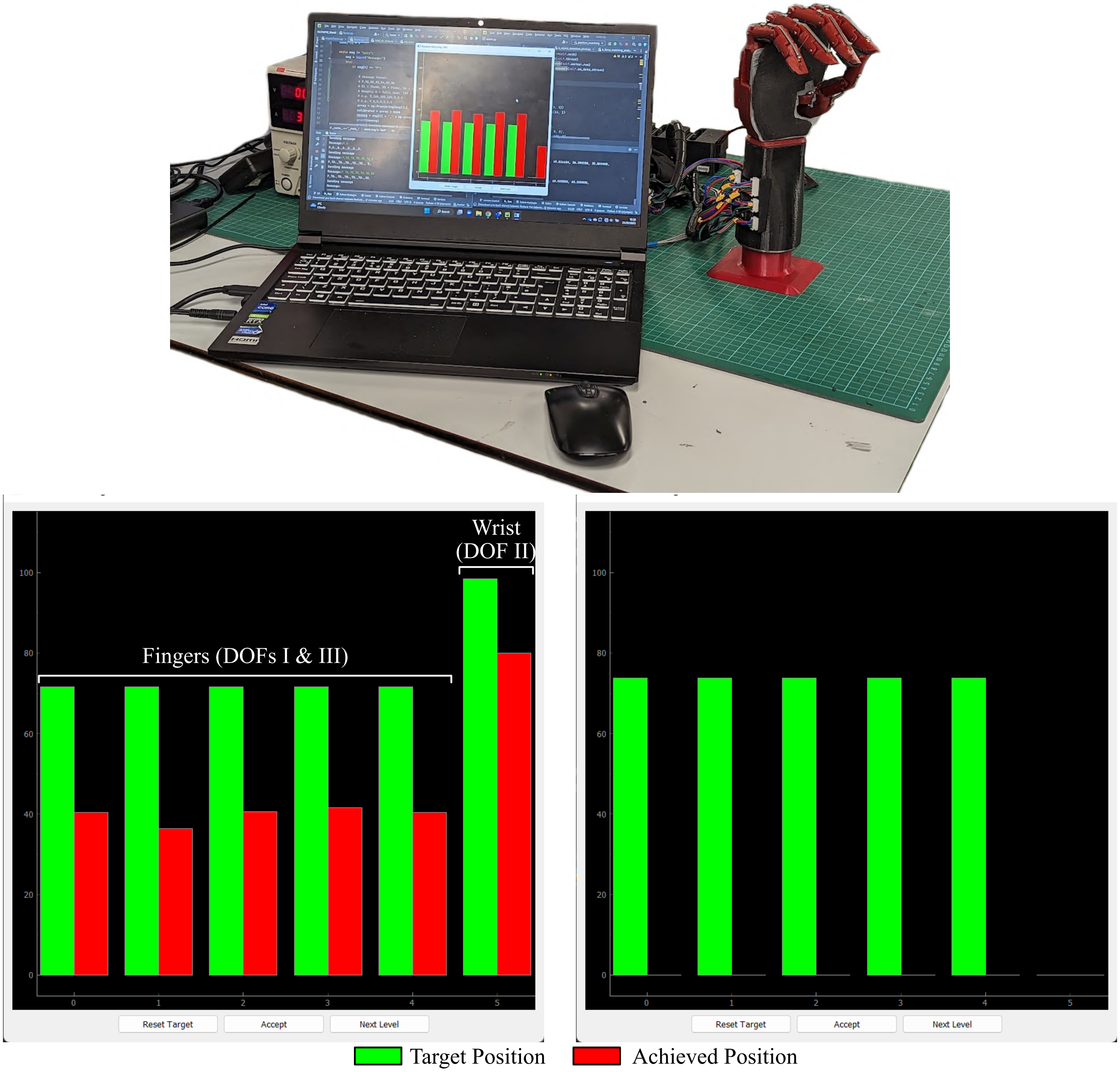

Figure S14. Setup of the position matching experiments. Position matching experiments were performed with the prosthesis desk-mounted (top). During training, participants were exposed to explicit visual feedback showing the position of the 5 finger motors and 1 wrist motor of the prosthetic hand in the form of the red bars showing achieved position on the graphical user interface (bottom left). During testing, the red bars were removed, leaving only the green bars showing target position (bottom right).

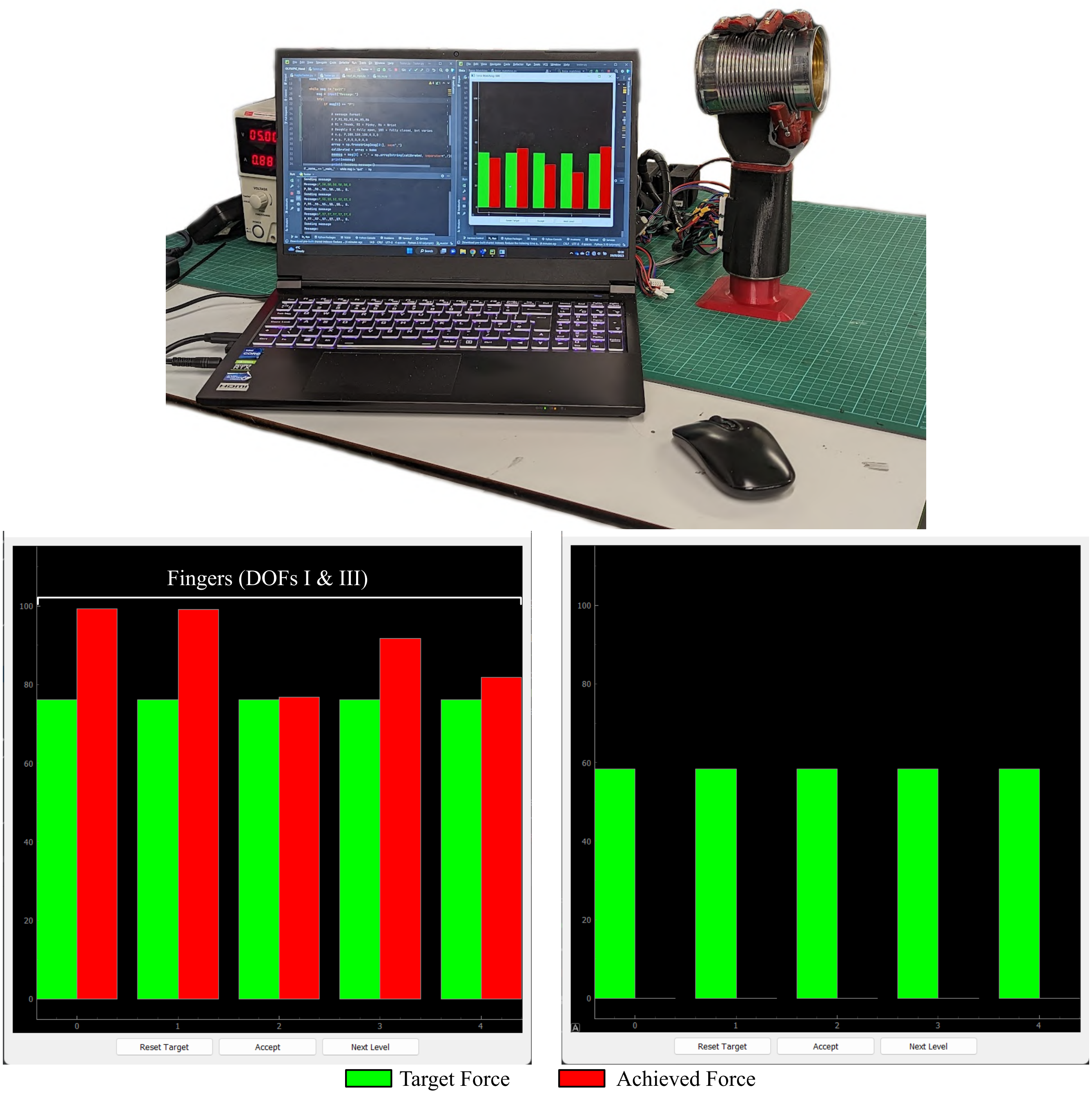

Figure S15. Setup of the force matching experiments. Force matching experiments were performed with the prosthesis desk-mounted (top).During training, participants were exposed to explicit visual feedback showing the force applied by the 5 finger motors in the prosthetic hand in the form of the red bars showing achieved force on the graphical user interface (bottom left). During testing, the red bars were removed, leaving only the green bars showing target force (bottom right).

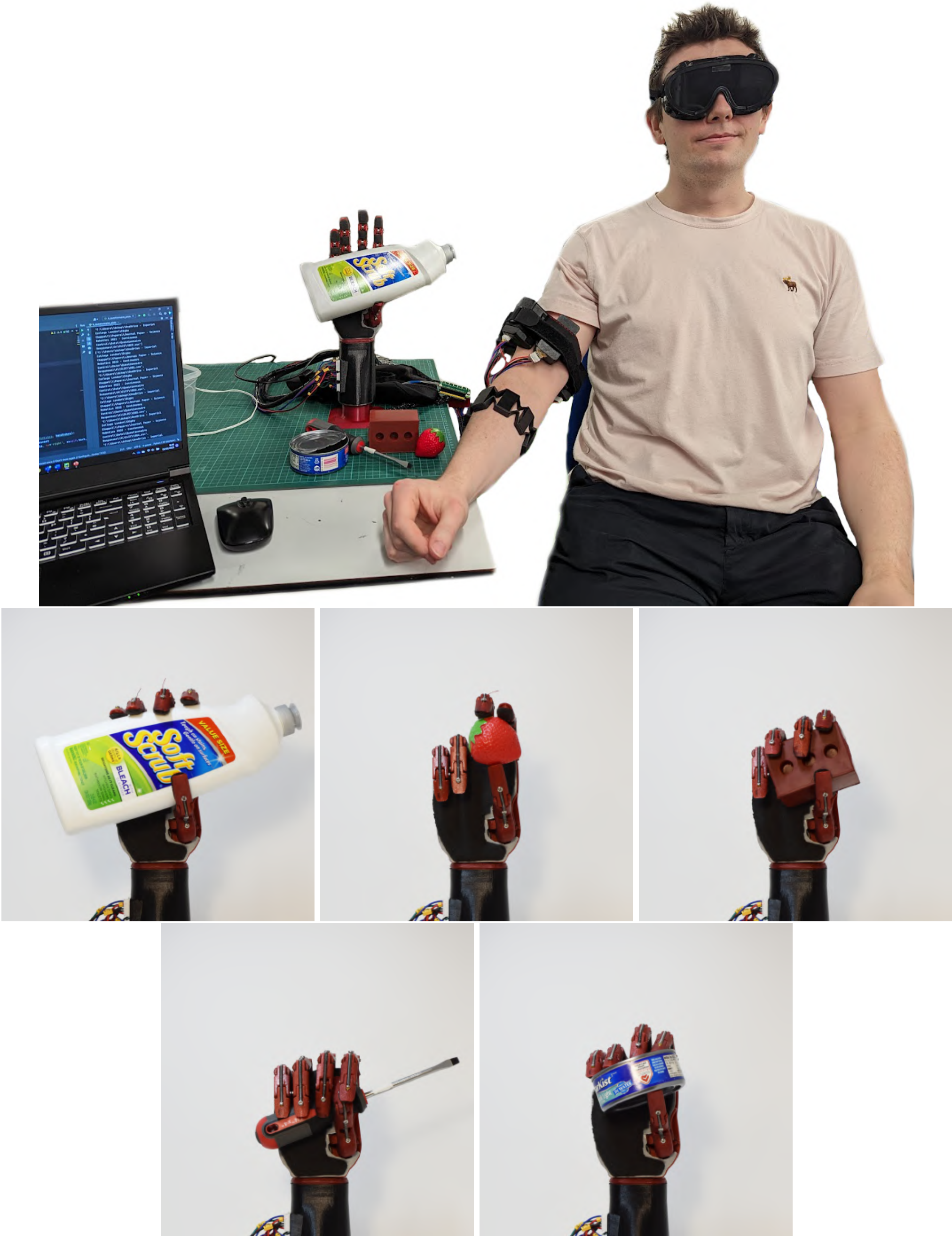

Figure S16. Setup of the object identification experiments. During training, participants were able to see the hand grasp each object twice. During testing (top), participants wore a blindfold and each object was grasped five times in a random order. The five objects taken from the YCB object and model set used for object identification experiments (middle row): a bleach bottle, a strawberry, a soft brick, a screwdriver, and a tuna can.

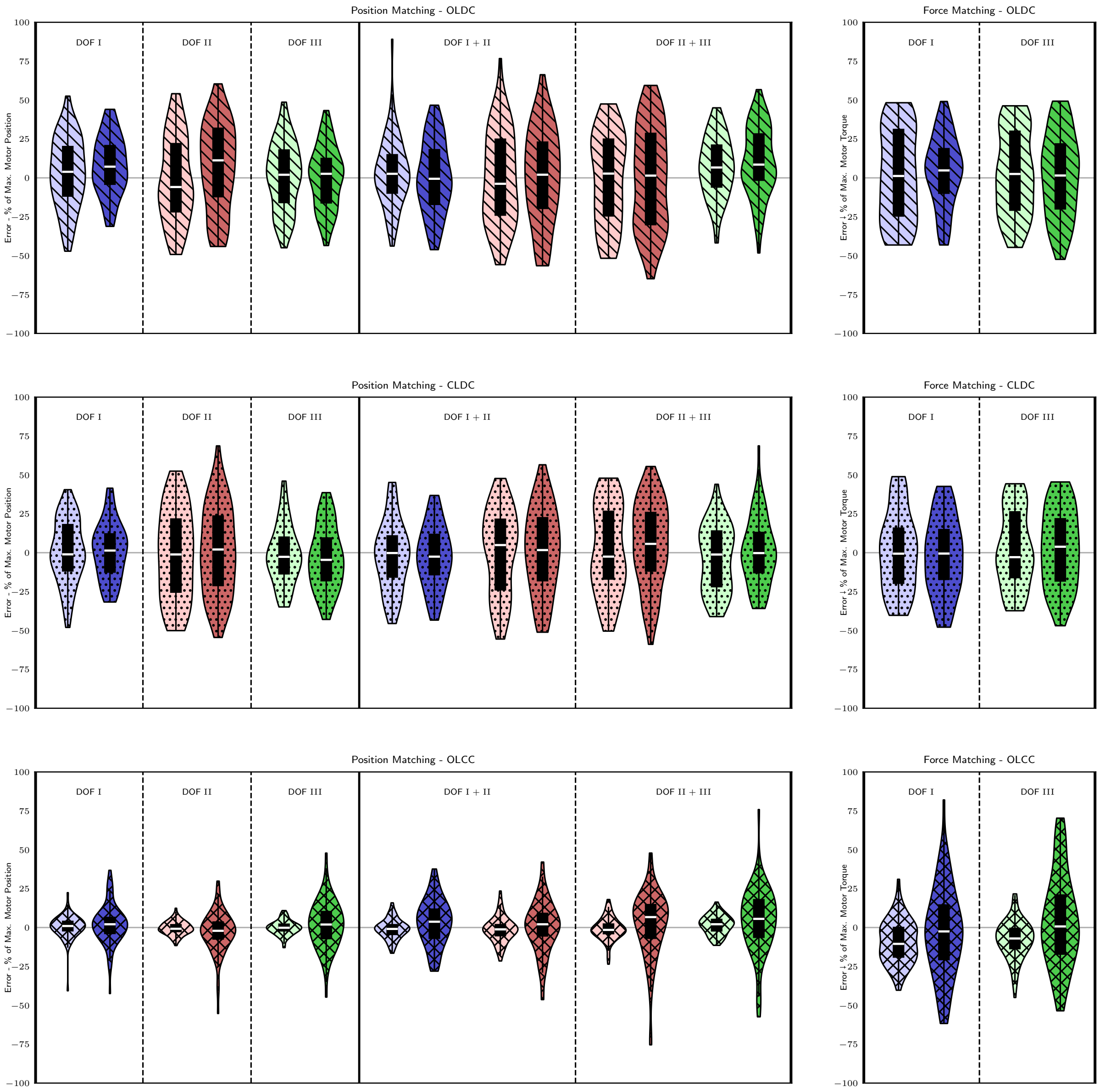

Figure S17. Matching performance experimental results of groups of participants without limb difference. Participants were asked to match target positions in sets of increasing complexity in terms of the number of degrees of freedom required, then were asked to match target forces with each grasping degree of freedom. Left: Position matching performance of single and dual degree of freedom motions, performed with no prior training starting from 1 degree of freedom motions, working to 2 degree of freedom motions in the order shown. Right: Force matching performance of single degree of freedom grasps. Statistical significance of Kolmogorov-Smirnov tests shown at $*p < 0.05$, $**p < 0.01$, and $***p < 0.005$ levels.

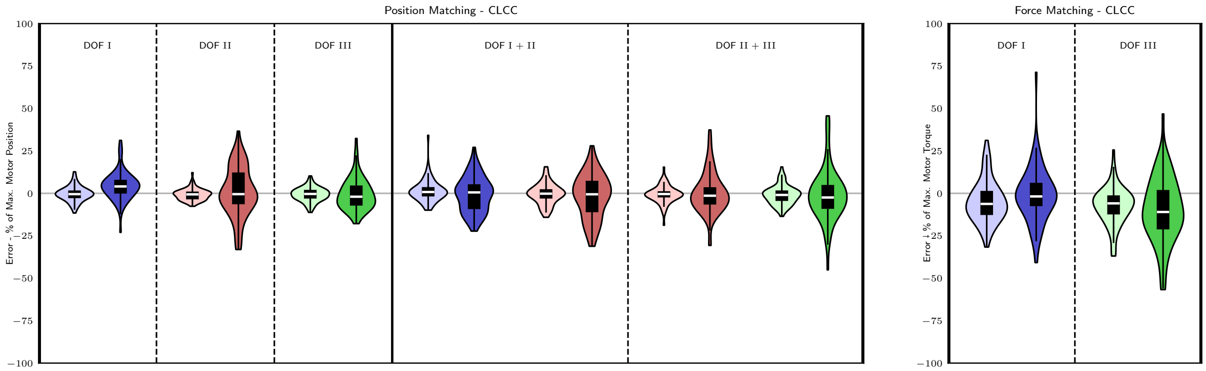

Figure S17. (Continued) Matching performance experimental results of groups of participants without limb difference. Participants were asked to match target positions in sets of increasing complexity in terms of the number of degrees of freedom required, then were asked to match target forces with each grasping degree of freedom. Left: Position matching performance of single and dual degree of freedom motions, performed with no prior training starting from 1 degree of freedom motions, working to 2 degree of freedom motions in the order shown. Right: Force matching performance of single degree of freedom grasps. Statistical significance of Kolmogorov-Smirnov tests shown at $*p < 0.05$, $**p < 0.01$, and $***p < 0.005$ levels.

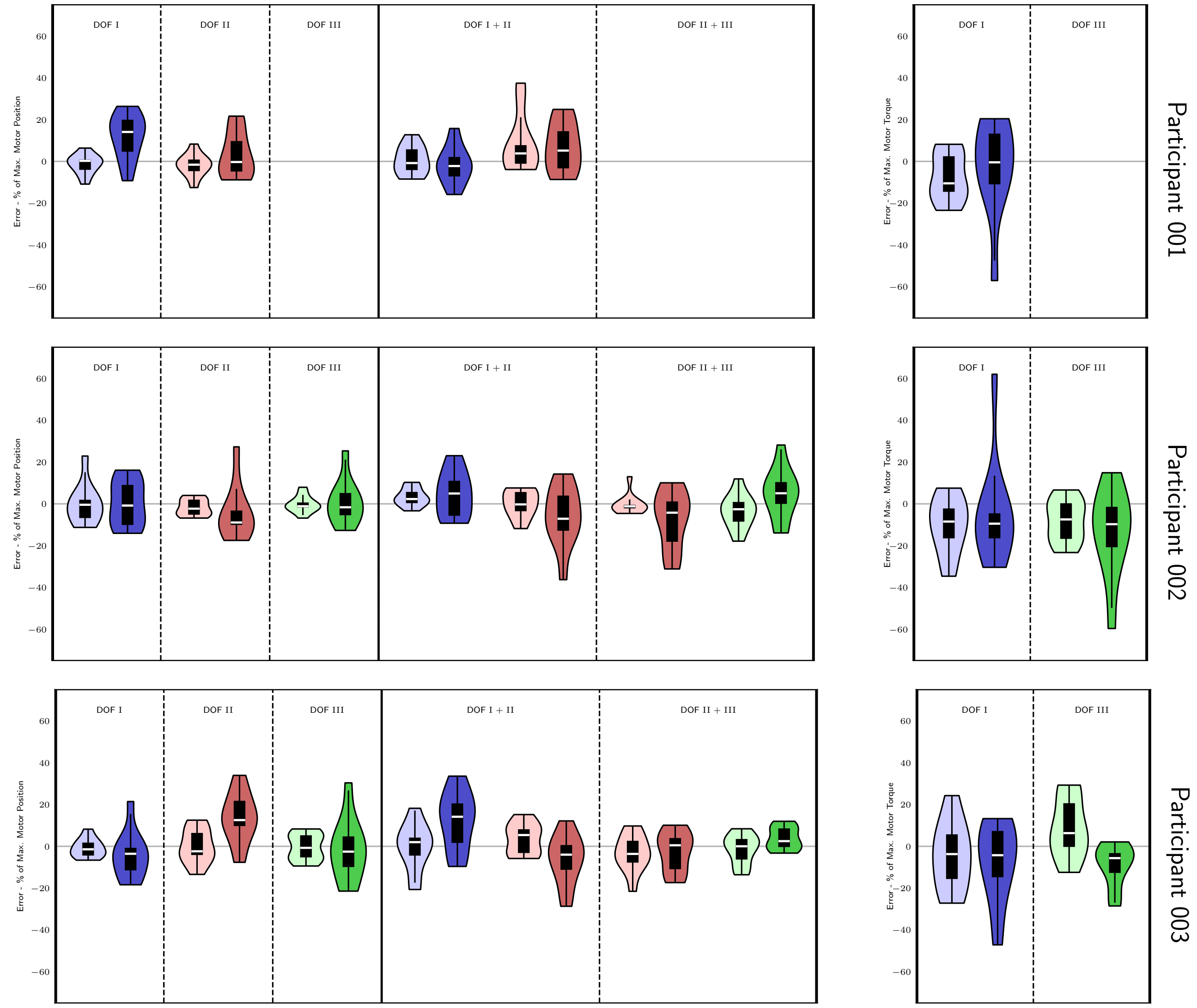

Figure S18. Matching performance results of participants with limb difference. Participants were asked to match target positions in sets of increasing complexity in terms of the number of degrees of freedom required, then were asked to match target forces with each grasping degree of freedom. Left: Position matching performance of single and dual degree of freedom motions, performed with no prior training starting from 1 degree of freedom motions, working to 2 degree of freedom motions in the order shown. Right: Force matching performance of single degree of freedom grasps. Note: Participants 001 and 005 only controlled DOF 1 and 2 of the prosthetic hand.

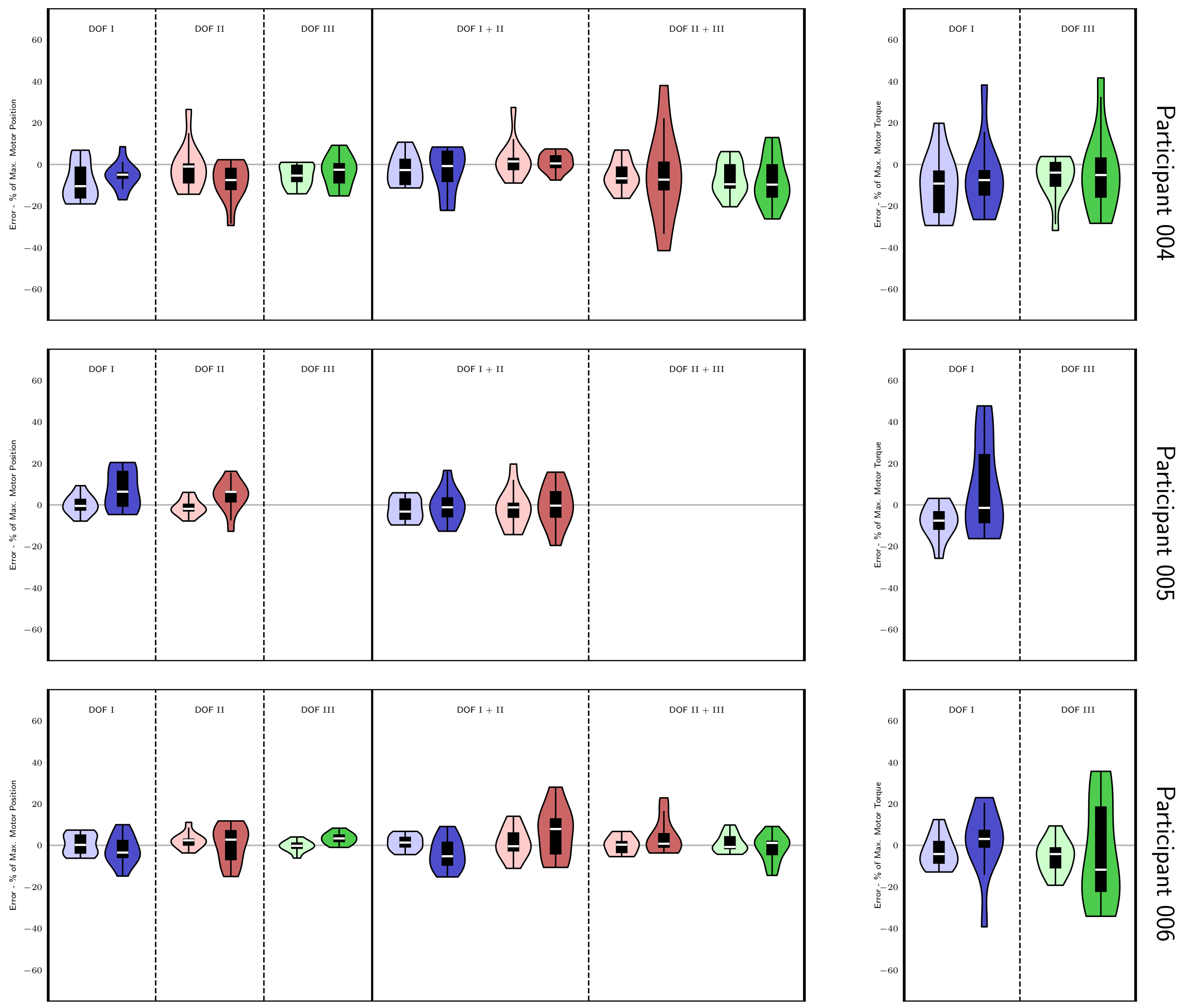

Figure S18. (Continued) Matching performance results of participants with limb difference. Participants were asked to match target positions in sets of increasing complexity in terms of the number of degrees of freedom required, then were asked to match target forces with each grasping degree of freedom. Left: Position matching performance of single and dual degree of freedom motions, performed with no prior training starting from 1 degree of freedom motions, working to 2 degree of freedom motions in the order shown. Right: Force matching performance of single degree of freedom grasps. Note: Participants 001 and 005 only controlled DOF 1 and 2 of the prosthetic hand.

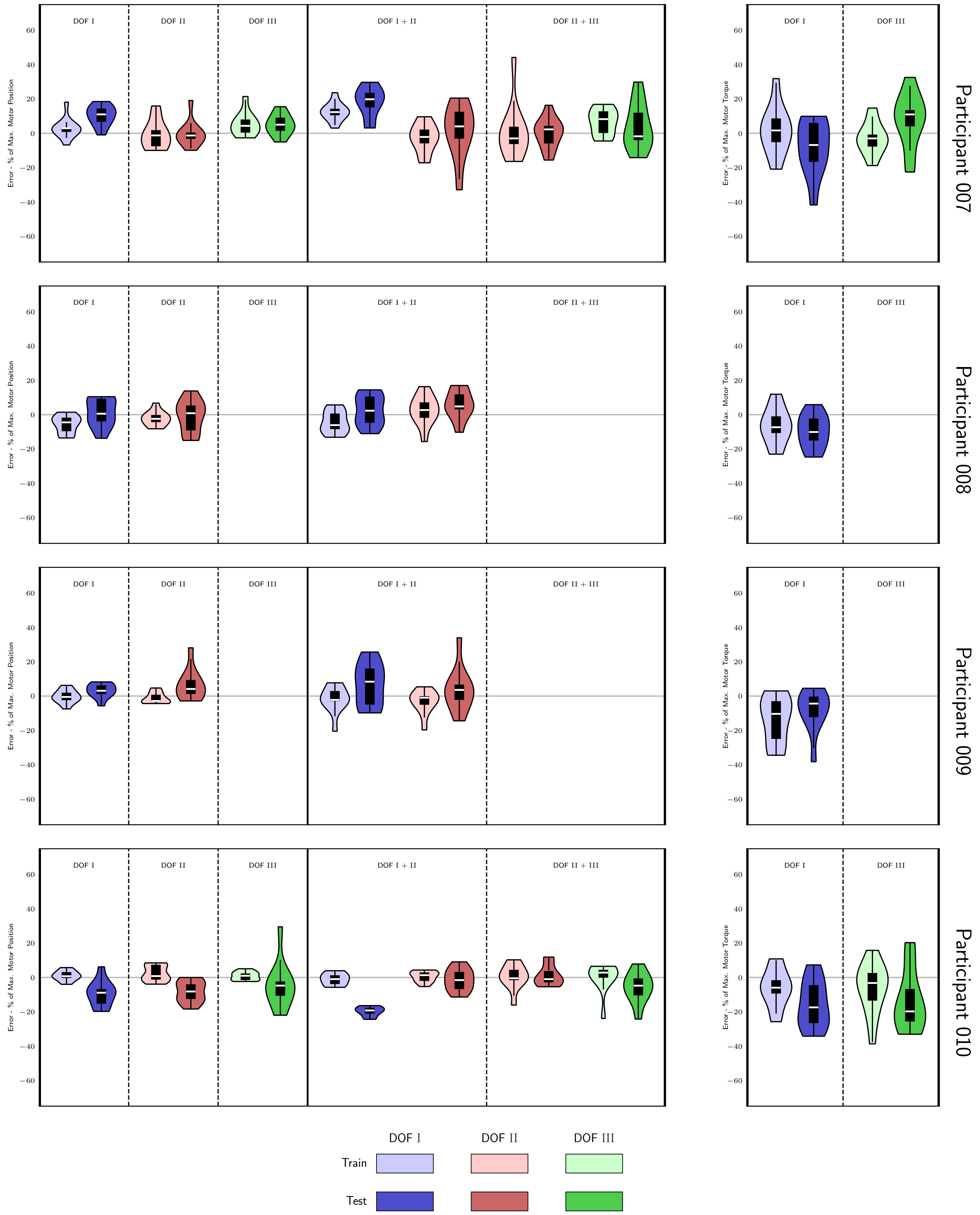

Figure S18. (Continued) Matching performance results of participants with limb difference. Participants were asked to match target positions in sets of increasing complexity in terms of the number of degrees of freedom required, then were asked to match target forces with each grasping degree of freedom. Left: Position matching performance of single and dual degree of freedom motions, performed with no prior training starting from 1 degree of freedom motions, working to 2 degree of freedom motions in the order shown. Right: Force matching performance of single degree of freedom grasps. Note: Participants 001 and 005 only controlled DOF 1 and 2 of the prosthetic hand.

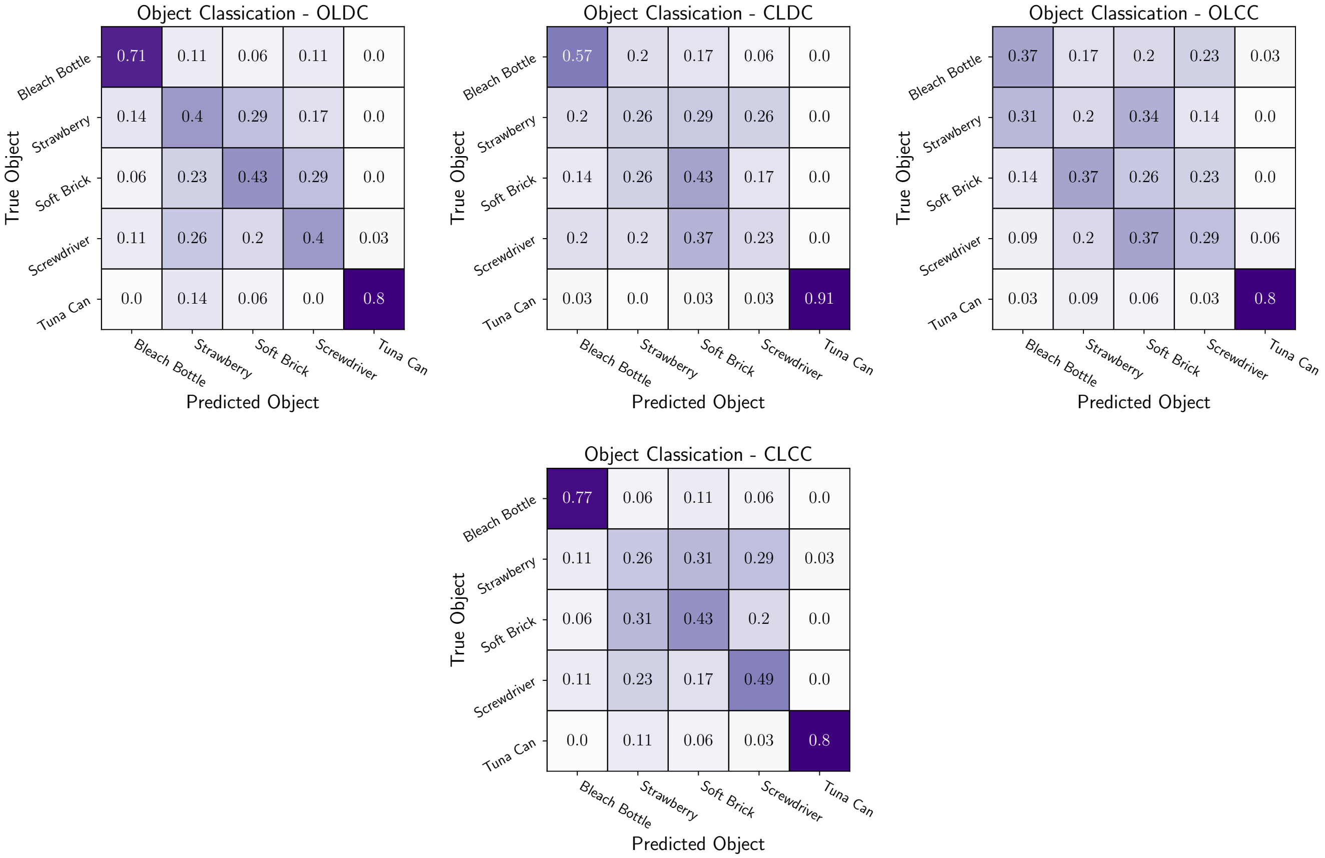

Figure S19. Object classification results of groups of participants without limb difference. Confusion matrices of groups of participants without limb difference classifying 5 objects from sensory feedback provided by the haptic feedback armband and incidental (audio) feedback.

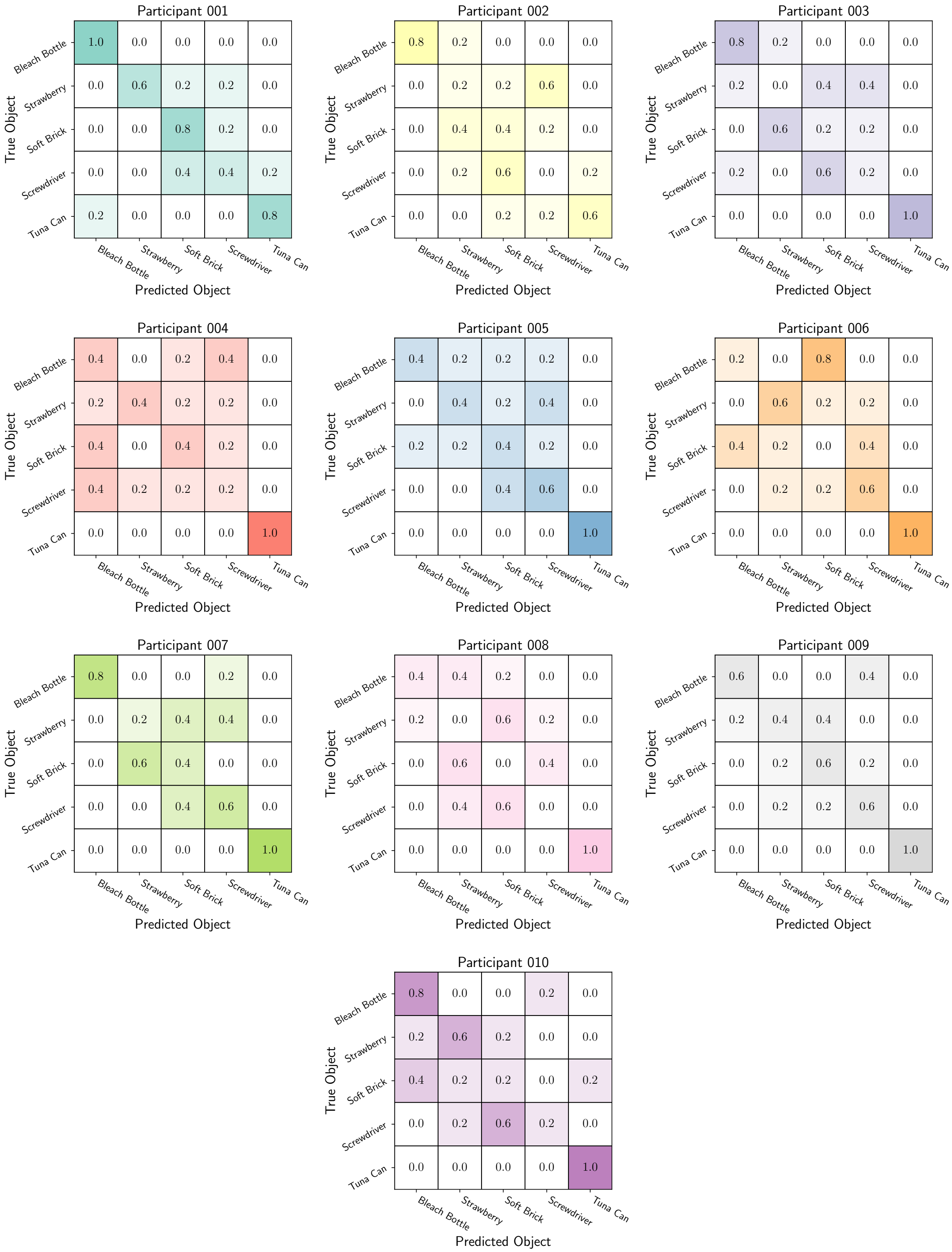

Figure S20. Object classification results of participants with limb difference. Confusion matrices of participants with limb difference classifying 5 objects from sensory feedback provided by the haptic feedback armband and incidental (audio) feedback.

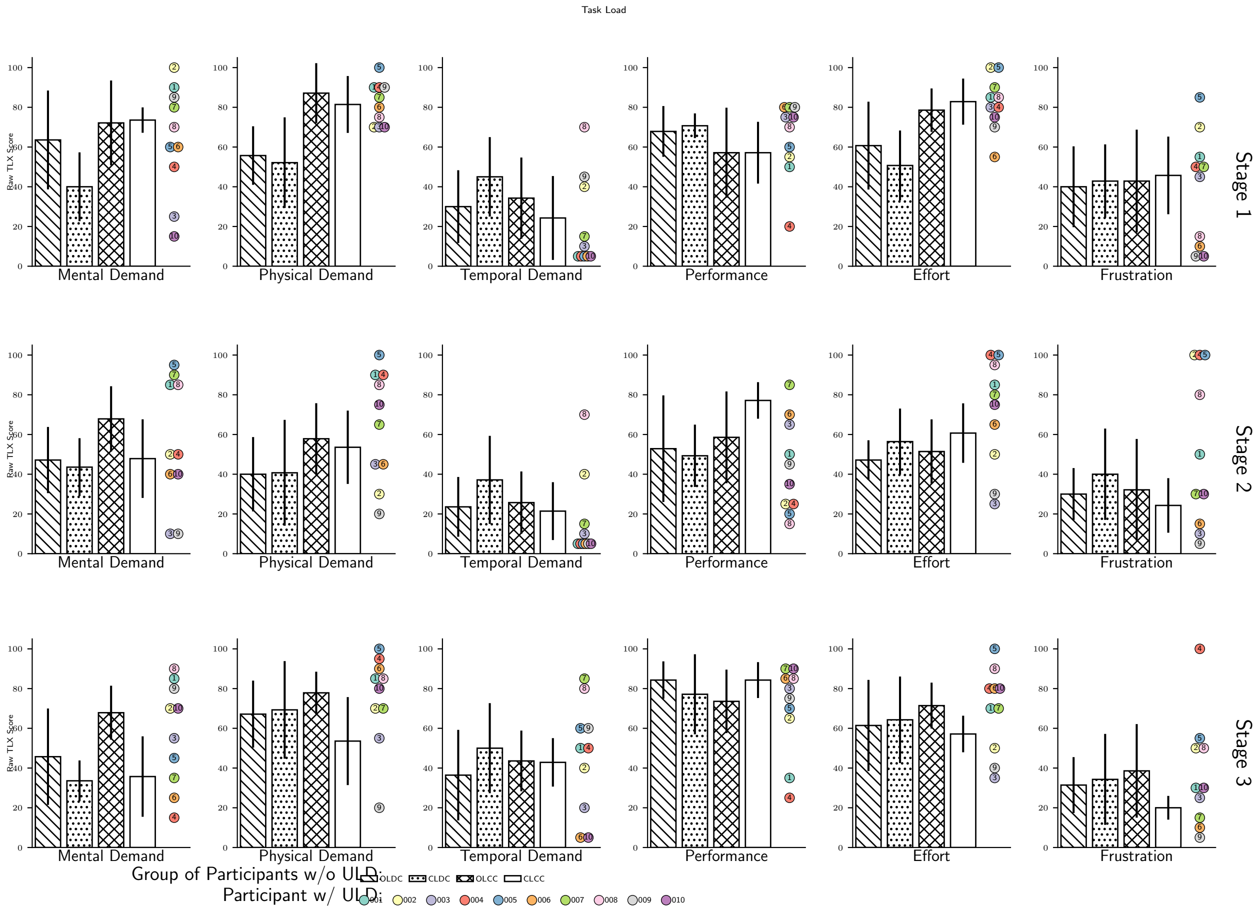

Figure S21. Task load results of groups of participants without limb difference and individual participants with limb difference. Raw NASA-TLX scores of groups of participants without limb difference and individual participants with limb difference, taken at three stages during experimentation. Stage 1: after position matching experiments, Stage 2: after force matching and object detection experiments, Stage 3: after clinical dexterity tests. Results of paired t-tests are shown at $*p < 0.05$, $**p < 0.01$, and $***p < 0.001$ levels.

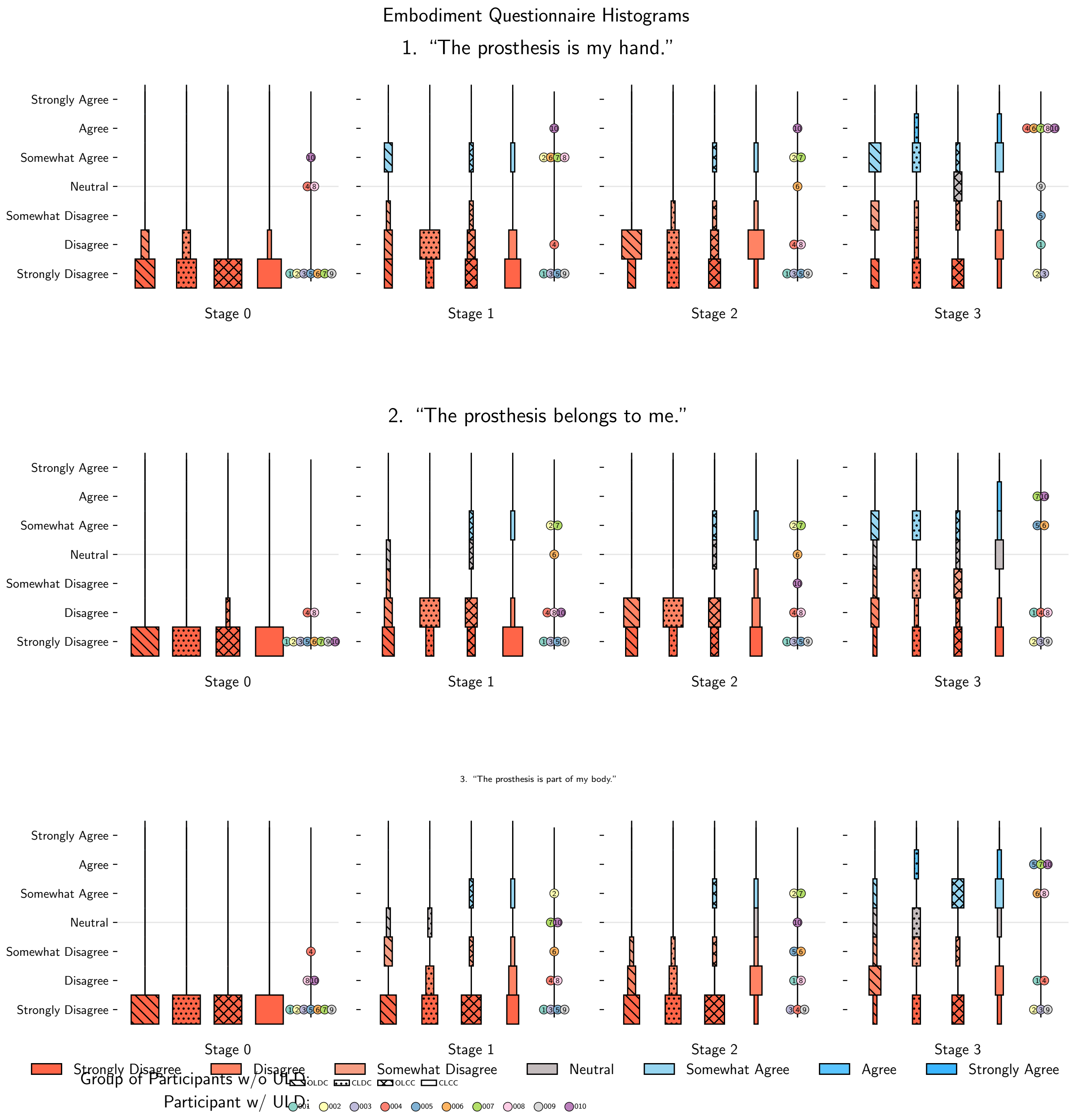

Figure S22. Prosthetic embodiment results of groups of participants without limb difference and individual participants with limb difference. Responses of groups of participants without limb difference and individual participants with limb difference to prosthetic embodiment questionnaire statements. Results of paired Mann-Whitney U tests are shown at $*p < 0.05$, $**p < 0.01$, and $***p < 0.001$ levels.

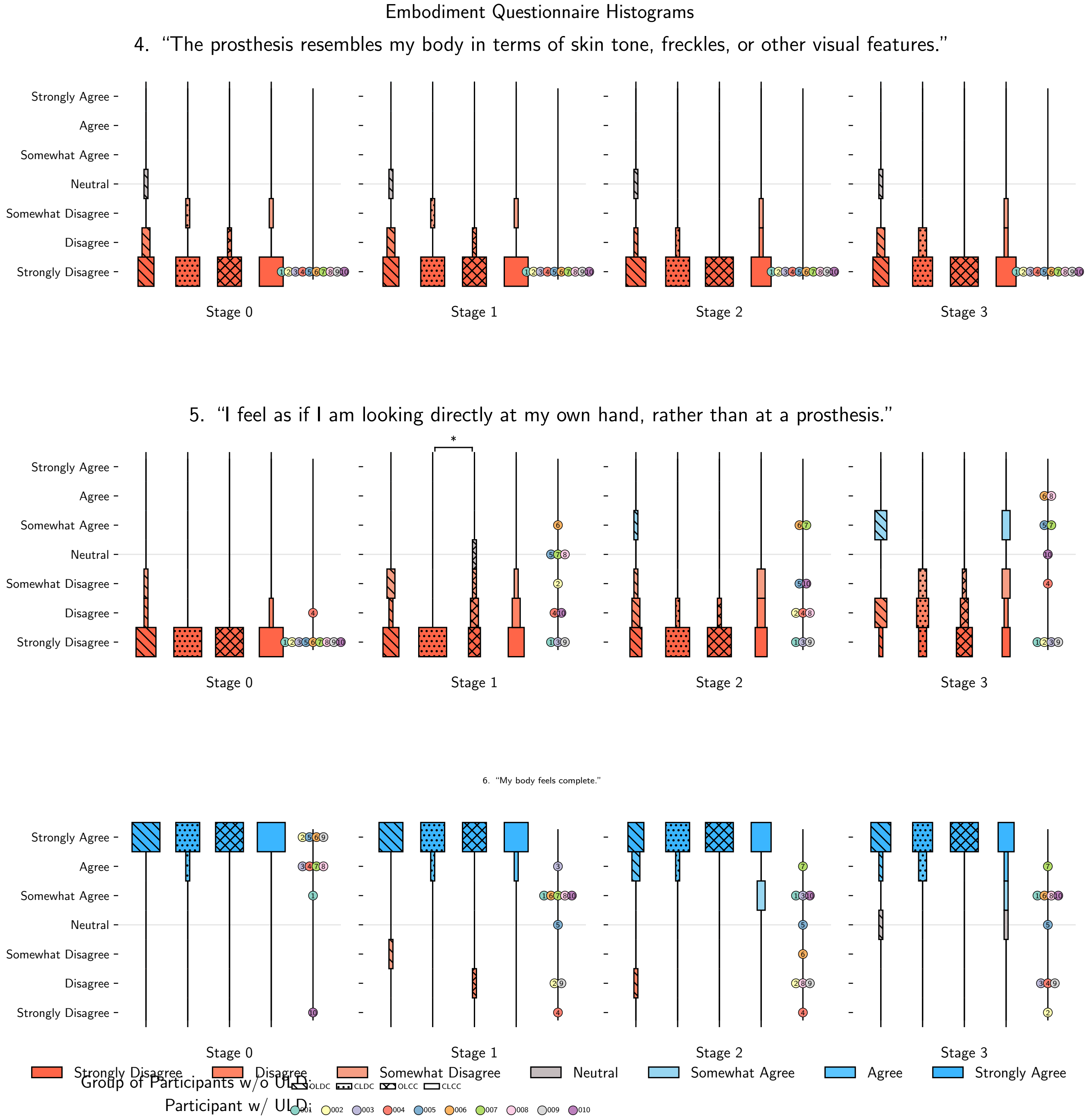

Figure S22. (Continued) Prosthetic embodiment results of groups of participants without limb difference and individual participants with limb difference. Responses of groups of participants without limb difference and individual participants with limb difference to prosthetic embodiment questionnaire statements. Results of paired Mann-Whitney U tests are shown at $*p < 0.05$, $**p < 0.01$, and $***p < 0.001$ levels.

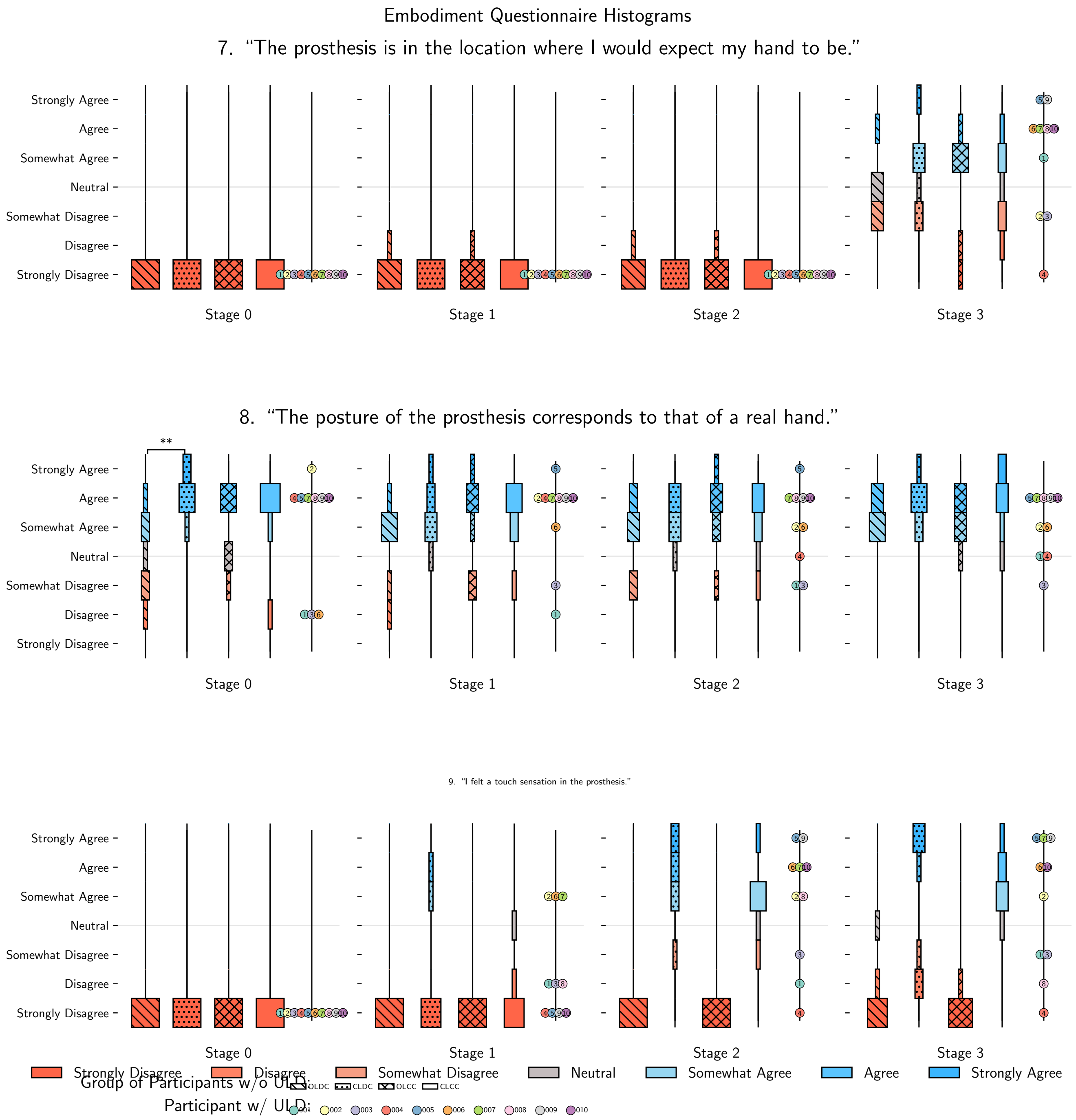

Figure S22. (Continued) Prosthetic embodiment results of groups of participants without limb difference and individual participants with limb difference. Responses of groups of participants without limb difference and individual participants with limb difference to prosthetic embodiment questionnaire statements. Results of paired Mann-Whitney U tests are shown at $*p < 0.05$, $**p < 0.01$, and $***p < 0.001$ levels.

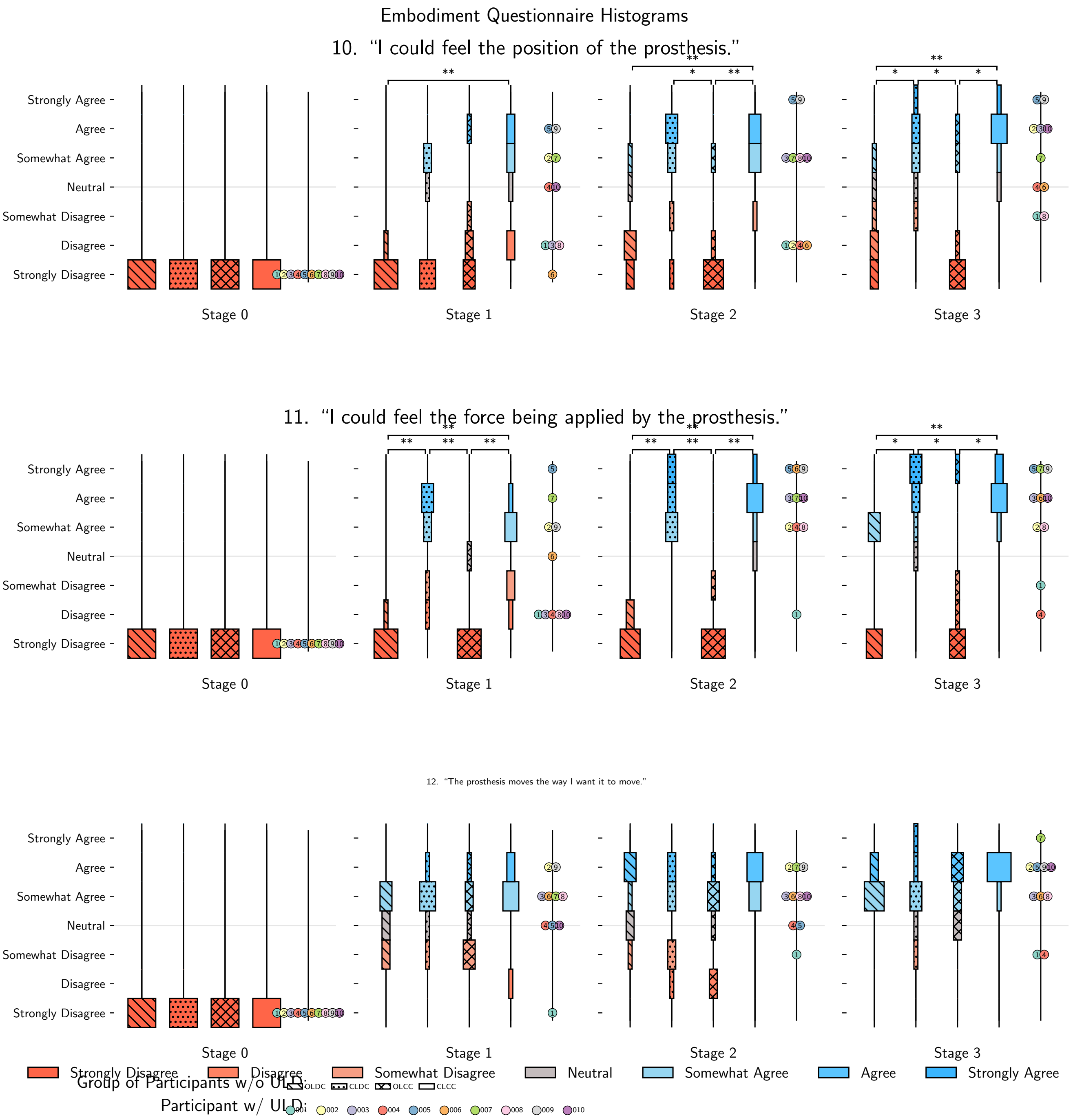

Figure S22. (Continued) Prosthetic embodiment results of groups of participants without limb difference and individual participants with limb difference. Responses of groups of participants without limb difference and individual participants with limb difference to prosthetic embodiment questionnaire statements. Results of paired Mann-Whitney U tests are shown at $*p < 0.05$, $**p < 0.01$, and $***p < 0.001$ levels.

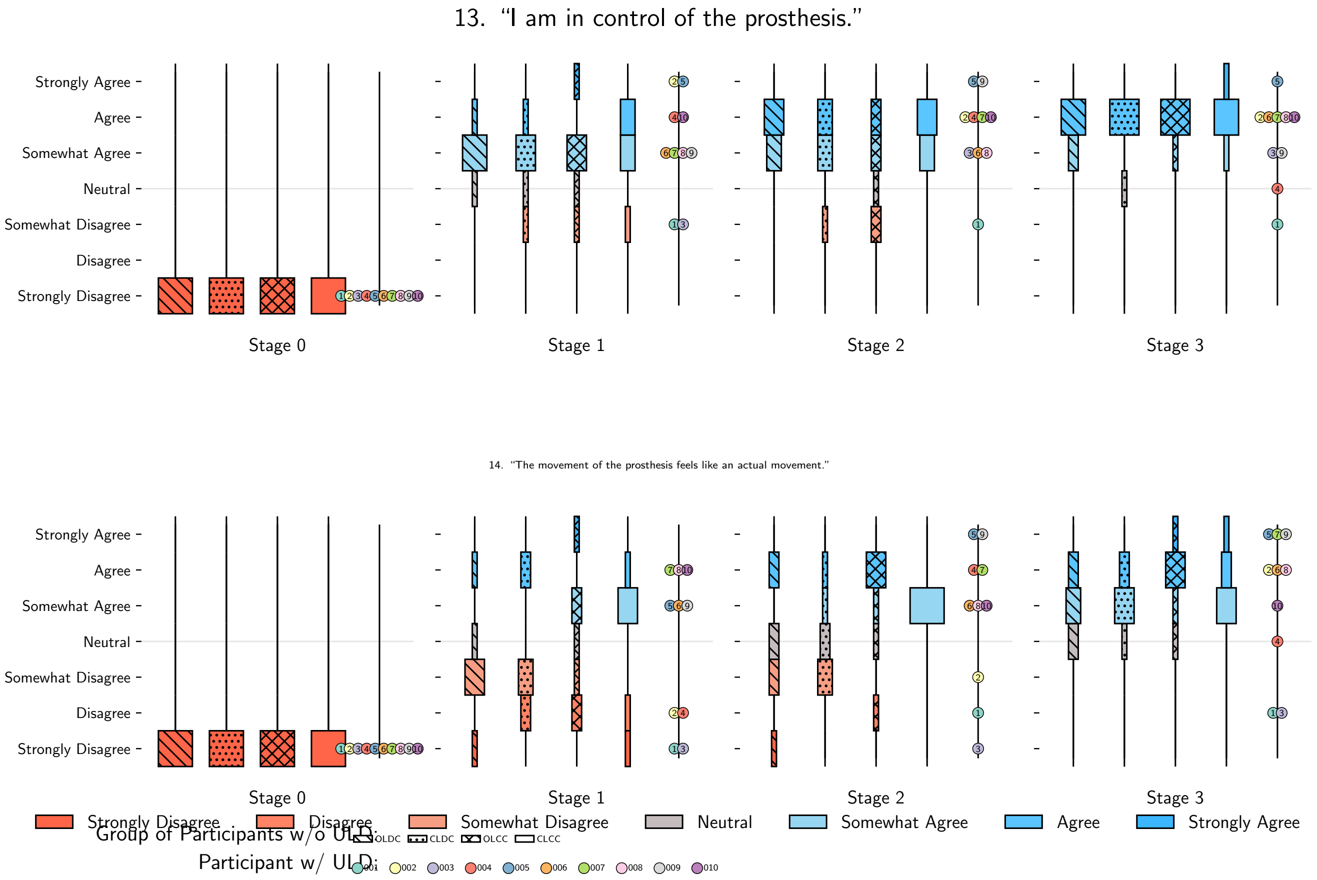

Figure S22. (Continued) Prosthetic embodiment results of groups of participants without limb difference and individual participants with limb difference. Responses of groups of participants without limb difference and individual participants with limb difference to prosthetic embodiment questionnaire statements. Results of paired Mann-Whitney U tests are shown at $*p < 0.05$, $**p < 0.01$, and $***p < 0.001$ levels.

Supplementary Tables

Table S1. List of Abbreviations

Participant Groups	
CLCC	Closed-Loop Continuous Control
CLDC	Closed-Loop Discrete Control
OLCC	Open-Loop Continuous Control
OLDC	Open-Loop Discrete Control
Upper Limb Difference Identifiers	
LAAE	Left Arm, Above Elbow
LABE	Left Arm, Below Elbow
RAAE	Right Arm, Above Elbow
RABE	Right Arm, Below Elbow
Causes of Upper Limb Difference	
C	Congenital
PS	Planned Surgery
TI	Traumatic Injury
Other Abbreviations	
BBT	Box and Blocks Test
DOF(s)	Degree(s) of Freedom
EMG	Electromyography
JTHFT	Jebsen-Taylor Hand Function Test
KDE	Kernel Density Estimation
MAE	Mean Absolute Error
TLX	Task Load Index
ULD	Upper Limb Difference

ID	Age	Gender	Affected Side	ULD Level	Cause	Years Since	Dominant Hand	Own Prosthesis	Frequency of Use	Session Format
001	26	Male	Right	Below Elbow*§	C (TI)	26 (8)	Right	Cosmetic	Infrequent	Single
002	54	Male	Left	Below Elbow	PS	5	Right	None	-	Single
003	30	Male	Left	Below Elbow	C	30	Right	None	-	Single
004	42	Male	Both (Right)	Below Elbow	TI	9	Left	Body-Powered	Daily	Multiple
005	31	Male	Right	Below Elbow†	C	31	Right	Static	Infrequent	Single
006	30	Male	Right	Below Elbow	C	30	Right	None	-	Single
007	30	Male	Left	Below Elbow	C	28	Right	Static	Frequent	Multiple
008	45	Female	Right	Below Elbow‡	TI (PS)	24 (6)	Left	Static	Infrequent	Single
009	22	Male	Left	Below Elbow†	C	22	Right	Static	Frequent	Single
010	62	Male	Left	Below Elbow§	TI	3	Right	Static	Infrequent	Single

Table S2. Table of participant characteristics of individual participants with limb difference. For bilateral upper limb differences the side tested is shown in brackets, and details for the tested side are shown. Causes listed: congenital (C), traumatic injury (TI), planned surgery (PS). *Participant 002 acquired a higher level of ULD than their congenital level from further traumatic injury; the muscles of the upper arm were used as an alternative input. †Participants 005 and 009 were only able to activate two muscles in their residual limb. ‡Participant 008 acquired their ULD via planned surgery after a prior traumatic injury, and received targeted muscle reinnervation; the muscles of the upper arm were used. §Participants 001 and 010 had no functioning elbow joint.

	OLDC	CLDC	OLCC	CLCC	All
Age/years	26.1 (5.7)	24.4 (2.5)	23.6 (6.4)	22.7 (3.1)	24.2 (4.6)
Male	6	6	5	6	23
Female	1	1	1	1	4
Right Handed	6	5	7	7	25
Left Handed	1	2	0	0	3

Table S3. Characteristics of groups of participants without limb difference. Age, gender, and handedness of groups of participants without limb difference. For age, standard deviation is shown in brackets.

Item	Question	Adapted From
1	"The prosthesis is my hand"	49
2	"The prosthesis belongs to me"	49
3	"The prosthesis is part of my body"	49
4	"The prosthesis resembles my body in terms of skin tone, freckles, or other visual features"	49
5	"I feel as if I am looking directly at my own hand, rather than at a prosthesis"	49
6	"My body feels complete"	49
7	"The prosthesis is in the location where I would expect my hand to be"	49
8	"The posture of the prosthesis corresponds to that of a real hand"	49
9	"I felt a touch sensation in the prosthesis"	49
10	"I could feel the position of the prosthesis"	—
11	"I could feel the force being applied by the prosthesis"	—
12	"The prosthesis moves the way I want it to move"	49
13	"I am in control of the prosthesis"	49
14	"The movement of the prosthesis feels like an actual movement"	49

Table S4. Prosthesis Embodiment Questionnaire for Upper Limb Prostheses. The questionnaire used to assess prosthesis embodiment for upper limb prostheses. Each question is answered using a 7-point Likert scale.

Post-Study Interview Transcripts

In all transcripts, the researcher conducting the interview is denoted 'R', and the participant is identified by their participant ID (e.g. participant 005 is identified as 005).

S1 Participant 001

R: I asked you three statements at the start, which were "the prosthesis is my hand", it "belongs to you" and it's "part of your body". So we asked you at the very beginning and he said strongly disagree – that's expected. You hadn't started using it and then after stage 1 when you'd used it a bit, you still said strongly disagree. So on stage 2, so after we put the blindfold on you and you had to go and identifying objects and also squeezing onto the can with a target force, you said, we asked you about the prosthesis [being] part of your body, that rose slightly. So that went from a strong disagree to just disagree. I'd like to ask a little bit more about what might have caused that or what you remember from it.

001: OK, I can distinctly remember this point. The way I'd articulate it was I felt after that my brain was starting to work it out. And in a very rudimentary sense, so how the system it had been placed in to operate the arm worked like the 'rules of the game', so to speak. And it was although it was never going to feel natural or ergonomic because of the constraints placed by the actual [prosthesis] – the fact it wasn't fitting correctly to the handle wasn't fitted at all [on the desk] in [stage 1] and stuff like that. And because it was inherently a bit uncomfortable. My body was starting to work out what movements you needed to do to achieve certain goals, and that for me was a big piece of the puzzle. It's like almost 1 foot forward about how this system [worked]. My brain no longer saw it as a difficult task that couldn't be defeated. It was something that I could learn to develop my skills on. There was an avenue to develop that sort of ability and that sort of led in to start that feeling. And about the hand becoming a part of me in the same way that my prosthetics that I use at home now. They, as I developed their roles and their uses and how to use them, the more they feel a part of me. It's not necessarily the fact that they're made of plastic or anything like that. I think for me at least, I don't necessarily see that it's their usefulness and that was a small part of developing that usefulness. So I could see there could be a use for it, if that makes sense.

R: What it is about a hand being useful that relates it to being part of your body. What do you think it is about that usefulness?

001: In my opinion, it's a tool – and this might be my outlook in life because I have never had a hand on my right arm. I maybe assign something different, maybe assign a different feeling than I do with [my left] hand. [My left] hand is a part of me. But whereas I forever see anything [on my right] as something that is just an extension; artificial. So I assign value to being a part of me is how useful that tool is at the end. If that makes sense, it's probably the best way I can articulate. It translates to a feeling of being mine in a way.

R: That that's very interesting, thank you. So [after stage 2], it started to feel like you were working out how this is gonna work. But, on the other two questions, "the prosthesis is my hand" and it "belongs to me" [your response] stayed at strongly disagree. But then after stage 3, when you're wearing the hand, they increased to just disagree instead of strongly disagree.

001: Yeah, I remember feeling when it was on the table, it wasn't on me. I could start to understand the, like I said, the rules of how this worked. Then when it was fixed on me, although it was uncomfortable, it started to feel more like, you know, the best way to describe it is like 'we could do something with this'. We could get a functioning hand out of that if we made those certain changes and that sort of really helped to increase that feeling of like, "yeah, it was a part of me". You know, I could see myself wearing this day-to-day. With the whole feeling and the whole, the whole kit. If it was made more ergonomic I could see that system becoming useful for me. It was the closest thing I've ever had to a feeling of a proper hand being able to work like a proper hand. I've never had that before, I've only ever had hooks or claws. And I know, although the limitations of the hand are fairly limiting, it was nice to get that feeling of being able to, for want of a better term, think and have it happen.

R: So your point of reference for that was having previously tried with hooks and claws that don't look like hands. I'd like to understand a bit more about how the look of the hand affects your sense of it being part of your belonging to you.

001: It's tricky. If you'd asked [me] a couple of years ago, I probably wouldn't have assigned any value to that because I was always very… my arm was a hook back then. Nowadays, especially since I've sort of – this is a bit more history than probably warranted in this interview – gained more of a feeling towards having something that looks like a hand on my [affected] side. So I think yeah, the look of it, I mean aside from the colour obviously, but the like the look of it is important as part of me. I think that's right in terms of like it 'felt' like a hand that had purpose, that had use, and I've not really had that before, mainly because my the hands that look like hands are just cosmetic. They're designed to look like a hand but don't serve any purpose. The only thing I've ever found real use out of was my hook, which obviously looks nothing like [a hand]. I would say that [the hand-like appearance] definitely aided in the feeling towards it being a part of

me. It wasn't the main part – that's the use – but the look of it, I could see it. I could see wearing it to work, you know having that one arm instead of having to chop and change [between cosmetic and hook prostheses].

R: The hand you mentioned that looks like a hand – was that a static hand?

001: Yeah, I've had a fair number of limbs. I've had primarily cosmetic prosthetic hands that look very similar to normal hands. I've had one [myoelectric] hand, which is a hand, but it's just a claw like that [gestures a claw grasping motion]. So that was very short lived. It was very heavy. It was very cumbersome. And then I've had mainly hooks which open like that [gestures a split-hook action]. I could see myself gaining the most [out of a hand] with the use that I get out of the hooks but an entity that looks like a hand, so I'd be able to just operate normally with it. I think as I've come a bit older, looks have become more important to me and in certain ways, but not it's not vanity or worried about looking about what how other people look, it's just more for... You know, it's just, I don't know. I don't really know what it is. It's just something I feel more comfortable with. Just looking a bit more normal.

R: So there's this interesting relationship where your priority is that it has a purpose, and then it's extra nice if it also looks how you like it to look?

001: Yeah. And I think that for me will always be right, because I've always seen anything beyond my [residual limb] as a tool. It's serving a purpose, be it making my life easier bit, its ability to take up less concentration in order to achieve its objectives in the same way. You don't need to apply all your concentration to pick something up, as you know where it is. And I think that's the way, especially when we were doing the hand stuff to work out what bits were, I started to feel like my brain could learn this. It could pick it up and it would be able to offload some of the more concentration focused tasks around like how or what object I'm picking up or something like that. That's, that's why. And then on a secondary point, yes, you're right, the look of it.

R: The next statement I'll ask about is "the posture of the prosthesis corresponds to that of a real hand". Initially you said disagree, and then after stage 1, you also said disagree. After matching positions after stage 2, which is the blindfolded one and the grabbing hold of the can it went up to somewhat disagree, and then after wearing the prosthetic hand and doing tasks with it [in stage 3], you said neutral.

001: Yep, seeing how it moved in terms of just the basic tasks and helped develop that. I think wearing it was the real thing and I and I think again this probably plays into the fact that I just had five fingers working to achieve a grip and not only that, but I had five fingers working with a degree of information coming back from said fingers. I've never had that before, especially from a hand like structure. So that's probably why the rating went up there in terms of you look at it to begin with, it's not attached to your body. It moves in quite a like an automated way. And then the more I picked it up, the smoother it moved, the more I could get it to do what I wanted it to do and that sort of fed back into the fact that this can move like a hand if I can learn how to do it. Plus, the information coming back to me around the sort of grip strength on them was useful. It gives you more information than, say, a blank prosthesis would, and I think that's probably why that went up in terms of the look and the feel of the arm.

R: There was this question, "the prosthesis moves the way you want it to move". After the first, so at the beginning, it was strongly disagree because you haven't done anything yet. So after position matching [stage 1], also strongly disagree, and then after stage 2 with the blindfold and stage 3, they were both somewhat disagree. It might be asking you to repeat some of things you already said, but why do you think like this?

001: Like I mentioned earlier, it was the feeling of getting the hang of it. I'm being honest, it probably plateaued because my ability to concentrate on getting the hang of it, my energy level, came down below the line of actually being able to learn, so they probably passed each other probably midway through the [study] and my sort of ability to learn was starting to wane, but risen initially because I felt that there was a possibility for progress here. It wasn't an insurmountable task. So that's why that feeling developed [which] is like "I can get it to do what I want". I just need to focus on it and that sort of stuff. And I definitely felt that if I continue to do it maybe with a fresh head, I would have continued to get better at it.

R: Understood. So like a longer term over, say, a couple of days or a couple of weeks?

001: Yeah, with a more ergonomic design, definitely. I feel like I'm bashing on the design. I do know it's a prototype.

R: No, please don't apologise. I need to ask more about that ergonomic design. What do you mean by this?

001: I mean in a perfect world, I'd like one tailor fitted to the to my little arm. I know that's not feasible, but. The sensor band wasn't too bad. I think that was by far the best part of it, and I understand that's more me and the constraints of what we had to work with in terms of muscles and where it was best fitting. I think as well the blocky force feedback [armband]. Not only was it uncomfortable, but it was very it was just it was big and numb. I think if that was obviously a lot smaller, I understand the difficulty in building these sort of things, but if that was a lot smaller and a lot lighter and

less lag generally as well. If those changes could be made, I think you'd be starting to see a fair few higher scores on those. Does it feel like your arm, can you get to do what you can do? I said it would be easier, especially with the lag, it would be easy for the brain to pick up what was happening.

R: It's good to have this noted, down from someone who's used it as opposed to just [a researcher's] view of it. I'm going to ask you about your perceived performance in each of the tasks. For position matching you had that down as 50 out of 100. For the second one, which was the blindfold one, you also put that down as 50 out of 100, so. For the final one, where you wore the arm and you did the task it was at 35. So I'd like to know your thoughts on that.

001: So 50 out of 100 for the first, and second. I remember the position matching one. I was really sort of happy with the fact that I could get it to [move] there. I felt after we've done a bit of that bit of that position matching, I felt, like I've said, I understood how the thing worked in terms, but it was very rudimentary. And then the blindfolding one, was the first time my brain had to literally like literally interpret the force feedback and I didn't really understand it enough to comprehend to fully sort of feel confident and be able to do that. I didn't think the movement was a problem. Apart from the sort of the actual mechanics of it that we were having difficulty with, like you know, slipping off the arm and stuff like that, but it was more the ability to understand what I was feeling. I hadn't had enough time with it to… it was very, very broad strokes in terms of the force we, but I still felt that I could still understand and get it to do what I wanted to do. I think towards the end where I was where it was mounted to me that it was limited by the mounting of it, and the I think tiredness when it comes to executing what I was trying to achieve basically. The core components are there in terms of like what [the controller was] trying to, what you were trying to achieve with that arm. It's just the design and the ergonomics and spreading out the learning with it. You'd get there. I feel definitely the actual the brain can do the rest. I felt if you take anything away, I felt that I could get there with enough time and enough perseverance with it. I felt that the force feedback and the information was there, I just needed time for my brain to learn and interpret it, and the energy too. I mean, that was something I could have done a bit better with in terms of taking a few more breaks instead of just pressing through it.

R: I'm gonna ask you about frustration while using the prosthetic hand. So, for stage 1 so the position matching you said 55, after stage 2 you said 50, so that was after the blindfold and the objects, and then after stage 3 it went down to 30. What do you reckon was going on?

001: Frustration feeds back in for me personally; I would have gotten more and more frustrated if I felt I couldn't learn a system. But because that wasn't the case, because I felt like I was making some progress, the frustration went down. Especially towards the end when I had that success with the [stacking checkers] and the moving the objects and stuff like that, I felt like I could get this to do what I wanted to do. I was starting to understand and comprehend how it could work. And not only that, but how it could be useful to me. And that that's why that frustration level was going down. It started off slow, I think. Over the middle period I felt a bit frustrated by the fact that I was trying to understand the force feedback and pick up what it was trying to tell me and interpret it, which is probably why you don't see a [decrease in frustration].

R: The nature of the third stage is that you have these tasks in front of you and it's quite tangible and you can see how you're doing on the task.

001: Yeah, and that probably did play a role in it. Having an objective and achieving that objective using the arm feels good. With not as much expected difficulty. I thought [I was going to] struggle a lot on some of those tasks and it wasn't as hard as I thought it was gonna be. I don't know whether that was necessarily, uh me having spent more time with it up to that point, whether it was because, you know, the force feedback or whether it was just because I just managed to get it in a good position when I closed it. It's probably a mixture of all three. But yeah, generally it's being able to see a task and complete it without as much difficulty as one would initially expect. I would still find it easier to achieve those tasks with my hook than the arm, but obviously that's because the hook fits me a lot better. It's a lot lighter. Although it doesn't give me as much feedback in terms of the [cable], I'm much more akin to what the tension and the wire that would connect my shoulder to the actual hook. I've got more [learned] information around how that feels and what it means. And I think that could all happen with enough time. With the arm, the only difference is time. I've spent years using a hook, so I've spent years to fine tune in the same way you spent your entire life understanding what your hand is telling you. For me, my body just interprets the tension in the wire to how open they will close the hook is, and the same could be said about the position of the pads on your upper arm. In terms of the force feedback, the information's there. It's just the time it takes to develop that understanding of it, and I think the key for that frustration is my brain could see that path of understanding it. It could make sense of it, "I'm not there yet, but I can work on this". If you gave me enough time and enough exposure with it. It was probably aided in the fact that I went in thinking it was gonna be really difficult – and it was – but it wasn't anywhere near as difficult to pick up as I thought it would be.

R: I would like to ask is there anything that we haven't talked about yet that you'd like to bring up?

001: Not really. I think it's the implementation for me at least. It was a challenge in terms of the discomfort [the haptic feedback armband] was causing on the skin [of the upper arm]. There were no, like lasting effects of it. It was just mild discomfort at the time as it was rubbing against the scar point [on the upper arm]. Also having a proper stand so that the person doesn't have to load bear the whole [prosthesis]. I know we have the strap which worked really well that took a lot of the weight off my arm and allowed me to focus on the task was very useful definitely.

S2 Participant 002

R: There was this question which was the "prosthesis is my hand". After we got you to match the positions [and forces] and then also identify objects with the blindfold on. You said, you somewhat agree, but then after you wore it and did the pick and place tasks you said you strongly disagree. So, I want to understand a little bit more about that and get your thoughts on it.

002: Right, visually what I find is with a lot of prostheses they don't actually give you a hand that looks like a hand. As a replica, if you took away the skin, I would say [the prosthesis used in the study] would resemble [a real hand]. The fingers of a normal, everyday functioning hand so visually there you can [imagine] that as being part of your hand. Right, but when using it you realise how disconnected you are from it. You quick you very, very quickly realise that. It doesn't move the way that you want it, it feels like an appendage on [the end of] what you are used to now. And in my personal opinion, that comes before the actual using of the [prosthesis]. You have to very strongly think of how to use the [prosthesis].

R: I'm really interested in the bit where you said where you put it on, and it becomes like an appendage.

002: I personally, and mentally, cut my hand off my arm when I knew it was getting cut off. It was dead to me. I quickly got very used to the idea that my arm was no longer there. I'm lucky enough never to have received or had phantom pains that I am aware of. Now if I have, I've not took them as phantom pains; I have took them as just everyday living with my condition or my situation. You get used to having nothing there, so when you put something on your arm where you've been cut or amputated to. If you actually think that that there is your hand, even though you can't open it and stuff like that, but when you put something onto that, it's like you grabbing hold of the stay of the brush and holding it out and you can feel that your arm wants to go down. If you went into your kitchen and there was a long sweeping brush and you picked it up and you held it by the handle and lifted it. It would naturally [go down] because gravity pushes it down. So that is what a prosthetic arm feels like. So do you remember when I said to you right at the beginning [of the study] that you will never get a prosthesis working correctly, because the first thing that you need to do is make sure that that prosthesis feels a part of that person.

R: So would you say that the way that it feels like it's part of you is a lot about how it's sort of connected or attached to your arm?

002: Yes, and I also think that the sooner that you can get an amputee a prosthesis the better. But, I also think that surgeons need to understand [that], when removing a limb, where is the best place for that person? Like think not in that moment but think a week [ahead]. When this person does have a prosthesis, if I cut it off here, is it going to help or is it going to more hinder that person with the prosthesis? So if I'm able to cut there, that would give them a better chance of using a prosthesis, which would give them a better quality [of use]. You get what I mean?

R: I remember at the time you said that there was something about the way it felt with the socket and it wasn't quite rigid.

002: Yeah, you mean the one that you've got?

R: Yeah, the canvas or fabric socket.

002: Yes because it was strapped to me round once. I mean that [socket] is the test piece so it would be very difficult for you to literally it or physically make the [socket] per person, for everybody.

R: What I would like to understand is that when it was mounted on the desk, and it wasn't attached to you, you said that you somewhat agree that it is "part of your body" and that it "belongs to you".

002: Basically, I think it's because being an amputee you hold hope. I think that's the only word you could use really is "hope" that the prosthetic limb that is there, designed for you, will work. Because I know now being nearly six years down the line [since amputation] that it is very difficult for people to make a prosthesis as an upper limb, because of the complexity; the movement of the fingers and the hands. Now, until – from my personal point of view – these little motors become a lot stronger, then you're limited to what you can use that prosthesis for. So when you look at [the prosthesis used in the study] that you made. A blind man touching that would be able to say that is a hand and they will be able to know that then fingers physically bend and move. Whereas the old prostheses, they made them to look like there was a proper hand visually; from a distance they look like a hand, but a hand that doesn't move. Whereas [the prosthesis used in the study], you can clearly see that the fingers do move independently – and that's it. So that's why, when it's on the table, you can see it's a hand and you can relate to that as being your hand – not that that is your hand – but you can relate to that with the hope that it will work and you will gel with it.

R: So when it's on the table a lot of this is how it how it moves and how it how it looks?

002: Yeah.

R: Until you actually wear it and then something changes?

002: Yeah. It's like there's like a line that you can't go across, because there's no connection with [it]. Now, I'm assuming, or I'm thinking, that if something, if the sensors were better and they were more adapted to the physical movement of your arm when using the prosthesis, then you will probably feel more in tune with that. There's lots of different elements. [There are] far more elements to a person being able to use a prosthesis as a robotic arm. Whereas a lower limb, you are connected with it straight away. Even though it feels funny at first because it's just there and it does only two things, it bends at the knees, bends at the ankle. Or maybe three things: it keeps you upright [as well]. If your balance is fine, it doesn't get asked for anything more. When [more] things are asked for you then have the blades and other implements that are specially designed. There isn't that yet with an upper limb. The strapping [of an upper limb socket] to you restricts your movement. It also, unless you've got the correct or a decent, like with me, [longer residual] limb, lower below elbow then you can't. There isn't the structure there to [give] stability. So, because the stability is not there and a lot of the weight is on the end, it will naturally gravitate to dropping down. See, I do think even though I've never actually looked into one of these "robotic ones" that they have. It might be worth being able to see how they have the sensors because [those hands] seem to be [performing] quite well. My experience other than saying "******* *****" is that they do the bare minimum for people [in the UK health system] and it's absolutely wrong. So, I mean this is a [comparison] scenario, a lady that I knew that had her leg amputated at the same time I had my arm. The three-year period she had. Extreme physiotherapy She had [multiple prosthetic] legs. She was then given this thing called a C-leg, which costs money. I roughly estimated, conservatively, probably about £100,000 [including] physiotherapy, legs, and the C-leg. I was told that the Ottobock Griefer arm was the latest technology. Now I never gelled with it because it when I moved my arm, it sent different signals, so I couldn't control the signal by using my arm. Plus there is quite a bit of weight on the end where your hand is because you haven't got [a limb] from your elbow to the wrist, that structural strength [is gone]. So I was watching this [movie] set in the 70s. And they go into this hospital where they show this robotic arm and there it is the Griefer. It's literally there, so it's a 1970s product still being used in the 21st century. 50 years down the line. We were told by the people at [the hospital] that there isn't as much money into upper limb because people do not use them like they do the leg. Well, that's a catch 22. If there was more investment, it would be better. These people that are using the Hero arms, they're using them. They must be getting made out of some something that is making them light weight. It could be the fact that my arm, because I've only got like 4 inches below the elbow. If I had 5 inches maybe? That would help. Or 6 inches. I don't know, but this is why I'm saying. I think surgeons need to be [thinking about] afterwards when people do come to need these prostheses. So for them to know that if you cut it rather than there do it at above the elbow then that helps.

R: You were mentioning a lot about rehabilitation [for] people who've lost their leg. How do you feel about rehabilitation for people who've lost an arm?

002: Right, my experience from even when I had the cancer. I was just left to fend for myself and my arm was amputated. I was told I was part of this family now and I could phone [the rehabilitation center], contact [them] anytime. Very shortly after it was made clear that myself and other people, if we had problems, we had to go to see our doctor not [them]. I went to [the rehabilitation center] the attitude of the consultant that I saw... [he] was an old consultant. And he was very, very old fashioned. I'm very liberal and I say a lot of things, but I was very surprised by a couple of the comments and the actual process that the consultant came out with then. When I actually went to see the guy who was making the prostheses, he was part time because he was retiring and it didn't feel that my needs were being listened to. It was "this is what you get – deal with it", which frustrated me a lot more because it felt like [that] every time I went to [the center]. I then asked the hospital in [a second location] to see if they could contact [the second rehabilitation center] for them to take me over because of the way [the rehabilitation center] was being. I don't think they were being naturally rude or anything, but I expected a lot better than what I was getting because I think that being an upper limb [amputee], I needed [better] and I didn't feel that I was getting it. I think basically the best way of describing my very first [prosthesis] was. It was that big and funnily shaped that if a coach load of people lads was going down the motorway you could have quite easily and put it on the side of the motorway and used it as a portable urinal. That's how it felt and looked to me, and once I thought about, I couldn't get that out of my mind. Because it just wasn't... I was disconnected with it. There was no connection between that prosthesis and me in any way, shape, or form. Then being in [the second rehabilitation center], there was a young guy and he was very good and he was full of hope. I asked after a while for these robotic arms because I was in England and I lived in Wales. I had to then go back to [the rehabilitation center], because that was my country that I lived in so I couldn't really cross the border. As much as they helped me as much as they could, after a bit, when I wanted actual big money [for a more sophisticated prosthesis], [then I had to be] dealt with through [the rehabilitation center]. When I did go back [to the rehabilitation center] they were quite apologetic, but I was full of hope. I was full of hope, and what was said to my face [reinforced this feeling]. Soon as I went out the door, it felt like, "well, he's not

getting that, he'll get what he's given". As much as I tried to work with [the rehabilitation center], I was taking time out of work to go there, so I was losing money. It was costing me money and the actual arm was just no... it wasn't any good.

R: Can I ask you about another one of the other questions I asked you [during the study]? The statement was "my body feels complete".

002: I'm happy with the way that I look and I'm happy with not having my [hand]. Yeah, that it doesn't affect me.

R: So, before we started you said strongly agree. Then what we found was that when we started the experiments, your response went down to disagree. By the end [of the study] it was strongly disagree.

002: Obviously when you're doing these trials, or you're dealing with prostheses it comes very quickly back to you that you have lost an appendage. You quickly then revert back to knowing that you've not got that hand. I don't ever think twice about doing anything with my arm because I'm so used to it not being there now. My end of my stump, I class that as my "grip", if you know what I mean? So I use that for carrying things and stuff like that. When you come to do [tasks] with a prosthetic arm you quickly realise that it's not there. It quickly comes back that you do have this [limb difference]. But I do find that, in my case, prosthetic arms made me more disabled, because they do not benefit me. I've never had anything that [does benefit me] because the ones that I have had have never gelled or helped me. Going back to the same thing; to me to make sure that these hands will [gel], it has to feel natural to you. And is it? If it doesn't feel part of me, it's always going to feel strange. I've never experienced a [multi-articulating hand]. I did go looking about for them because there is a place nearby and they do robotic arms there. They said to me that "you're looking at thousands and thousands and thousands of pounds for them". If it knew it would work and it would enhance my life. I'm not asking for it to do a lot. I'm asking for it to be able to grab a can of coke and be able to have a drink. Or the best way I used to put it is to give my right arm a bit of respite. Because my right arm is constantly doing everything. If I had [a prosthesis] and I could rely on it on sometimes then [my right arm] would get respite.

R: The amount of frustration you experienced, after the blindfolded stage, you said your frustration was 100%.

002: Yeah, because you've got no feeling. You can't mentally visualise what you've got in your hand or across. There's no connection; you could have put a leaf in that hand. Or you could have put a screwdriver in it. I would know what it was until I visually saw it.

R: I need to poke a little bit more at this one to understand. What is it about not having this connection that made it frustrating?

002: There's no feeling. Like I said, I could have a screwdriver in my hand. I wouldn't know. With my right hand, I have sensitivity so I can tell, or I can relate with seeing something and the touch... it relates. It stimulates your brain to know what it is. Because there's no touch, or no stimulation, my brain is just blank. I mean, it's like now I can touch something with my right arm and my left arm, and I know I'm touching something. I know if it's hot, I know if it's cold. If I had my eyes closed, I could hazard a guess, because if it was a ball, I could feel around it if it was some kind of shape, I would be able to tell you possibly what the shape was not as well as my hand. Because of the dexterity [in my hand] and the amount of nerves that's in there to let, you feel what you're touching. With the [residual limb], I'll get some sensation, but not a lot. A prosthetic arm, the way that they are at the moment, you have to visually see what you're touching to know what it is. I don't think you will really ever gain the stimulation with a robotic arm. Unless it's permanently [integrated with] your nerves. So to me, the only way that things like that would be able to work is... with similar things that you're doing with the vibration [feedback]. I don't think you'll ever get the actual feeling of the proper arm because [it's too complex] and stuff and you couldn't put my [hand] in [the prosthesis].

R: One final question: is there anything that we haven't talked about yet that you would like to talk about?

002: One of the big things I think is that the little motors that you require to move the fingers independently [based on] your muscle movement or your sensors. They need to be lightweight as possible, with as much power as possible, because if they haven't got the power then you can't grip a can of coke. So if you can't grip the kind of coke to drink, then it's all in that motor. I think what we all need to understand is that a prosthetic arm. It's all when you go to these [rehabilitation centers], they measure your right arm or your left arm and they make an arm the same length. With a prosthetic arm it doesn't work because when drinking a can of coke, yes, you bend at the elbow, [but you] bend at the wrist as well so you possibly pull away 3-4 inches. It can't physically work, if it's still made the same length as a proper arm. Because with a can of coke then what happens is, if you don't flex the wrist, you have to physically move your arm in a different position.

R: You have to bring your elbow up.

002: It was just reminded of when I got my Ottobock arm and I tried using it. I was doing little different tasks in the house and I decided that what I was going to try and use this hand as best as I could. One particular day [I decided] I'm going to put the curtain rail up in the bedroom. I got it all prepared, and goto hold the bracket so I could drill so I could mount the

holes in the wall. As I flexed my arm it threw off a separate signal, because my your arm was in a [different] position, it suddenly started spinning that thing around. And I couldn't control it because the sensor in my arm was or the muscle in my arm was touching a different part of the sensor which was giving it a different signal? So obviously it got launched and never was worn again.

S3 Participant 003

R: The first one I'd like to talk about is physical demand. The first stage, so matching the position of the green and red bars with the hand you responded with 70 out of 100. For the second stage, where you were grasping the can with a set force and then wearing the blindfold you responded with 45 out of 100. Then for the final stage, where you wore the hand and used it to pick and place objects, you responded with 55 out of 100. What do you remember about physical demand of using the hand in each of these stages?

003: I remember using muscles in my arm that I wasn't used to using. So towards the end of the exercises it felt a little bit uncomfortable, but not so much that it was painful. I think the blindfold exercise made me feel more in tune with [the prosthesis]. It helped me compartmentalise the feeling and sensation in my arm more than the other [stages]. I think that might have made it more difficult, in a way. I'm not quite sure because I ranked it 45 at the 100, but I remember feeling more conscious of the [sensory feedback]. I think toward the end of the exercises. I became more aware that my arm was aching. Whether that was the use of the muscles that I wasn't used to using or the weight, I'm not sure. I can't quite recall at which one was more a factor. Everything else, from memory, was okay. The strap was comfortable… I think maybe having to twist the arm to pick up objects might have been a factor.

R: Okay. I'm going to ask you about performance now. For that position matching task you said 75 out of 100. For the blindfold task and the can squeezing task that went down slightly to 65 out of 100. Then for the final stage where you were picking up the objects that went up to 80 out of 100. Do you remember what contributed to feeling like you've done well or not on a certain task?

003: I remember feeling a big sense of satisfaction from picking up objects and putting them in a specific place. I think that made me feel like I had more control over my environment, in a way that I hadn't experienced for a long time – since I originally had a prosthesis. The second one I was blindfolded, wasn't I? I think not having that visual stimulus was a factor for me in like having that sense of satisfaction and not being able to, like physically see what I was doing. Although having said that, when I could hear what kind of object it was, that did give me a sense of satisfaction knowing that I'd [identified] it. In terms of what I think I did badly, fumbling with the objects. Not being able to like open [the prosthesis] all the way or close it all the way. Fumbling, like dropping objects. For example, the paper clip. The smaller the object got, the more frustrated I'd get. By the end of the exercises the more practise I had, the easier it got, and I felt like I was doing better. I was getting more used to the types of movements I needed to make. My own coordination as well, I think was getting a bit better. Feeling like I was doing things a bit quicker and more accurately, definitely made me feel like I was successful.

R: Just then you were talking about you felt like you had a sense of control in that you could influence your environment in a way you hadn't felt for... Well, I think you said since you've previously had a prosthetic hand. What prosthetic hand did you have before that and what was it about that that made you feel that way?

003: It was the [split] hook with the elastic bands and can't quite remember the name of it. But you know the one that I mean, you would kind of push it forward and the split hook would open. It was like strap over my shoulder. I remember using that prosthesis to specifically pick up objects and move them and do things like open doors with handles. I think having an additional functionality that I haven't had for quite a few years was made me feel like I could... it was a very specific. Trying to figure out what I'm trying to say… The specific tasks that I was given weren't things that I ever thought that, or the functionality [needed to completed them] wasn't something I thought I needed. I've never thought of myself, as someone who needs the extra functionality that I haven't had for a while. I can get by quite easily just using one hand. But having the experience of picking up objects with my left hand with that [prosthesis] was interesting because it made me feel like there was more it could be like doing or controlling. That's not to say I preferred that. I think I would always prefer to keep going the way that I've been going in terms of using the right hand.

R: That makes sense. Thank you. The next one I'm going to ask you about is frustration specifically. For the first stage, where you are matching the green and red bars with the position of the hand, you had that as 45 out of 100. For the second stage, with the blindfold where you know to identify objects, we put in the hand that was brought down at 10 out of 100. Then for the final stage where you were doing tasks with the hands while you're wearing it, it was 25 out of 100. I think we've touched on it a bit already, but was there anything in particular that made you feel more or less frustrated?

003: It took a while to get the green and red lights to line up, and I think it was like the amount of time it took there was making me more frustrated. Not with you, [R], with my function. It was very easy to kind of flip between one or the other [action], that fluidity made me feel a bit [frustrated]. I think I would have had more satisfaction if it was just like yes or no… maybe that says something about my outlook on life, I don't know. And then yeah, like I said before, I had a lot of satisfaction from picking up objects, like knowing that I'd like got it. That made me feel less frustrated and I felt like I was achieving something; that contributed to me feeling less frustrated. At the same time, dropping things like

maybe feel more frustrated. There were specific objects that I had to pick up that were a lot harder. So for example, the paper clip where it was very difficult to get the fingers to close at the right angle or in the exact place I wanted them to. And I was getting frustrated with the fact that I couldn't quite align the arm correctly with the object, so not that they weren't functioning. It was my control over the hand itself that I was frustrated with.

R: Okay. I'm going to move on to the embodiment statements. The first statement I'd like to ask you about is "the prosthesis in my hand". For all stages you said you strongly disagree – and that's okay. I'm really interested to know what about it didn't make it feel like it was your hand. And what would make something feel like your hand?

003: I'll start with why it didn't feel like my hand. I think it was the look of it; it looked very robotic. It looks like you know how I would imagine a robotic hand to look like. So the appearance is a big factor. Then on the flipside of that, I think that having a more lifelike looking hand would definitely make me feel more like it was aligned to myself. The range of function was less functionality than a real hand would have, so I think that is a factor. In making it feel like whether it's like mine or not because you can look at the other hand and think "well, this can do all this and this, this can't do that". I think it was also the amount of time I'd had using [the prosthesis] – I was with you for less than a day and it was just too short of a period for me to feel like it was part of me. I think with a longer time using it, spending time with it, taking it to bed with you, picking up objects, all of that, I think would personally make me feel more like it was part of me, or is my hand. I think it's probably linked with some of the exercises as well. The exercises were they felt like not 'real' experiences in that we were in like an environment that was in a research lab. They're not tasks that I'd be doing in my day-to-day life. I'm not going to be squeezing an object, picking up random objects. What would make me feel like it was my hand would be to use it in real life scenarios. For example, opening a door or going to the post office and opening an envelope, or wrapping a present or something. In an environment that made it feel like it was my environment, so maybe doing it in my home. Allowing me to choose the objects that I would have been picking up to be something that I was more familiar with or I use every day, I think that would have contributed to feeling like it was more a part of me. Physically, I think the weight was maybe a factor. It wasn't because it was particularly heavy. I think it was just that I'm used to doing tasks without an extra weight on [my arm]. So I think having that extra weight on it made it feel almost more artificial in a way, like there's something there that I'm not familiar with or used to or shouldn't be there. What would help is having something as lightweight as possible.

R: Thank you very much. This next statement is a slightly different angle at a similar topic. This one is "the prosthesis is part of my body". Again, for all of these, you said strongly disagree. What would make it feel more or less like part of your body?

003: That's a good question. I think easy [donning] would help, so not so many straps or things to keep it in place. The additional stuff makes it feel a bit more artificial. Keeping it as simple as possible would probably help with that. The look of it, again, didn't look like the rest of my body. Making it look a bit more similar in skin tone or shape. The shape of the fingers for example, or the palm was a bit like jagged. The [fingers] weren't curved very well, from memory, and it didn't look very lifelike and. I could talk about my body and the fact that you could feel an extra thing there I'm not used to. I think reducing the amount of skin contacts might make it feel like it was more a part of part of my body… I don't know. I think the feedback that I was getting from certain objects was good, but it was up here on my upper arm. I think maybe having feedback at the lower end [of my arm]. I think maybe having the feedback mechanism like at the bottom like where as close as possible to where the action is happening would make it feel more a part of the body. I found it quite jarring having this feeling in my upper arm, when the actual action was happening down at the lower arm. I think making that change would probably make it feel more like it was a part of my body.

R: That makes sense, thank you for that. This next statement is "my body feels complete". Before we started, you said you agree. After stage one that stayed agree. After stage two that went down to somewhat agree. Then after stage three, so after you'd worn the prosthetic hand, it went down to disagree. Do you remember anything about this feeling of completeness and the effect that the prosthetic hand had on that?

003: I think maybe the more hiccups I had, or the more frustrated I'd get with like certain tasks, maybe I felt a mental detachment or aversion to it. I don't know. I think maybe having more familiarity and time with it would be a good thing in making me feel like "ohh there's more function I can be achieving. There are more things that I could do that I haven't maybe been able to do with an exact functionality that this offers". I think that could probably help with the complete feeling more complete. I think my perception of completeness is based on how other people perceive me as well, which probably isn't a good thing. So I think having a device or an extra and that doesn't look or appear lifelike. Makes me feel like it contributes to me feeling incomplete, in a way.

R: Okay. What is it about it looking specifically lifelike that would make you feel more complete?

003: I think it looking lifelike when it's functioning as well as not actually in use. So it might match me symmetrically, for

example. And being able to walk down the street and people not think of it or see it as something that is not completely 100% real. I think that's a factor.

R: The next one we're going to ask about is "the prosthesis is in the location where I would expect my hand to be". When it was on the desk, you always strongly disagreed until you until you wore it, where you said you somewhat disagreed. Do you remember anything about the location of where the hand was relative to your arm that made it feel correct or not for where you would expect to hand to be?

003: I have a feeling that it was slightly longer than my actual forearm. I think it if I put my elbows together and put my arms up next to each other it didn't match. It feeling like it was aligned with the other hand would help. So the size and location. Because the socket that held it in place [extended further] up my arm, it made me feel like it goes all the way up here rather than just what was functioning down here at the bottom of the arm. I think that contributed to me feeling like it wasn't quite in the right place. Not that there is necessarily a solution to that, but yeah, having less straps, less bit that kind of crept up.

R: Okay. The next one is "the posture of the prosthesis corresponds to that of a real hand". Before we started, you said you disagree. And then after you used it so for the rest of the study, you said somewhat disagree. So it went up very slightly. Do you remember much about how it moved or how the fingers looked or how they held themselves?

003: Yeah, it was the range of movement was sadly limited. So it could [close] and twist and pinch with the two fingers and the thumb. I think if it had more functionality then it might have helped. Having said that though, I think that having lots and lots of functionality in a short time to kind of learn it in would have probably been a bit overwhelming, and I would have needed more time to get to grips with it and understood its full range of motion. I think the way that it rotated from memory was like quite lifelike actually. Because very rarely would you rotate your wrist inwards rather than outwards, and I remember really liking that. I thought that was good and lifelike. The shape of the fingers, I think they're quite long and spindly fingers. My fingers aren't long and spindly, so that made it feel like when I was picking up objects, it was a bit more tricky.

R: Okay, thank you. The statement I'd like to ask about next is "I am in control of the prosthesis". At stage one, you said you somewhat disagree. After stage two, that went to somewhat agree, and then that stayed there for stage three. Do you remember this feeling of being in control of the prosthesis and what contributed to that?

003: Yes, being able to perform tasks, and practising and being able to make it move in the way that I wanted it to move, the way I was intending. I think I probably made more mistakes beginning and I found it like difficult to rotate, open, close [together]. I think particularly the closing motion – trying to get it to close a certain amount – was quite difficult. It was quite tricky because it was using certain muscles. The feeling that I couldn't quite control that contributed to me feeling frustrated. I think again, with that it's a practise thing, we had a limited amount of time together. I think that was contributing factor. I think maybe if I had more functionality and I had more practise and did more things with it. Again, maybe a more natural environment or an environment that was more familiar with that would make me feel like I had more control over it as well, because I would be able to do additional things and I'd feel more comfortable in the environment that I was in doing those things. For example, making a cup of tea at home with it, and doing it all with the prosthesis. I think that would have contributed to me feeling more in control.

R: Thanks. The next statement I'm going to ask you about is "the movement of the prosthesis feels like an actual movement". For stages one and two, so when it was on the desk, you said you strongly disagree. Then after stage three, so when you wore it, that went up very slightly to just disagree. Again, I'd like to ask you what factors go into making it feel like an actual movement that you're making?

003: So the feedback helped, but conversely because that feedback was further up the arm, it was a bit jarring. I think feedback was the main thing, having it further down would be a good thing. I think being able to see [the prosthesis] probably helps me feel more confident in my ability to control it. I remember lifting the wrist action with my left arm [to perform a tripod grip]. My upwards was quite uncomfortable. It didn't feel like a natural movement. I don't know from memory whether I was matching it with my right hand at the same time to mimic it, but maybe that would help.

R: Thank you. We've covered all of the statements that I wanted to ask you about. Before we finish, is there anything that you would like to talk about that we haven't talked about so far?

003: No, I don't. I don't think so. I think we covered quite a lot and there's not anything that I think I need to elaborate on. I think maybe the most important factor for me was definitely the environment that I was in.

S4 Participant 004

R: The first one I want to talk to you about is perceived performance. So this is how well you thought you did at the tasks. For for stage 1, when you were matching target positions, moving the hand to a goal position, you said felt like you performed at a 20 out of 100. Then stage 2, after the target forces and then had a blindfold on and identified objects in the hand. You said 25 out of 100. Then stage 3, where you've worn the had done some pick-and-place tasks you said 20 out of 100 again. What it was about each of these tasks that made you feel like you've performed the way you did?

004: Well, I think perceived performance on all three really was just my frustration at not being able to achieve what I should have been achieving. So it was not nothing to do with the test or the hand itself, it was more my frustration. I think that's because, being a split hook user, I'm used to achieving everything each day. So in my mind I'm at a I'm a 90 out of 100. It was frustration. I couldn't... I wasn't able to operate the hand away that I should have been able to. I think the disparity was between my actual prosthetic and how well I use that in my mind, and not being able to use [the research prosthesis] as well.

R: Okay that's pretty interesting. In that case, I'm going to ask a little bit about your perceived frustration to see how it relates to performance. For stage 1, you said your frustration was 50 out of 100. Stage 2 when we were matching the forces and used the blindfold you said it was 100 out of 100. And then after stage 3, where it was picking and placing the small objects or the boxes and blocks, you said 100 out of 100 as well. What's interesting there is that when you just said that frustration was related to your [perceived] performance. Actually, the time when you rated your performance as the worst, which was stage 1, that was when your frustration was also the lowest.

004: [It was a] simpler task probably, in comparison to the other two. It was much easier - it should have been much easier. Then the blindfold thing I just didn't know what on Earth was going on in that... Apart from the can. I remember picking up the can.

R: I'm really interested in your perceived performance on stage 1. Because that was when your frustration was pretty low. So you said that you felt like you only performed at 20 out of 100. What do you think was might have been going on, apart from maybe frustration?

004: Probably getting used to the hand, that muscle movement. Which I felt was quite forced through the three tasks. In my mind, it was a simple task, the first one. Because looking at the screen I can see what's going on. But then when I was unable to achieve that... maybe my competitive edge, perhaps. I just wasn't doing it and it's like, "come on, you can do it". I just felt like I let myself down a bit on that one. All the research that I've done [previously] and all the playing about with this sort of thing, I should have better at that than I was.

R: I'm going to ask you a bit more about the frustration. You said it was frustrating and we touched a little bit about on why stage 1 was frustrating because you've taken part in research before, you've had experience of how you should be able to control these hands or a perception of that. Then you've got all this reference of how well you can do it with a split hook. But stage 2 and stage 3, you've rated as 100 out of 100. The scale doesn't go higher, so I'm really keen to find out what it was about stages 2 and 3. That made them frustrating. Let's start with stage 2.

004: I think I found stage 2 annoying because I had no haptic feedback. It's very difficult to [know what's in the hand when] you're just moving your muscles. Because those muscles aren't trained, I've had no longevity and training to carry out the task. I didn't get any feedback whatsoever, did not know what on Earth was happening, and in my mind I'm thinking "right, I've gotta do this, that, and the other", but it just wasn't working. That little [sound] for the tuna can, then I could recognise it and the bleach bottle, but otherwise I was literally flapping about the wind. I didn't know what was going on because there was no haptic feedback or any kind of feedback from the hand.

R: So I just, I just wanna ask what exactly you could feel.

004: Honestly, I don't think I could feel anything. It was just my focus on moving my arm correctly. I was carrying out the movement in the muscle. I was solely focused on that and I think once you start with that and it gets frustrating, then you're moving your muscles even more. You're putting more effort into this, the pronate or the supinate or the clench. After a while, you all you can focus on is those muscles and you almost lose sight of the hand and what the hand should be doing. I could not see beyond the end of my arm and those muscles moving.

R: Okay, and for stage 3, what did you find frustrating about that?

004: I've done it before and I did the [Jebsen-Taylor Hand Function Test] with the split hook. I kept reverting back to how I did it with my split hook. I know the functionality is different, but I still have that kind of that [completion] time in my head that I need to get it done by. It was that competitive edge, I think, and also knowing that there's a benchmark and I was trying to beat that with the with the hand. Again, the frustration of [when] the muscles were not moving correctly. Even with the best will in the world, I just don't think I'm [able to move my muscles correctly]. I'm almost conscious

not to move the muscles in my arm when I'm in my split hook. Because I know I shouldn't be moving them around in the socket, because the socket isn't designed for that. My arm should just slide in and stay static, really. I think it's just not having the muscles trained well enough to carry out the task. You know, if I was doing this task over the course of two weeks everyday, then I'd have a much better understanding. Also probably the psychology behind it is that I know that no bionic hand is going to give me the functionality in my split hook. At the moment there's nothing out there that can give me what I've already got and improve upon it. So I go in with a – it sounds terrible, but it's no reflection on the hand – I go in with a cynical view. That it isn't going to behave. It's like dealing with a naughty school child. It just won't do what you want it to do, so frustration is through the roof.

R: I see, that make sense. I'm going to ask you some questions about the embodiment statements that you answered between strongly disagree and strongly agree. The first one I wanted to ask you about is "the prosthesis is my hand2. Before we started, you said neutral, then after stage 1 you said disagree, and stage 2, you said disagree, but after stage 3, when you wore it, you said you agree that this is your hand.

004: I think the stage 3, the task was more… I could see what I was doing for a start, and it was more [like] real life. In terms of picking moving things around, that was more of a real-life scenario. That made me think that answer, "yes, this could be my hand", in a future setting.

R: Okay. So what is it about being able to relate to real life that makes it feel like it's your hand?

004: I think you can imagine yourself in a kitchen trying to pick something up with it. In the future, if it becomes really cool and really affordable, and I end up using one I can see how then some of those tasks could be replicated in my real world. Whereas the previous two tasks you have been blindfolded. I've got sight, hopefully that never changes, and looking at a computer screen isn't the real world. So I think yes, stage three was more… I could see a place for what I was carrying out in my in my life. That made me think "actually this could be my hand". Or a bionic hand – not my old hand.

R: Okay. I'd like to ask you about this statement that said "the prosthesis is part of my body". Before we started, so before it was when it was just on the desk, you said you somewhat disagree. Then after we did the position matching, it went down to disagree. After we did the blindfold and the force matching, you said strongly disagree. Then, when you wore it, it went to just disagree again. Could you talk me through any aspects that make it feel like part of your body or not? Or what it was about those tasks?

004: I think it's because it's uncomfortable – the socket isn't comfortable. I think that had a bearing on it, because it just didn't feel [comfortable]. It's as good as we can get it, but it didn't feel like the prosthesis was made for me. I mean, my prosthesis is designed solely around my residual limb; it's custom to me. That probably has a bearing on the psychology that it is mine. That that is my prosthesis or that is my arm, so to speak. A lot of that was the, the uncomfortable nature of the hand. Visibly, it doesn't look anything like something that I would choose. There might be people out there that would choose a red hand. You know what I mean? But that isn't something that I would go for. If it was black and covered in carbon [fibre], honestly, I'd have more of a relationship with it, because I'm used to black and carbon [fibre].

R: Okay, I'm going to ask you about stage one and two. You weren't wearing the hand [during those stages]. It was on the desk. How about then? How much did comfort have to bear on it?

004: It doesn't look inviting to put your limb into.

R: Could you elaborate on that?

004: I think because we were using it [in previous research] we know how long it was. We knew that it was like crazy length. I think, because of the length of it, I know that the myoelectric control and the functionality isn't going to be there. I'm used to having just the end of my arm and then an attachment at the end of it. I look at that the length of my arm [and the myoelectric hand] – it's not crazy long it in comparison, but it's just these are minor details. It's just that little bit longer and I know that the longer the prosthesis, the harder it is to operate. I think that's the psychology of having [my current] arm and using it all the time. Then looking at [the myoelectric hand] and thinking, "that isn't going be the same". I think it does hark back to how I feel about my current prosthesis. That has such a big bearing on how I feel about yours.

R: Interesting, so even before you had worn it in this study you had it in mind that that was how it was going to go?

004: I'm not able to forget about my prosthesis. Probably because I spend my life working in prosthetics. They're centric to my life. It shouldn't be, but it's kind of ended up that way. Because I'm so cynical about prosthetics, or most prosthetics, that has a bearing. I can't shed down cynicism.

R: Okay. The next statement is "I feel as if I am looking directly at my own hand rather than at a prosthesis". This one's quite interesting – stage 0, before we started, you said you disagree. Stage 1 disagree, stage 2 disagree, and then stage 3 that went up very slightly to somewhat disagree. What was it about stage 3 that made it feel more like you were looking at

your own hand rather than that of prosthesis?

004: Probably because of the tasks, because of what you're doing. You imagine your hand doing that... when you had a hand. I think it's all that that was down to the tasks that were being carried out. That made me start to think when a task went well, which was hardly any of them. But there were points when it worked and points when it didn't. But when it worked I was like "ohh actually yeah, that that could be my hand". But that's only a split second because then you do another task and it's not working. Then you want to throw it across the room.

R: Okay. Something about it being useful?

004: It makes me feel like I'm looking at my hand, but not at my prosthesis, because there's fingers and a thumb. I think that had a bearing on it slightly. If it didn't have the four fingers and the thumb, then it wouldn't look like a hand. Because it was all the fingers and thumb moving, at points, to work together. It made me think of my hand... but only when it works. Imagine if you had an arthritic hand then you had it amputated. You would remember how frustrating that arthritic hand was and you couldn't pick things up. So it's almost all the other way around. You've been more frustrated, because it takes you back to when your hand didn't work. My hands always worked before, I didn't have any problem with them. So when your hand worked, it did take me back to having hands. Which is not something that I ever think about. Maybe when I am frustrated at home. Twisting a bottle top, you know things like that. It's just that split second when everything cohesively work together. But that was few and far between.

R: Right, okay. The next one is "my body feels complete". Before we started, you said you agree that your body is complete in its current state. Then after you used the prosthetic hand, it went down to strongly disagree. That was stage 1 and 2; both were strongly disagree. Then stage 3 it went back up slightly to just disagree instead of strongly disagree. I'm interested to know what it is about a prosthetic hand that would or wouldn't make your body feel complete.

004: I've had two bodies. I had one with arms and legs and now I've got a body with no legs, bit of an arm missing, a lot of the other arm missing. I don't know. I'm not even sure what complete even means for me. I don't feel complete when I put my left arm on, for instance, with my right arm. "Body complete" to me is just wearing that one prosthesis on my right arm. I think through the tasks on the third stage, it made you feel like it was nearly... we're on the way to feeling complete. I honestly... I don't even know what complete looks like for me. I guess I feel complete. Yeah, as complete as I can get. It is just putting my right arm on in the morning and getting in a wheelchair. Two devices and I'm complete. When you're missing so much. If I had legs and a left arm and you were giving me that prosthesis, then I would feel complete, cause everything would mirror. So yeah, I'm not too sure what complete even means.

R: It's okay not to know exactly what complete means. Complete means something different to everybody. I would like to touch on something you said, which was when you put your left arm on - So that's an above elbow cosmetic prosthesis?

004: No, body-powered functional.

R: Okay. So when you have two body powered hands on, you say you don't feel complete when you wear that?

004: No, because I don't wear it enough, so it's not the norm. I put it on intermittently to wear a shirt and maybe if I'm going somewhere where I'm going to do lots of self-propelling [in a wheelchair] and I need both arms to make it work. Otherwise, I wouldn't wear it, or I take it to universities for research purposes. It feels like a an add on almost.

R: Okay.

004: Whereas the right arm, I wear it 16 hours a day. That's probably as nearest to complete as I can feel when I'm in the wheelchair.

R: Stop me if this is too asking too much. How about when you're not in the wheelchair and you're not wearing your right arm?

004: Yeah, I feel vulnerable. Say I'm on the floor mucking about with the dog. I feel naked, really, without that arm and not in the wheelchair. I guess the nearest I can get to complete is my right arm on and me sat the wheelchair.

R: Thank you for that. I'm going to ask you about statement seven, "the prosthesis is in the location where you expect your hand to be". Of course, before you wore it, you said strongly disagree. When you'd worn it, you said strongly disagree as well. Could you shed some light on that? About what makes it feel like it's where you'd expect your hand to versus where the prosthetic hand actually is?

004: It just, it just feels too long to me because I'm dealing with a residual limb that's only about 6 inches long, with an attachment right at the end of it. There's a big disparity between the length of that arm and hand, and my arm and attachment. It looks long, feels long. So yeah, because it's almost like the same length again away from the my residual limb, it doesn't feel like I've got great control of it.

R: Okay. The next one is “the posture of the prosthesis corresponds to that of a real hand”. So that’s to do with how the fingers are sitting, how they shaped, how they kind of how they move a little bit. Before we started, you said you agree. Then we did the first task and you said you agree. Then you did the blindfolded task, and it went right down to neutral. After stage three it stayed at neutral.

004: I think to basically to look at before you do any task with it, it looks as near to a hand as a bionic hand could look maybe. Maybe the fingers are a bit too straight. You are still looking at a palm, a thumb, four fingers; to me that’s a bionic hand. Actually, have you seen how much more life like the new Bebionic is? It did look really cool. Hate saying it, but it did look really cool. I know it’s functionally ****, but it looked great. I think your hand would look better if it had some softer bits if you know what I mean. More curvature. I still think [your hand] looked like a hand because of the four fingers and the thumb.

R: After stage 2, after the blindfold, your perception of that changed?

004: Yeah, because I couldn’t see what was going on and I couldn’t make out what was happening. It could have been a split hook for all I knew. There was a big disconnect from feeling that I had thumbs and fingers or something and think and fingers at my disposal. Without any feedback, I couldn’t determine whether forefinger was in comparison to where the little finger was. I just could not connect with it.

R: Without that visual reference it doesn’t feel like the posture really means anything anymore?

004: No, it could have been like those grippers that you get in the penny slot machines in seaside towns, where you grab the teddy. It could have been that for like, for all I knew at that point with the blindfold.

R: Okay and then stage 3, when you could see the hand again, you were wearing it and doing tasks, it remained neutral.

004: Yeah, the fingers just didn’t want to do what I wanted them to do. So I still don’t see fingers and a thumb. They just wouldn’t play ball. It was still a hand, but the fingers and thumb just weren’t working. I wasn’t operating the hand well enough to carry out the tasks. The four fingers, the thumb; you can see it was hand. But just couldn’t quite do what you wanted to do, or you couldn’t get it to move the way you wanted it to.

R: These next three [statements] are all about sensations – what you could feel in the hand. The first one is you “felt a touch sensation in the prosthesis”. You said strongly disagree across the board. How did you feel about a touch sensation with the prosthesis?

004: The only way [I could feel a sense of touch] was because I was looking at it. It was visual otherwise.

R: Do you remember feeling anything from the arm band at all?

004: I couldn’t make any kind of sense that armband, to be honest. The trouble with feedback is it’s somewhere really random. You know, stick it on the top of your arm or, you know, in the back or when your rib cage. That that isn’t how hands work. [The armband] was on skin graft, wasn’t it? So I didn’t [feel anything].

R: I remember you saying that you could feel when the armband squeezed a bit.

004: Yeah.

R: But you couldn’t make out individual fingers.

004: No.

R: OK, so in that case, I’m gonna ask you about the next one, I can “feel the position of the prosthesis”. So stage 1, you said neutral, after you’ve been controlling it to a position. Stage 2 that went down to disagree. And then stage 3 it went back up to neutral. Do you remember what it was that let you feel or made it feel like you could or couldn’t sense the position of the prosthesis.

004: Visual [feedback] and weight. You could feel when it was to the side, you could feel when it was flat. I think that’s just the weight of the arm itself, and the way it was moving the residual limb. The visualisation in the third test, where the hand was positioned.

R: Okay. The next one is you could “feel the force being applied by the prosthesis”. For this one, after stage 1, after the position matching, that went up to disagree. After stage 2 that went up to somewhat agree. After stage 3 it went down to disagree again.

R: What do you remember feeling the force of the prosthesis? It’s that sort of squeezing around your arm.

004: If I gripped tight, it was that, just that pressure on the muscle.

R: Okay. The next statement is “the prosthesis moves the way you want it to move”. After stage 1 you said neutral, so after

that was after position matching. Then after force matching and wearing the blindfold [in stage 2]. You also said neutral. Then after stage 3, it went down to somewhat disagree. What do you think had a bearing on that feeling of it moving the way you wanted to move?

004: It didn't do what I wanted to do all the time. So I couldn't understand what was happening.

R: Could you elaborate a little bit about maybe an example or two of when you wanted it to do something and it didn't do it?

004: [I wanted to] carry out every task that was put in front of me with it in a good time, as well as I use my other prosthesis. That would have been like the ideal benchmark. A lot of the time my muscles were tiring. It would take a long time to carry out what I was doing. There was a lag. Other times where I thought it was going one way and it was doing something else.

R: So a lag and when it would start doing something you didn't want it to do.

004: Not having the muscles trained well enough would have been my fault, not the hands. I remember having trouble with those tasks. I can't remember whether one went a lot better than the others. I remember stacking those connect four pieces. That was frustrating. Maybe it wasn't the movement at the fingers… maybe it's just that there wasn't enough grip on the finger itself for some of those tasks.

R: I see. The next statement is "I am in control of the prosthesis". So after stage 1, you said you agree. After stage 2, you also said you agree, and after stage 3 that went down to neutral. What is it that makes you feel like you're in control of the prosthesis? And what was it in stage three that made it feel like you weren't as in control the prosthesis?

004: The first [stage], I suppose because it was, it was reacting to my muscles moving, so I felt I was in control of it. The third test was because I was not carrying out the tasks well enough. I probably felt that it wasn't doing what it should have been doing, and I felt my muscles were doing what I was asking them to do.

R: That makes sense. Okay, the last statement is "the movement of the prosthesis feels like an actual movement". After stage 1, you said you disagree. After stage 2, you said you agree. Then after stage 3, you said neutral.

004: I don't know why I said I agree in stage 2, in hindsight. I suppose I couldn't see it; I just thought everything was working correctly. Maybe what's happened.

R: Yeah. And then stage 3?

004: Again, just that frustration.

R: What's interesting is the difference between stage one and stage three is it's actually up. So stage 1, you said you disagree, and stage 3 it's up to neutral.

004: Stage one it's on a computer screen – there's no link to real life. Stage 3 it'd go up because there was a link to real life.

R: Okay, I've finished all my questions. But now I'd like to give you the opportunity, if there's anything that you'd like to talk about that we haven't talked about, now would be a great time.

004: My frustration is there's an assumption that myoelectric and is going to be better than split hook functionality. Really, I don't see the cost benefit. Sure, you get the aesthetic [with a myoelectric hand] that people want – fingers and thumbs – but you know, to me as a double arm amputee. I have to err on the side of function beyond aesthetics. A multi articulated limb is just not good enough for what I need to do. I think it's nuts that there are people that are driving the policy, who don't have any concept of the functionality of this equipment. It's just frustrating. That they feel that, because it's myoelectric, it's state of the art. That "it's obviously going to be a better prosthesis", but that isn't the case. I don't know any prosthetists out there that would say that the iLimb or the Bebionic has more functionality than a split hook. And if they did say it, they would be lying. From a research standpoint, I don't see why we're not looking into more hybrid prostheses, whether it be myoelectric or whatever with body powered. It's like we've jumped from a Ford Model T4 to a Tesla, and not tried anything in between. I did quote recently, it was to a journalist, I did say that supplying Ukrainian soldiers with myoelectric multi articulated limbs is like getting in a Tesla to drive through the mud on the frontline. ******* useless. To which my prosthetist and my plastic surgeon wholeheartedly agree. So these guys are agreeing with me in relation to the functionality that split hook, so why more people aren't looking at that? Who is making those decisions? It's not end user led… it should be end user led. I'd like to see more. The amputees are willing to give their time into these research projects and studies. They should have more voice into what goes into the system than anyone else, cause they're giving their time to develop, to understand, and teach. You know, I think we should have more of a voice.

R: Thank you.

S5 Participant 005

R: The first thing I'd like to ask you about is mental demand. After the first stage, matching the bars with the position of the hand, you had that down as 60 out of 100, so about 60%. After stage two, so after squeezing the can and then putting on a blindfold and identifying objects, you had that up at 95 out of 100; nearly as high as it could go. Then when you wore the hand and did tasks in stage three, you have that down as 45 out of 100. Do you remember what contributed to feeling like there was more or less mental demand while using the hand?

005: Yeah. Because I'd done it all day, by the last stage it felt like I was getting into the swing of it. It was like learning a new skill to start with, because I've not had a lot of experience with any prosthetic hands, certainly not robotic ones. So yeah, it was like learning a new skill. Once I had it, I felt more physically tired than I did mentally because I'd got used to using it and I felt a bit normal.

R: That makes sense. What about at stage two?

005: That was because it took away your other senses. So obviously you keep touch and smell and taste and all the other senses, but sight is a big one. Trying to figure out what [object] it was, and my competitiveness wants to get every object right, but it's just not just not plausible, really, is it? Because some of them are quite similar, I found that really mentally taxing. At some objects I didn't want to guess, but I couldn't tell. There were certain objects I could tell what they were. If I had to squeeze tighter to grab it, the armband went tight around your arm and the sensation was around all the fingers rather than just one or two. It was hard to link that to what the hand was doing.

R: Thank you. The next one I'm going to ask you about is physical demand. For every single stage here, you put this as 100 out of 100.

005: Yeah, I found it really, really strenuous for my arm because I'm just not used to using it. I won't say [that my arm] sits by the side of me, but it doesn't get a lot of use. I'm very much dominant with one hand and I was doing everything with the other. The muscles, although they felt pretty powerful when we did the test, repeatedly using them was difficult. The endurance of it was hard.

R: You've given the same response for every stage, but how did you find wearing the prosthetic hand?

005: It was quite hard. It was quite a heavy piece of equipment, so securing it on your arm and functioning with it was difficult. I mean, if there's a chance of making it lightweight, I'd recommend it. The higher up muscles as well, in my shoulder, were tired from holding it up. I felt like I had to... it wasn't a natural elbow lift, it was more about getting underneath it and propping it up sort of thing.

R: Alright, I'm going to ask you about frustration now. At the first stage, the position matching stage, you said 85 out of 100 for the amount of frustration. For stage two, the blindfolded one and squeezing the can, you said 100 out of 100. Then for stage three, where you wore it, you said 55 out of 100.

005: Stage one, it's going back to learning the new skill. I didn't find it easy. Whenever I've done sports I've always picked up a sport pretty quickly, and I just didn't find I was picking that skill up very quickly. So I found it quite frustrating to start with, and then it didn't help that in stage two I couldn't identify some objects. I think it was the bottle and the sponge, because the sponge was so soft, it didn't really feel like anything on my arm until I was closed tight. It was similar in size to the bottle, and I'd try to time when the armband went tight on my upper arm. I just couldn't figure it out. I was trying to use every different part of my brain to be better at it, and I wasn't.

R: Okay and then for stage three, when you were doing the tasks, this went down to 55.

005: I think the blocks, I wanted to be the best at it. I very much was putting a lot of pressure [on myself] and then I couldn't grab two at a time. Well, I could, but not every time.

R: So that was what made it somewhat frustrating. Is there anything that made it less frustrating?

005: Yeah. By the end of it, I felt like I was getting into the swing of it, doing better with the bottle caps and the paper clips and the pennies and stuff. The smaller objects, I felt I was better at picking them than I was the blocks.

R: Interesting. We're going to move on to the embodiment statements now. The first one we're going to do it "the prosthesis is my hand". Before we started, you said strongly disagree. After stage one, you also said strongly disagree. After stage two, you also said strongly disagree. Then after stage three that went up to just somewhat disagree. So I was wondering what was it about stage three that made it feel more like it was your hand specifically?

005: I just want to clarify the strongly disagree, because I knew full well that it's not my hand because I'm a human being. I understand that. At stage three, I felt like it was strangely like putting on a pair of trainers. If I went into a shop and bought [the prosthesis] then it would have been my hand. I felt like I could go in and use it straight away, and I'd be

comfortable using it. I've never used a robotic arm before, and I've never even really seen one in person, so it was very foreign to me right at the start. But then when I was a lot more comfortable with it – by the end of it – I felt like I could go and use it. If I walked into the room tomorrow, I wouldn't be uncomfortable putting it on or anything like that. I'd probably remember how to strap it up and use it.

R: That's pretty interesting. Was there anything specifically about stage 3 that made it feel like that?

005: Yeah. The more I used it, the better I felt about using it. I was more comfortable with it. I don't understand how to explain it, really, but it felt a bit normal.

R: Stages one and two, the hand was mounted on the desk.

005: In stage three, it wasn't just sat on the desk looking at me or not looking at me. I was moving it as part of my arm.

R: Okay this next one is "the prosthesis belongs to me". Again, at stage zero you said strongly disagree, and it stayed that way after stage one and after stage two. Then at stage three, it went up to somewhat agree.

005: I remember feeling like if I did go into the doctors and got a prosthesis, I'd be really comfortable with it. I remember when I was a younger man, well, when I was a child, I had a prosthetic arm. It was very basic and it just did the opening and closing, and I had a battery pack that came through my clothes and stuff. I had one of those and I was never comfortable wearing it ever. I didn't like it. This one, I thought it could definitely increase my quality of life.

R: Okay, and what is it about this idea that it could increase your quality of life that makes it feel more like it belongs to you?

005: It made me want it. It made me want something like it. Obviously [the study prosthesis] was a prototype, but it made me want it made me look into getting a prosthetic hand. I haven't got one, but it did make me look into getting one.

R: The next the next statement is "the prosthesis is part of my body". Before we started, you said strongly disagree. After stage one, so after position matching, you said strongly disagree. Then after stage two that went up to somewhat disagree. After stage three that went up to agree.

005: I felt I was thinking it, and then [the prosthesis] was doing it. It was more of a natural [process]. By the end it wasn't "right, contract this muscle", it was "pick that up". Then I was doing it as a natural reaction. The thought process was different, rather than the physical act was. I was obviously doing the same thing [physically], but my thoughts going into it weren't "tense the bottom muscle to make sure that you squeeze", it was "right, pick up that penny" or "pick that bottle cap up".

R: Okay. So was there a difference between so stage two where you had the blindfold on to stage three? Was there a difference you felt between those two?

005: It was this extra step in the thought process [in stage two], where it was "squeeze this muscle to close the hand". By the time we'd gone for lunch before we got to stage three, I don't know if you remember, because it wasn't round my round my arm, it felt strange. I remember it felt lighter. It was like there was something missing because it was lighter.

R: Very interesting. The next one is "I feel like I'm looking directly at my own hand rather than as a prosthesis". So stage zero, before we started, you said strongly disagree. Stage one, that went up to neutral. Stage two, after you had the blindfold on, that went down to somewhat disagree. Then after stage three, when you've used it, that went up to somewhat agree.

005: So like I said, obviously it's not like I'm looking at what would you say normally is your hand, because it's a bionic arm. But after I'd used it... I can't explain it. It's a weird feeling because I've never had one. I've never had a hand before, so I don't know what it feels like. When people check the backs of their nails, they put their hands out – I felt like that's what I was doing when I was holding out the arms together.

R: Okay, that makes sense for stage three. What about stages one and two?

005: Stage one, knowing that I'd tensed the muscles to get [the prosthesis] to move; to close and to twist. I felt like "I'm in control of this, this is me". Then after you took my eyes away, and I didn't do very successfully without the sight, it didn't feel as much that it was me. It was something else.

R: Thank you. This next one is "my body feels complete". Before we started, so as you are right now, you said strongly agree. Then after stage one and it stayed this way for the rest of the study, you said neutral. What was it about using the prosthetic hand that made you feel more or less complete?

005: I've been born with my arm, so I've never known anything different, so I 100% feel complete all the time. Then I realised that actually I'm probably missing out on something – and it sounds stupid – but like an easier way of living. There are certain tasks that are difficult for me, they're not physically really difficult, but they're more difficult than they should for things such as tying your shoelaces. It takes an extra 20 seconds. Opening a can. Using a knife and fork. I can do it, but

it's more difficult; you have to switch with a fork. You put the fork in, then hold it down with your arm, then cut with your hand. So yeah, I got that first sense of feeling that I was missing out on something to make your life a lot easier.

R: Okay. This next statement is "the prosthesis is in the location where I'd expect my hand to be". For all of the stages while it was on the desk you said strongly disagree, of course. When you wore it, you said strongly agree.

005: Because it was just a prototype, it was very much bigger than my hand, but I have a little baby hands, really. Once I had it on, it was having the full length of the arm. It was more or less where it should be. Usually I use my arm as a hand for clapping and for lifting stuff and whatever, but having the full length, it put the rest of my body back into line.

R: At the end there, you said "back into line". Could you elaborate a little bit more on that one?

005: So if I'm picking something up off the floor I have to really reach down, so it's uncomfortable almost. Whereas with this I wouldn't have to do that.

R: Understood.

005: Say when I was picking the coins off the table, I found my depth perception on that side was very different. I felt like I was going past it a little bit, to pick them up. I'm used to having to lean in, having to be close to something. Whereas then I could be touching it from a distance.

R: The next one is "the posture of the prosthesis corresponds to that of a real hand". Before we started, you said you agree. Then you used it in stage one and stage two and you said you strongly agree. Then after stage three it went down again to agree.

005: Yeah, the fingers are really long. You don't notice that when you just clenching and twisting, but when you come to the finer details of picking stuff up like blocks you notice it. They sort of like get in the way. I was trying to emulate what I do with my good hand, but I couldn't always do it; I'd knock another block over whilst trying to grab one because the fingers are a bit further on than I thought they might be. Which is good, in a sense, because if they are long then I'm noticing that, I'm feeling like it's my fingers that are doing it and the same size as my fingers that are doing it. You understand me?

R: Could you explain a little more?

005: Say I've got to pick something up with my left arm and my finger knocks something. It's because I've gone too far. Then if I'm using this prosthetic arm and the fingers are longer than my other fingers, I would go for the same distance and probably knock stuff off. It's a good thing. It's not good that the fingers are long, it's the evidence that I sensed that there were longer when I used them. It made it feel like it was more like my arm.

R: Right. This next one is "the prosthesis moves the way I want it to move". After stage one you said neutral. After stage two that stayed at neutral. Then after stage three that went up to agree.

005: I think it's because of the finer details. Stage two with the blindfold on, it was hard because it was sat on the desk. Then when I put it on, I could feel… it's weird. I can't explain it very well. It's a strange sensation. I felt it in my upper arm that I was grabbing something, but I couldn't see it. So once I could see it as well, once I was doing stage three... Being able to react to the hand. If I tense too hard or not enough, I could then react to it and tense again. When I was grabbing objects, when I could see the object. I think using your other senses to do things. With the blindfold, I could hear the can; I used another sense [apart from the feedback armband] to identify that that was a can as well. In the last stage there was sight as well, so I had more ways of knowing I was doing it well. Because it was a brand new skill.

R: Okay, this next one is "I am in control of the prosthesis". Now for stage one, stage two, and stage three, you said strongly agree. What was it that made it feel like you were in control of the prosthesis?

005: The fact that you had measured my arm to start with. You'd measured what I could do. Then as soon as I could that I was doing the actions that we tested. And the actions made the hand do stuff. I was like "I'm doing this. This is me doing this". It was instant; that was me.

R: All right. This last statement is "the movement of the prosthesis feels like an actual movement". After stage one, you said you somewhat agree, then stages two and three you both said you strongly agree.

005: Yeah, in stage one, I think it was more of a case of it wasn't doing anything; like it was just closing and twisting, whereas I was actually grabbing things with it [in the other stages]. I was doing tasks with it rather than it just closing and twisting and showing the movement that it has.

R: That makes sense. What is it about it doing tasks that makes it feel like an actual movement?

005: I felt like it was doing the tasks; I was sat there thinking "ohh I'm doing something with an arm" and not I'm not making

that hand close and twist. I’m grabbing things. It went from a making that hand close and twist to grabbing things.

R: Great. Okay, that’s all the statements that I wanted to ask you about covered. The only other thing I want to ask you about, is there anything that you’d like to talk about that we haven’t talked about so far?

005: What you’re doing, bear in mind I’m a 31 year old man that’s got 31 years of not having an arm. If stuff like this can be developed for the younger people, I think it will improve our quality of life massively.

R: That’s very kind of you. Thank you very much for taking part of it, because we can’t do it without people like yourself volunteering for research.

S6 Participant 006

R: Before we start the main questions, here's a quick refresher of what happened. There were three sessions in the study. The first section we showed you a series of target positions for the hand to move to, and they were green bars and you had to move those red bars to get close to them. That session was called position matching. In stage two we did very similar, where we grabbed hold of a can and squeezed on it and that was matching the force. Then we put a blindfold on you and got you to identify objects. That was stage two. Then in stage three you wore the prosthetic hand and did tasks with it, like picking and placing the blocks and stuff like picking up paper clips. Cool, so that's a quick refresher on those. The first statement I wanted to bring up is "the prosthesis is my hand".

006: Yeah.

R: Before we started, you said strongly disagree. After stage one that went up to somewhat agree. Then after stage two, so after the blindfold and grasping the can, it went down to just neutral. Then after stage three, it went up to agree. I was wondering if you remember much about it. What were you were thinking at the time about the prosthesis being your hand?

006: I think it was probably more when you had the sight you could visualise and feel the arm more. Obviously in the second part when I was blindfolded, I was only going off what I thought I was doing and sometimes it didn't feel like what I wanted it to do. When I was grabbing the objects and I was getting the feedback from the [haptic armband] on my arm, I couldn't always feel or tell what I was grabbing; sometimes I could, and sometimes I couldn't, so it didn't always feel like I was in control. But when I had my sight, I could visualise and feel what I was trying to do as well, and I think that helped to use the arm.

R: I'm interested to know a bit about that. You said that when you can see it, that made it feel more like it was your hand. What about seeing it makes it feel more like your hand?

006: I don't know, I just I guess it's cause you're using more [of your] senses so it just feels it because you can see something happening. It just feels more real, I guess. I suppose it's like when you're watching movies, sometimes you feel like you're involved, even though you obviously just sat down and you're not there and… I don't know what you're like, but sometimes I react to stuff moving on the screen, and I suppose it's nice. The visual aspect. It just gets you involved a bit more.

R: Okay, the other thing you mentioned there was that you could feel like you control it. I'm interested in this. What about that relates with it feeling like your hand?

006: What do you mean by that?

R: So you mentioned there that when you took the blindfold off and you could see, you had the feeling that you could control the hand. I was wondering about how that feeling of being able to control it relates with it being your hand specifically.

006: I don't know what to say. Suppose when you try to do something... Obviously with my dominant hand I just move it and I don't think about it, but I suppose when I'm trying to [use] the muscles in my arm to make it do a specific action, and when I can sort of see that while I'm doing it. That probably makes me feel like, "yeah, I'm in control and I'm the one that's doing this" and it kind of feels a part of me then.

R: OK, so like this link between seeing it do what you're telling it to do.

006: Yeah.

R: The next statement I'd like to ask you about is "my body feels complete". At the start, you said strongly agree; you're complete. And then as you [progressed] through the study that actually decreased. So, after stage one it went down to somewhat agree. In stage two went down to somewhat disagreed. Then in stage three, when you wore the prosthetic hand, it went back up again to somewhat agree. I was wondering about your thoughts on that side of things.

006: Can't remember to honest… what was the question?

R: The statement was "my body feels complete".

006: I can't remember. I don't know what that was in regards to. So when we started, what did I say to that?

R: You said strongly agree. So if I asked you now, do you feel complete? Like does your body feel complete?

006: Ohh yeah, I see what you mean... I don't know. I suppose it's again, it's because I've towards the end I could probably see I was controlling the arm. I knew that wasn't a part of me, but I suppose it kind of took away a bit of control from me using my arm to obviously have the prosthesis doing the work for me.

R: I'm not going to press on anything that you don't remember to you all that well, so let's move on. I'm interested in the

amount of frustration you felt while using [the prosthesis]. You put that as very low [for all three stages], you it put down as 10 and then 15 and then 10 again.

006: Yeah. That's probably just down to me just being quite calm and collected. It takes a lot to really stress me out. And I suppose it's cause I knew that it's always a learning process as well. It's like when you're starting your job, you don't particularly get too stressed because you know you're still learning and you don't quite know what you're doing yet. If I knew fully how to use [the prosthesis] and I should be in full control, and if stuff won't work out, then I probably would be a bit more stressed and annoyed. But because I know that it's early stages and it's stuff can go wrong and will go wrong I just shake it off and think "it is what it is" and it'll get better.

R: Thank you. The next statement was, "I feel as if I'm looking directly at my own hand rather than that of a prosthesis". At the beginning, you said strongly disagree. But then [after stages 1 and 2], you said somewhat agree. Then after you wore it, you said agree. It was pretty positive throughout [the study]. Do you remember how you felt about this?

006: I think on the table [before the study] it was it just on its stand, it was just like a floating hand. Quite obviously don't feel like it's part of you. It doesn't look particularly like it should be a [functioning] hand or anything, just look like a model as it was on the table. But then when you put it onto the rest of [my] arm and then you start wearing it. Without the skin texture and clearly looking like an arm, it represented an arm, and that's what you're aiming for.

R: You mentioned the skin texture and stuff like that, how important is that to you?

006: Me wouldn't particularly bother me if it was a close match, but not perfect. Other people would probably [say that] "it has to be a perfect match or else it's just not gonna work for me". That's just a reflection on me; I'm quite chilled and relaxed about stuff.

R: Okay, so was there anything in particular about the hand that you thought that makes it look more like a hand or that makes it look less like a hand apart from the colour?

006: Don't think so. It was kind of what you'd expect in the functions and the movements. Obviously, you could see all of the springs and all of the screws and whatnot, but when the movements are finalised and are in pretty good shape, you'd put probably like a silicon skin over top. Something like that and it'd smooth all out and it wouldn't be far off then, would it? For the shape and the way it looks, it was about right.

R: The next statement this is "I could feel the force being applied by the prosthesis". This is that feeling that you were being sort of squeezed or poked a little by [the haptic feedback armband]. That peaked when after we did that blindfold test. Then when you wore the hand, it decreased slightly, so it went from strongly agree just to agree.

006: Yeah.

R: On that one, do you remember how the feeling was when you were doing it?

006: Yes, when I was blindfolded, depending on what objects I grabbed, so I think it was the can. When that squeeze that as much as I could, it was pressing harder, so I could feel like it was supposed to be gripping something. But the smaller the object got, the less of the sensation of force I could feel him from the feedback. I think when I could see visually you kind of less focused on that feedback. I could feel it sometimes depending what I was grabbing the cans [in the Jebsen-Taylor Hand Function Test]. I could feel it cause it was quite a strong press, but the smaller objects she couldn't quite tell all the time. But yeah, I think when I was blindfolded, I could definitely remember feeling presses around my arm when grabbing the bigger stuff, but the smallest stuff I can vaguely remember it pressing, but it was hard to define how hard or what it was at the time.

R: The last three things I want to ask you about. There was a statement which said "the prosthesis moves the way you want it to move". Similarly, "you are in control of the prosthesis". The final one was "the movement of the prosthesis feels like an actual movement". For all of those, for the first two stages you responded with somewhat agree. Then on the final stage they increased to agree. Do you remember if there was anything different when you put on the prosthetic hand and did the last stage?

006: I suppose it's probably [because] I felt complete. It was a part of me then when I was trying to do stuff and it was reacting to how I wanted it to [move]. I probably said that's why.

R: I'd like to ask you about physical demand. After the first stage you rated it pretty high, at about 80 out of 100. The second stage was down to 45 out of 100. Then the third stage is up at 85 [out of 100], so it's right up there. Do you remember the physical demand of each of the stages?

006: So the first stage was when we were trying to calibrate it and trying to fine tune it. So I think that was cause the muscle groups don't particularly get work that often; I think it was just a fatigue factor. The longer we went on the more tired it

got and the harder it felt. I had to take a few breaks and I think that happened a bit more as we went on throughout the day. The second bit was when I was blindfolded and I was only grabbing objects, so it was only short bursts or what I was trying to do, so I rested in between, which helped and didn't feel as physically demanding. Then the third stage when I was wearing the arm. It was quite heavy as well; you've got that to play with as well as the fatigue of the muscles. I think don't recall any other factors, I'd say that was about it.

R: Cool, that was the last question [related to the results] that I have for you. I did want to ask you a couple of other questions that aren't related to the results themselves.

006: Yep.

R: I remember, you mentioned that you've used prosthetic hands when you were a lot younger. I was wondering if you had any thoughts about how the prosthetic hand you used in the study compares those that you've used before.

006: The one in the study had the rotation of the wrist, which was different. So that was a new concept for me. Function wise open and closing, it was very similar. I think my old arm it just opened and closed. It got in the way, I found, more than it actually helped. Probably cause of the way it just didn't feel like I could do much because I couldn't rotate the wrist. You very limited to the to what you could grab and you've got to move around more to get into a position [where] you could grab whatever you're aiming for. Cause obviously [you use] the flexibility of your wrist to obviously get into the position that it needs to start with [to pick something up]. Also, probably cause I was younger [when I used a prosthesis previously], the arm [in this study] felt smaller than the one I had from the hospital. The fingers felt a bit longer, but I think that's probably cause the hand on the [prosthesis] from the hospital was more curved and yours is quite straight, and has quite elongated fingers. That's probably just probably just a design bit, but it's not the be all and end all. I wouldn't say there was all that much difference between them.

R: Do you remember how young were you when you used to the previous one?

006: I had it from being really young to the first year or two of high school. I'm not sure how old you are when you're in year 7/8.

R: Like maybe 11/12/13.

006: Yeah 11/12, so I think it from probably been about… I did maybe 5 till yeah 12 so maybe 6 years.

R: Okay, you may not remember, but how did it open or close? Was it either open or closed or was it able to go like halfway?

006: Um, it could open or close fully and stop at any point in that, like this sort of range, it just depended which electrode I was pressing to obviously open our close it, so I had a bit of control, but sometimes it just kind of did what it wanted. Sometimes it just opened when you're trying to close, and then sometimes it close and you can't open it.

R: And how did the control of the one we used in the study compared to that?

006: It was very similar in the respect of the open and close. But as I said the one in the study had the rotation as well. I don't really know how many arm is built up, it's hard to say, but I've got quite good control in the end of my arm. That probably helps cause then I can kind of choose what I want [the prosthesis] to do a bit more, whereas if it I suppose if I didn't have that sort of movement or the ability to control it. It would be hard to try and define an open or close cause you can just feel like you're tensing the whole alarm, which would probably give mixed signals, which probably could be what happens with [some] people, I'm guessing.

S7 Participant 007

R: The first one to ask about is frustration. As a quick refresher, stage 1 was matching positions, so moving the hand to a set of positions. Stage 2 was grabbing hold of a can with a certain amount of force and then putting a blindfold on and identifying which object was in your hand. And then stage 3 was wearing the prosthetic hand and doing some pick and place tasks and picking up paper clips and stuff like that. So at stage 1, you said you're frustration was about 50 out of 100, so in the middle. After stage 2, you said it was 30 out of 100, a bit lower, and then after stage 3 you said it was 15 out of 100, so quite low. How much do you remember about feelings of frustration? Could you elaborate on any of that?

007: Yeah, so initially I felt like I got to grips with the movements pretty quickly, but then still within that first stage, what I found was that I was struggling to control between a full hand grip and the [tripod grip]. So that was like the primary source of frustration that I was feeling there. But then as we were going on through the day, I did start to kind of get to grips with it a little bit more and especially into some of those later tasks when we weren't using the [tripod grip] I was really happy and not feeling too frustrated. It was that 1 grip that was causing me that level of frustration.

R: So when we were doing tasks that didn't really require that grip [meant that] frustration went down. So when you found it difficult to control it between fully grasping and doing the tripod grip; what about that specifically made it frustrating?

007: I think how close I knew I was to get in that grip. I think maybe the muscle action that we'd gone for [to perform] the tripod was one that was slightly harder for me to do, whereas the full hand grip was just like a big contraction, a big squeeze. I could do that like 10 times out of 10, but the tripod took a lot more concentration for me to do. Especially when the hand was on the on the stand, I could see that it was starting to do it. Then it would turn into a full hand grip and I'd be like, so frustrated by that, knowing that I was really close. Not being able to kind of finish off that movement.

R: Okay that makes sense to me. The next one I want to ask you about is physical demand. After stage 1, position matching, you put it up at 85 out of 100, so that was pretty high. After stage 2 it was 65 out of 100, so a little bit lower. And then after stage 3, it was 70 out of 100. How much do you remember about this feeling of like physical demand and exertion?

007: Stage 1 was tricky, I guess, cause [I was] using muscles that I don't use very frequently and really having to try hard at that. Force matching as well, like some of those were pretty high targets. So that was a bit of a challenge to get up to there and maintain. It was tricky, it's a longer contraction that I was finding a little bit difficult. And then sorry, just remind me, stage 3 was the pick and place, wasn't it?

R: Yes.

007: So, there's a bit of fatigue in there from actually controlling the weight of the arm as well. Like I found that the box with the cubes in it, that was about as much as I could do without having the sling on. I don't think I've been able to do much more without the sling supporting the weight of the arm.

R: So when there hand was on the desk, a lot of [the physical demand] was coming from your muscles directly, which you don't use often. Then when you were wearing the hand, it was a lot more about the weight of the prosthesis.

007: Yeah. So I wasn't too aware of like fatigue in the muscles that I was using to create the action. It was more fatigue in my bicep and my shoulder that was starting to come into play there from just holding the arm up in that forward position while I was doing the tasks.

R: Okay. The next 1 will ask you about is mental demand. For stage 1 you said that was 80 out of 100, stage 2 that went up to 90 out of 100, and for stage 3 it went down to 35 out of 100.

R: What about the different stages made them more or less mentally demanding?

007: Kind of similar to what I've already said. I think that first stage was like learning it. I really had to concentrate; it was something that was new to me, and I was having to give it my full attention to have success. Then especially while I was having that level of frustration between getting the tripod grip to go, that was really difficult to focus on. But then kind of moving towards stage 3, I actually felt like I was having, like a reasonable success rate like I felt like I was doing okay at the tasks. It felt a little bit more intuitive than matching a position or really focusing on matching a grip level. That, to me, was more mentally demanding than picking up a block and moving it to another side of the box, or picking up a can.

R: That makes sense. I'm going to go into the embodiment questions now. The first 1 I want to ask you about are the statements that said "the prosthesis is my hand" and similarly, the statement that said "the prosthesis belongs to me". For stage 0, before we'd started, they were strongly disagree. After stage 1 and stage 2, for both of those, you put somewhat agree. Then after stage 3, that increased to agree. Do you have any reflections on what it was about using the hands at each of these stages that made it feel like it was your hand, or it belonged to you?

007: Yeah, so stage 1 and 2, I was aware that I was in control of that hand. I was in charge of it. I was using it how I wanted to use it. Then in stage 3, after wearing the hand in a position that I would expect a hand to be, and using it to perform

tasks… I had had the arm on for quite a long time by then as well, and it even got to the point where if you had to like retake control over the hand for any reason. I was all of a sudden really aware of the feedback that I was getting through my through my upper arm, whereas when I had the hand on and I was performing the tasks, that kind of just melted away into the background a little bit and it was what I would expect to feel. But when you were controlling the hand, it felt weird because like it almost felt like you were controlling me, which was really strange.

R: This feeling of you being in control of [the prosthesis]. What is it about that that made it feel more or less like your hand?

007: I mean, that's kind of what owning a body part is like, isn't it? Like you expect it to do something and it will do it. For the movements that I was really comfortable with in that first session, like the grab and the rotate, I felt really comfortable doing those and combining the movements. But then I was still experiencing a little bit of difficulty with the tripod and the hand was sitting on table so I think it was the difficulty with the tripod and where the hand was positioned, that stopped me from feeling like it was fully mine. Having that ability to close the hand and then rotate it. By the end, that was quite easy for me to do, so it that kind of led me to feeling some kind of ownership over the hand. Even more so once it was on and I was having some success with tasks, it did start to feel quite natural.

R: And then I'm going to ask a little bit more about that. So that increase at stage 3, where you say that because you're it was on you and you were wearing it. What about it being on you? Is there anything specific about that, that made it feel more like your hand?

007: It kind of sounds silly, but like proximity to me, like it was exactly where I would expect a hand to be. I have a prosthesis that I use in the gym. And it was a similar length, probably a little bit longer, but not too far from the length that I'm accustomed to with my prosthesis that I use in the gym. There was familiarity there. The feedback almost made more sense because, again, there wasn't that separation between me and the hand - it was on me. Then I was feeling the feedback in my arm as well. It all just kind of like added to the feeling that that was my hand and I was using my hand to grab blocks.

R: Interesting. I'd like to ask you about the next statement, which was "the prosthesis is part of your body". Now for this one, after stage 1 it was neutral, then stage 2 it was somewhat agree. Then after stage 3 it was agree. This is slightly different to it being your hand or belonging to you. So I was wondering if you could elaborate a bit more on what it what it was that made the prosthesis feel like it was part of your body or not part of your body.

007: Yeah. So I think this one was down to like the time that I'd been in control of the hand. Very early on, it was like I was having some success with movements, but it was still very unfamiliar; [it] was a strange thing to be doing. As I got more used to it, I just got more comfortable with that piece of equipment [such] that it started actually feeling more like part of my body. Again, that final stage was actually wearing it and using it. [It was] moving my upper arm to help target something, it just all started to feel more like part of my body.

R: Okay. I'm going to ask you about the statement which says "my body feels complete". Before we started, without any prosthetic hand on, you said you agree that your body feels complete. After stage 1 that that reduced to somewhat agree. But then after stage 2 and 3, it went back up to agree. So I was wondering what it was about any of these stages or using the hand that made your body feel more or less complete.

007: Yeah. Coming into [the study], I don't use a prosthetic for day-to-day life. I use it for like sports related activities, no day-to-day tasks. For me, unless I was in a sporting environment, my body does feel complete. So that's how I felt right when we started. But then, as I'd experienced, using the hand and a little bit of success in some tasks, it did almost make me feel [complete], only temporarily, then once I'd stopped being in control of it, I'd lost a an option for completing a task. Because I was able to do stuff with the hand like I've never been able to move anything with my left hand, even my gym prosthesis relies on me using my right hand to attach it to a bar and then it's good to go. But like that hand felt more able to perform tasks with me controlling it but not having to touch it.

R: What is it about being able to complete a task that makes your body feel more complete?

007: I think that it was like an extra option. I have one hand, [so] I have one option for doing things. Having the arm on gave me another option. Which, I guess, added to that feeling of being complete. I felt like because we had the sling on the forearm for quite a bit of that day. My right hand was out of action because it was supporting the sling, supporting the weight of the arm, but actually I was still able to be independent and do those tasks. Whereas if I ever had anything in my right hand and you said "hey, grab that tin for me". I'd have to put down whatever was in my right hand to grab the tin for you, whereas I was grabbing things with a left hand. I'd never done that before. It was less reliant on my right hand and actually having another option led to me feeling more complete.

R: Okay, there was another comment. You said a couple of minutes ago that you feel complete and then you said except for when you're doing sport. Do you feel less complete when you do sport?

007: Some sports. Yeah. So if I were to go to the gym right now and I didn't have my prosthetic arm with me, I would not enjoy that. I'd feel like I'd be limited in what I can do, and probably feel a little bit more self aware as well. Which is strange when I probably get looked at more when I have like this carbon fibre prosthetic arm on, but to me that is more normal, but actually to other people that's probably more interesting than just a guy with one arm in the gym. I think it a lot of it does come down to like the ability to do stuff. It's what leads me to feel complete or not, so it's quite situational. Like if I was playing rugby, which I do. I don't wear my prosthetic for that because I don't need it. But if I was in the gym or riding my bike, I'd feel quite lost without the arm.

R: Thank you for sharing that. I wanted to ask you about the posture of the prosthesis. So for the statement "the posture of the prosthesis corresponds to that of a real hand", you consistently said you agree. But I am interested to know what would make it look more or less like the posture of it corresponds to that real hand?

007: I guess you don't always rest your hand with the fingers always open and the thumb in that [extended] position. So I guess that would make it feel or look even more like a real hand [in] the way the way it holds itself. And the way the fingers moved for the most part was very similar to how I would expect a hand to move, when it was grasping an object.

R: And what do you mean by that?

007: What I was especially impressed with is the way that if you were grabbing with a full fist grasp, but actually the object was smaller than the full width of the fingers then those other fingers would then continue to close independently whilst the other fingers had gripped the object and continued to squeeze it. Whereas like some prostheses that I've used before, those fingers haven't been independent of each other at all, and once you've gripped something with maybe even just the finger and the thumb, the other fingers will also stop as well, which looks quite unnatural cause a hand wouldn't ever really grab something like that.

R: Independent finger motion.

007: Yeah.

R: Interesting, thank you. Okay, now I'm going to get on to the control side of things. The next statement is "the prosthesis moves the way you want it to move". After the first stage, you said somewhat agree. The second stage you said agree and then after the third stage you said strongly agree. What was it about how you were using the prosthetic hands that made it feel like it moved more the way you wanted it to move?

007: So stage 1, it was that that tripod grip that was stopping me from agreeing more because I was doing what I thought was the muscle activation that we'd calibrated for it and it just wasn't doing it. But then as we went through the stages, I either became better at actually doing that or even later on in the study we didn't need that movement. So kind of a combination of, early on, frustration with that specific movement, but then later either getting better at it or not using it as much and that that made me feel like it was doing what I wanted it to more.

R: That makes sense. Similarly we had the next statement, which was "I'm in control of the prosthesis". So for this one again, you said, somewhat agree after stage 1. Then you said agree after stage 2, but this time after stage 3 it stayed at just agree.

007: OK.

R: [Your response] didn't go to strongly agree like the other [statement]. This is slightly different. This is control the prosthesis versus the prosthesis moves the way you want it to move. Why do you think was?

007: Yeah, again, I think it's very similar for stage 1. As in, it would sometimes move in a way I didn't want it to move in, which led to me to feel out of control. Then I guess perhaps what has stopped me from saying strongly agree in stage 3 is that actually there were times where you were in control of the of the of the hand. Just because little bits needed doing to it [for calibration] or whatever, I think that probably disturbed my feeling of control at all times.

R: That makes sense.

007: And actually, the feeling of – and I've mentioned this already – the feedback on the arm when you were in control of it was quite strange. It felt like you were controlling a part of my body at that time. Which I guess would take away that feeling of control for me.

R: Yeah. I'm gonna come back to the feedback and that feeling because I'm really interested to your thoughts on it. The last one on the control section is "the movement of the prosthesis feels like an actual movement". After stage 1 you said agree, stage 2 was agree, and then stage 3 was strongly agree. Wha was it about the movement of the prosthesis that made it feel more or less like an actual movement?

007: Stage 1 and 2, I could see that that those movements were what you would use if it was on the end of your arm. Then stage 3, I was actually using it and it was working. So rather than just seeing those movements being useful, I was

actually completing tasks with them and that made it feel that way.

R: So in stage 1 and 2 you were saying you could imagine it on the end of your arm?

007: I don't know if I was imagining it on the end of my arm, but what I could see were movements that I would use if it was on the end of my arm. Like grabbing something and turning, I could see how that would be useful. But then, especially when we were picking up those cans at the end, the heavy ones, the grab and the turn, it made a lot of sense when it was on the end of your arm and it worked really well.

R: Okay, so the first [stages] were kind of imagining how it could be useful?

007: Yeah.

R: And then when you're wearing it, it was being useful?

007: Yeah.

R: Okay. And what was it about that usefulness or functionality that made it feel specifically like an actual movement?

007: I think because it replicated the movement that I would do with my right hand. On all of those tasks… I have slid something with my finger and thumb off a desk before or I have picked something up and then turned it to look at it. Perhaps not turned it to be able to pick it up, but I picked it up and I turned it. So [the movements] were replicating movements that I do in day-to-day life.

R: I see. Okay now for feedback. This statement is "I felt a touch sensation in the prosthesis". After stage 1 you said somewhat agree. Stage 2, you said agree, and after stage 3 you said strongly agree. What could you feel that made it feel like a touch sensation?

007: The squeeze on the upper arm and the intensity of that squeeze, depending on how hard I was trying to grip an object. But then as we went further, through the activities, I started to become more aware of how that corresponded to different gripping actions. I think I did okay on that identifying objects task, and that was where it started to become a little bit more natural. That was like I was able to feel stuff. Then especially in [stage] 3, I think because I'd had the arm on for quite a long time, by then I was getting a little bit more used to the whole feeling of it. That sensation of touch or feel became expected, whereas in the first part it was "ohh that's like a weird feeling". But I became more used to it and started to pick up a little bit more detail from that feeling.

R: Why do you think it became expected? Because I remember you said earlier that when you put it on, it was something that was expected, whereas on the desk it was something that was a bit weird.

007: Yeah, I think proximity again. The hand was attached to me at this point, which made it feel more logical that I would feel something from grasping an object with it, whereas when it was on the desk, I know I was connected by wires or whatever, but I wasn't actually attached to the hand, so it kind of felt like I was feeling something that was over there, not near me.

R: That makes sense. I'm also interested – this is slightly separate to the touch sensation – when you were wearing it, could you feel that the end of the prosthesis would, say touched the table or contacted logged by the amount of like weight that was put on your arm?

007: That was harder, to be honest. I think I was well in tune with the feeling of squeezing an object, but something that I had particular difficulty with was knowing when the thumb had touched the edge of the table. Yeah, I did really struggle to feel that and even the perception of the length of the arm did make that a little difficult. Yeah, I did, however, feel more in tune with that sensation before I put the sling on, so in the very first task, moving the cubes from one side to the other. There was that that [divider], I bumped that once or twice and I felt that, because it made the whole arm kind of move a little bit. And then when I was placing them on the other side, I could easily feel when the weight of the arm would have a bit too much, but for that one I could definitely feel it, yeah.

R: Okay so the next one is "I could feel the position of the prosthesis". For this one, for all stages you said that you somewhat agree. How did you feel in relation to the position of the prosthesis?

007: Yeah, I found it harder to feel the position when it wasn't grasping an object. I think if it was touching something I was getting more feedback through the through the band on my upper arm. Whereas in terms of position, I was relying very much on sight to approximate and see how many degrees I'd rotated the wrist in that position matching task, that was the way I was doing it, rather than feeling where I'd put it.

R: Aha, okay. The last statement about sensation is "you could feel the force being applied by the prosthesis" for this on you said agree, agree, and then strongly agree for stages 1, 2, and 3. I think we've touched on it a little bit already, but what was it that specifically made you feel like you could feel the force?

007: Yes, it was that kind of like total sensation of a squeeze on my upper arm. And I felt like I picked up on that really quickly and it just made sense to me. And then I think the reason it went to strongly agree was that I just had more time with it on and I was beginning to like build a picture in my head of what that level of squeeze meant in terms of the force that was being applied. That force matching task as well did help a little bit because I could see on the screen when we were having that first little play around with it, I could see what my output was versus the target but I think the reason it went to strongly at the end is because of the amount of time I had been wearing it.

R: Okay, so this this one is "I feel as if I'm looking directly at my own hand rather than at a prosthesis". After stage 1 you said neutral. Then after stage 2 that went up to somewhat agree. Then after stage 3, you also said somewhat agree. Was it about the hand that made it feel more like you were looking at your own hand or not?

007: It definitely started at neutral because it was this big red hand on a desk and you could see some of the motors that are on it and the cables. But I very quickly realised that it's more than that; I was in control of it. So that kind of balanced me out to go to go middle. Because it was unlike any prosthesis that I've used before, and I think as my familiarity with the prosthesis grew, it did make me feel like I was looking at more of a hand than a prosthesis. But, I couldn't get past the fact that it was red and slightly bigger than my hand and yeah, had all these motors on the back of it and cables poking out of it. Yeah. So that's what stopped me from putting strongly agree.

R: Okay, that's Interesting. I mean, it is a big red hand made of plastic. I'm interested in stage 2 and stage 3 – you gave the same response. For other aspects of this, for example, for it belonging to you or it being your hand, you said that this proximity made it feel more like your hand. But what was interesting is this result in particular stayed the same. So I was wondering if you had any thoughts on that?

007: Yeah. So it's exactly that like it did, like the proximity made it feel more like my hand, but it didn't change the appearance of it, it was still exactly the same look.

R: Okay. So a lot of it's about aesthetics for that statement in particular.

007: Yeah, but that's not to say that I would want it to look anymore like a real hand like for me. I appreciate a prosthetic that that does a job, rather than looks like a biological hand. That's just never been what I've looked for in a prosthetic, cause I I've had cosmetic limbs that have done very little in terms of practical tasks or helping me do anything in day-to-day life, and in actual fact [were] a bit more of a hindrance than anything else. But the prosthesis I have now and that I use all the time is like a black carbon fibre forearm with a titanium hook on it. It looks nothing like a hand. It looks nothing like an arm, but I still refer to it as like my arm. I don't expect it to look like a hand – I don't want it to.

R: Why don't you want it to [look like a hand]?

007: I'm not trying to hide the fact that I have one arm. What I want from something is to help me, and if it helps me and it needs to look… funky, then that's completely fine. If there was a hand that… actually no. Sorry, I was gonna say if there was a hand that was perfect and it looked like a real hand, I'd wear it. But I actually don't think I would. I think I'd rather it be clearly identifiable as a prosthetic hand because actually me having one arm is a big part of my identity and I wouldn't want to get rid of that, or have people unable to spot that.

R: Thanks for sharing that, I think it's really important for us to capture that.

007: Yeah, but like you see it with a lot of a lot of leg amputees. Very few prosthetic legs that I see people wearing day-to-day look like a biological limb, and loads of people wear shorts with them because [they] don't try to hide anything. In actual fact, that sometimes it's almost a bit of like, "hey, look, I cool my leg or my arm is", rather than trying to mask the fact that they are using a prosthetic limb.

R: Yeah. I would like to ask is there anything we haven't talked about yet that you would like to talk about?

007: I don't think so other than I really enjoyed the whole experience and the challenge cause it was difficult at times, but I really enjoyed that aspect as well. I found it really fun and exciting to be able to use a bit of tech. It's really fun. I enjoyed it and yeah, exciting.

S8 Participant 008

R: Okay, so the first thing I [want to] talk about or I want to ask you about is. We had this question. The statement that we asked you, which was “the prosthesis is my hand”.

008: Yeah. Yes, I do.

R: And with that one, before you started, so in stage 0. You had it down as neutral. Then after stage 1 that went up to somewhat agree. Then after stage 2, right down to disagree. And then stage 3, it went up to agree. So, it went all the way up again. I just wanted to sort of understand that bit more. Would you be able to tell us a bit more about it?

008: So remind me again, what stage 1, stage 2, and stage 3. Was stage 2 the feeling?

R: It was, yes, the stage 2 was squeezing onto that tin can and then putting a blindfold on and identifying objects in the hand.

008: OK, so stage 1 I scored as I did because it’s just getting used to it, and so I couldn’t really get a sense of what it was I was supposed to be feeling if that makes sense. And yeah, as I said, just just trying to get used to it so it was a neutral comment because I didn’t know what I was supposed to be feeling. Stage 2 I would have scored like that because I’ve got no sense of what’s what so there’s nothing to say. And then stage 3 I scored as I did because I was able to use the hand and connect with it slightly. In order to complete the tasks that I was given.

R: Okay, so I’m interested in stage 3. So, in stage 3 there you said that you were able to connect with it. And I was wondering if you could sort of elaborate a bit more on like how that’s related to this feeling that this hand is your hand.

008: So, by thinking of a movement and an action in my head, I was able to send that to the hand. And in order to do the movements and pick up the pieces so you have a connection with it, because I’m telling it to do something and it’s doing it. So, I feel that I do have a connection with the hand and that I’m able to use it as if it were my hand. If that doesn’t make sense, is that answered that?

R: Yeah I can. I can see that so it’s sort of – correct me if I’m wrong – it’s a sort of like this feeling that you can control the hand sort of makes it feel more like it’s your own.

008: Yeah, yeah.

R: Okay, and so for stage 2, would you say that? Well, what was happening? What was happening there?

008: So stage 2 with trying to feel it, you know, and you took my eyes away from me. I can’t, obviously I am. I’m able to move the hand by using my sight, but once you took that away from me. I have no sense of what I’ve got in the hand, so I don’t feel it so it doesn’t feel part of me. I’m seeing with my eyes when I’m moving the hand and doing the tasks, but when it comes to actually touch and sense I didn’t have a lot of it. I was guessing that’s why I scored so low. So I’ve got no association with the hand no matter what I was trying to do and think of thinking my head. I was just guessing so and the scores show that. I didn’t have a clue what I had in the hands, so. Unless of course it was a tuna can. Ohh the bleach bottle.

R: So the one that made sound.

008: Yes, I’m using my other senses. In order cause that’s how I adapt, I use something else and my sight and my ears is what I was using to try and guess. But yeah, when you take my vision away from me, I didn’t have a clue hence why I don’t feel connected with the hand and I didn’t feel it was with me or mine.

R: Gotcha. OK so I’m [going to] draw your attention now to couple other statements. Statement 13 “I am in control of the prosthesis”. So I just wanted to understand that bit more about this sense of control of the hands cause what’s interesting is on stage 1 and 2. So after position matching and then after this feeling [stage] you said that you actually somewhat agree that you were in control of the prosthesis. So [after] stage 2 your feeling that you could control it didn’t actually drop. So I was just wondering if you could explain a bit more so when you were doing that blindfold test, did you still feel like you could control the hand?

008: No, because I couldn’t see it. So I find that in order for me to tune in to control that hand, I need to look at it and feel that that hand is mine and sort of send the message from my brain to that hand to say, right? These are the movements that we need to do, and that’s I use my vision to do that. So that’s why then when we started doing the picking the penny up and the and the paper clip. Because I was able to see. Okay, I need to be able to use this much. You know precision and I’m using my eyes and feeding that back through the movement, so that’s how I was able to connect with it.

R: OK so this feeling of control. Stage 3 was all about doing tasks with [the prosthesis]. Would you say that that had an effect on your feeling of control?

008: Yeah, I felt like I was able to control that hand and I felt like I had a good level of control because I was able to instead of just being robotic and I was able to precisely [external interruption]. And yes, so I was able to control the hand as

was needed and so I felt it was. I was connected to it and it was part of me because I was able to give such level certain precision to be able to pick that up cause it I mean it was difficult. It wasn't without difficulty, but I was able to do it and I don't think that if you didn't feel attached or it part of you, you wouldn't be able to do that.

R: Interesting. Okay, so it's perhaps it's kind of tied to being able to see you doing things with it.

008: Yes, you're I'm getting the feedback from OK, so if I move too fast or if I think of the action too fast. It doesn't work, so I have to give. I'm relying on the feedback from my eyes to be more gentle, you know. And then obviously listening to your instruction and just trying to then relay that from my brain through my eyes into the hand. So yeah, I do have a connection with it.

R: Okay, interesting. Yeah, so I'm just gonna ask you about three statements this [time]. One is "I could feel a touch sensation", one is "I could feel the position", and one is "I could feel the force being applied". And after stage 1 where we asked you to move the hand to a certain position, you said you disagree. You disagreed with all those statements actually. And I [want to] start off by just asking you sort of what was making you feel that way.

008: I disagree? Sorry, can you repeat that? So I disagreed with...

R: You could "feel a touch sensation in the prosthesis". You could "feel the position of the prosthesis" and you could "feel the force being applied by the prosthesis".

008: Yeah, no, I couldn't feel anything.

008: In terms of like what I could sense, if I wasn't looking, I wouldn't know. I could only guess. So, say for instance if I wasn't looking at the hand and I went to make the movements. Then I wouldn't know if I was being successful or not unless I could look at it so in. So that's why I'm not feeling it.

R: Yeah.

008: Like in my body, through my sensory sort of you know I'm not getting no sensory feedback from it. That doesn't make sense.

R: In that case I'm then going to ask you about after stage 2. Where actually said you somewhat agree with each of these.

008: This is when I could see was it?

R: No, stage 2 is when you couldn't see. So, after stage 2 you actually said these actually increased. And I remember you saying at the time that you were feeling sort of like a tingling in your hand. And I want to understand a little bit more about that and what that was.

008: So, I think as it went on and I started to tune into it a little bit more. I don't know if – see it's really hard to say whether I was actually feeling it. Or what it was I was feeling because there was tingling. Like now, when I think about my hand, I'm feeling a tingle in my hand that's no longer there, so I don't know if I scored that way because I have. I know what the task is. I know what I've got to do. And so am I pushing that into the hand and making it tingle. I don't know, hang on, I'm going into a rabbit hole here. Half of it is I don't know if I'm really feeling it or if I'm willing myself to feel it so. I want sort of. I don't know [I was] making it up almost. Does that make any sense?

R: That does. It's. It's an interesting mix of like wanting to feel something in the hand and [actually] feeling something in the hand.

008: Yeah, cause it's like you know they say, "you asked for the universe for something to come back and it comes back" like that sort of stuff. So, am I sitting there thinking "yes, I've gotta concentrate" so is that a sensation? Am I overthinking it and thinking yeah, it's happening or is it really happening so it's hard to try and distinguish between reality is it really happening or am I cause your brain's strong? Am I really wanting it to happen? So, I'm thinking that it's happening. That's like mind blown, but so I think sometimes I can get a sense of something's going on and you'll get different tingling sensations. But I don't know if that's just the way the injury is, and the message is going through. I don't know. Sorry, thank you.

R: No that's alright. Please don't apologise.

008: As clear as mud.

R: No, all of that made sense to me, at least.

008: Yeah, it's it's hard to try and express it because. You want it. I want it to. I wanna feel it I wanna do it so much that sometimes I think am I just imagining it like you know you just you tell yourself that and then that's what it is. But in reality, it's something totally different so I find it hard to distinguish between that. But I definitely did feel like a flicker or a flutter. But what it was, I don't know. It's like when I. The only way I can maybe explain it is when I get the nerve pain

so my brain thinks move hand. And because the hand's not there it comes through as a pain because the messages are mixed up, I don't know so was I trying to think "yeah I can feel that" so there's a little flicker there [is that] my wanting it or is it actually happening?

R: So, to put that in kind of like a scientific way. It would be to say something like it's eliciting a sensation in the phantom limb.

008: Yeah, yeah, I'm willing it to happen, but how do you distinguish that it's actually happening? Or is it just that I'm telling myself that it's happening? Is it an illusion that I've created because I want it?

R: And just to understand a little bit more about why that might be happening. You had some reconstructive surgery on your nerves?

008: Yes, so they've done nerve and muscle transplants from my legs into they put it up into the chest and for it to then grow so [targeted muscle reinnervation] to connect with any of the nerves that were OK in the brachial plexus for it to then grow down to the stump, hopefully to try and help alleviate pain and then so then [the doctor] said that "you know these things take a while to grow", so could it have been that I was thinking? This is a movement, and that little fluttering was the nerve endings being there. So it could be that it is actually physical and not me imagining it. Because I've had so many surgeries to try and reconstruct, it could be that it's a slight flicker and I'm then just adding on to that with my imagination.

R: Interesting so you may have felt some sort of sensation.

008: Yeah.

R: But identifying what object it was out of the question?

008: Yeah.

R: Okay, I'm gonna ask you about another one. This one, the statement is "my body feels complete". So this is a really interesting question. This is the sense of completeness and what it means to you as a participant. At the beginning, before we started at all you said you agree. And then we did session 1 [which] was moving it to a position and it went down a little bit to somewhat agree. And then we did session 2, which is the feeling one and it went right down to disagree. And then when you used the hand [in session 3], it went back up to somewhat agree. So, I was just wondering if you could elaborate on any of that.

008: This was when I was moving it and it and that it felt part of me, yeah.

R: So this is your body feels complete. This sense of completeness.

008: So when I was moving the hand slightly. You do have a sense of you are complete. Very slightly because I've got an association with it and I can whatever I'm pushing from my brain is causing that to move. So yeah, and then it does make you feel like you have two working hands again. And then, but then obviously when it come to sensing, I can't feel anything. There's no appendage there, so that's why the scores went down and then back to when I was able to move the hand. Yeah, I suppose you do feel slightly complete because you've got two working hands again. I've not answered that.

R: You've done great. I just wanna poke a little bit more at it to do with this. So when you had the blindfold on so it was, you know I'm interested about this. Sort of like this visual connection between you and the hands. Would you explain a little more; so you're saying like when you've got it on you it feels like you've got two working hands again?

008: Yeah, yeah. So when I've got the hand on and I can see it because whatever movement I'm thinking it's causing that hand to move so that in in a in a way it feels like it's part of me because I'm controlling it so it's me that's doing it. And then when I when I can't see, I can't sense it. I don't know that it's there, so I have no control so I don't feel like it's part of me and then back to when I can see, and I was doing. The more the tasks that demanded more dexterity. Because I was able to control it in such a way, yes, it did feel like it was part of me. So, it completes you. If you know what I mean without sounding a little bit, you know. Yeah, so it does feel like it's part of you.

R: I'm interested as well. In stage 1 you were controlling it and you could see it, but it wasn't on your body, it was on the desk, whereas in stage 3 it was on your body, but both of these have the same completeness score, so I was interested in hearing a bit more about your thoughts on that.

008: I think that's because I've got no. I don't know. I've got no sense of touch on the stump, so it wouldn't matter if it was on me or not on me. The fact that I'm controlling it is why those scores are similar is because I can see that I can move it, whether that be on the desk or on my body. So I feel like I've got a connection with it.

R: Gotcha. I'm gonna probe a little more on this – I don't wanna be too annoying with it – but so that you this feeling of control and this connection feeling, why do you think that relates to making your body feel complete?

008: Because I have the ability to move something and do what I need to do and so it's more helpful to me and so. I don't know if I would feel I like, say, for instance, in an ideal world I get the hand and I'm able to use the hand, so I would feel that I'm more independent because I can move this hand. Does that make sense? You wouldn't have to ask for help, because I can do it myself so that that's the sense that I feel that you would feel complete, because if I was at home, wouldn't have to ask anybody to help me cause that sort of makes you feel a little bit more complete does that.

R: Okay, so this idea of being independent.

008: Yeah.

R: I'm going to probe it again on that. What do you think it is about being feeling like you could be independent that makes you feel complete.

008: Because if you're on your own and you're stuck. Uh, and the one that I'll bring it to what I relate to the most is food preparation and if I'm on my own and you know I haven't got my mum or dad there. If I had that hand there and I'm able to grip with that hand to open, then I don't have to ask anybody or I don't have to go. Ohh I can't do that because I'm unable to open it, you know and it's them little things that really frustrate me and calls me such annoyance. And when it "****es me off" is the best word to say, because I can't do it myself and I used to be able to do it myself so to have something that goes actually I don't have to ask anybody because I am fiercely independent and I could open it. It makes you feel complete because you can go about your, you know on your own without having to ask anybody and you don't feel weak. You know that's my big hang up like a feeling weak because I can't do it and I have to ask for help. You know, you do have to, I will ask, but I just don't like it cause it I just wanna get on with it instead of having to you feel like you're being a burden, you know.

R: Thank you for that. That was a lot of information, I appreciate it a lot.

008: No problem.

R: I wanted to ask a little bit about this question which says "the prosthesis is in the location you'd expect your hand to be in".

008: Yeah.

R: And so, when you wore it, I just wanted to know a bit more about. What did you expect, like where do you expect your hand to be sort of? How does that line up? What goes on when you think about "this is where my hand should be"?

008: I'm so when I had it attached to me. I felt that it was the right length. As to where I would expect it to be, because if it if it wasn't where I would expect it to be, then I wouldn't have been able to control it as I did, because if it was too long or not where I want it, yeah, as I said, I wouldn't. I wouldn't be able to be able to move it and work with it to do what I wanted to do. If I didn't feel like I had that connection. So that's why I answered that way.

R: Thank you. There are a few questions we asked you also about, so it's frustration and sort of physical demand and stuff like that. I'm going to ask you first about frustration. So, in the first, the first session where you are moving it to positions, you said the frustration was really low, at 15%.

008: Yeah.

R: Like 15% and when you put the blindfold on your frustration when up to 80%. And then when you were using it to do pick and place tasks, it was about 50%. So I was just wondering about each of those tasks what it was that made you answer in that way.

008: Okay, so the first task, because it was an easier movement to do and I felt like I'd sussed that movement. You know I didn't feel that I was struggling too much with that. The second task is I couldn't feel it, I couldn't see it and it's really it's annoying cause I wanna succeed and I wanna be able to do it and I can't. So that's why it shot up. And then the last one I knew I was doing it and I was capable of doing it, but I was giving myself [pressure]. It was the point of giving myself time to try and be as precise as possible and slow it down a little bit and then what happens is you're dealing with the fatigue at that stage. So you're trying your best to do it, and your body's not sort of doing what your brain wants it to do. Um, so that's why the frustration got a little bit. I'm tenacious, I don't give up, so I was determined that I was gonna do it. But yeah, the frustration is high, but purely because I know at some point in my life, I've been able to do it with no problem at all and in my head I've still got it hard wired. I should be able to do this, but you know, as you said I had what just a few hours session testing with that. If I had the you know if I didn't have to work or what not and I could just practise and train then. I know that I would be able to use that efficiently and that and that's the massive difference. If we were given the care and the time to practise and use these things, then more people would use their prosthetics because they know how to.

R: There's a lot that, okay, so a lot of factors go into that. I'm gonna try and do each one by itself. I think the first one you

mentioned was that you felt some fatigue.

008: Yeah.

R: And that was kind of making you feel frustrated. What about fatigue Is it that makes it more frustrating?

008: Because the must, I think the part is you're tired so you know what you wanna do, but it's just trying to get your body to do it. A lot of where I've had muscle transplants and the muscles have muscular atrophy. The muscles are weak. It's not as strong as it could be, but I know that I can build that muscle to be stronger, but I'm just not there. I'm just impatient that's my personality, which isn't a bad thing cause it pushes me to do what I've got to do. So I think in terms of the fatigue it was about giving myself the time to be able to do the task or just take a breather. You know, sometimes I find myself and holding my breath it's not helpful.

R: OK, the next thing you mentioned was how you had this reference that you could do it before, and that you want to be able to do it now and it's frustrating.

008: Yes, so you know I went 22 years with 2 hands and I was able to. You have the dexterity you can just do things without thinking about it. Then it's taken away with me for like 23 years. So you then become adept to using just the one hand, and then when you go and start to use 2 hands again, you're just you're switching your brain back and your brain, then I don't know if it's muscle memory or what it goes back to "Ohh I'm 22 again. I've got 2 hands", but it's not the same, so it's [about] trying to tell yourself, "OK, there's something there, but it's not the same" and it's trying to give yourself time to learn that, so that's what I meant by that.

R: And what about that made it frustrating?

008: Because I thought I should be able to do it, I don't give myself the time. I thought I should be able to. I thought like everything I approach; I think "I should go in and smash that, I should be able to do it", but it's not. No marathon runner went in and did 3:30 did they? You know, they practised, they practised, but I'm very impatient and I just wanna do it and succeed and so I'm not giving myself the time. I wanna go from A-Z as quickly as possible without doing whatever it needed in between. It doesn't work like that. Well I still try anyway, even at the age of 45, I've not learnt this. So if I can work on that sort of thing. That's a brain remap that one.

R: Interesting. Okay, now there was one where we asked you about temporal demand. So we asked you about how hurried or rushed he felt while doing the test on all three tasks you put down roughly the same. So, I think you put 70 for the first 2 and then when you're wearing it you went up to 80.

008: Yeah. I think because I know what it's capable of but I wanna do it so badly that I'm not giving myself the time to do it and so I'm like come on and then you get frustrated and then you can't think. So that's when you know you need to take a step back and just try and calm, but that's me. That's something that I need to work on and going forward for me to get these [prostheses], I need to realise it's gonna take time. I'm not gonna be able to put it on and be really like fluent with it like it's my organic hand and that's that's difficult. And because I've been working so hard to try and get this hand, I think "ohh, I should be able to use it", but it doesn't work like that. And that's why I think. [external interruption] Yes, so I've been working hard to get the hand that what I you know when I eventually get it, I need to understand I have to give myself time what with it. So even though you've got the hand, it's like right, this is gonna take a while. You know the journey hasn't finished. Cause you've got to now teach yourself you've got 2 hands and they got work together and so that you know what you were saying about and the statistics showing that [often] people that do have [prostheses] don't really use them, and it's because you need that sort of care and assistance in the middle to show you how to do it and the time that it takes, and to be able to give yourself time to use it properly. So I think that if there was some sort of treatment or programme where people do get their prosthesis to sort of hold their hand, pardon the pun to show them how to use it. More so it wouldn't be wasted.

R: Okay, so it's this this thought about after care?

008: Yeah definitely, because that's the thing and the first surgeon when I was with [the hospital] where my accident first happened and when I found out about prosthetics and I went to [the doctor] and I said "what about a [prosthesis]" and they went "ohh no you'll just get bored and leave it in the drawer" and I was just like "well no I won't" but that's actually got a bit of truth [to it]. I suspect that's what people do cause they gets so frustrated they can't use it like they would think that they would use it. You know then they need that support package to go right? OK, we do little bit by little bit hour by hour. You're not gonna just put it on wear it all day and be able to do everything you used to do cause that's what I would want but you can't.

R: That's really that's really interesting to hear from my perspective as well. Thank you for that.

008: Purely because I know that I would feel I'd be like, well, why can't I do it now? You know I should be able to play the

piano like I'm going to the extremes, but it doesn't work like that. It's not to replace your hand that says to assist you cause you'll never be the IT will never be as good as what you're born with.

R: Okay, I'm just gonna ask are there any other sources of frustration or anything that you wanted to talk about or bring up?

008: I think I find that because I get the excruciating nerve and phantom limb pain. I find that there's a lot of factors there that make that worse. If I'm tired, if I'm stressed, then all adds to your frustration and I think you have to be [aware]. When I get this [future prosthesis], I have to be really aware of that, because if I've had a really crap night's sleep and I've been in a lot of pain, you know it's not gonna go as planned. I think that a holistic approach is what will be [important]. But I also find that when I am training brain training doing me the hand, I get no pain. So there's definitely a connection that using a prosthetic limb correctly can help alleviate or not make the pain [worse]. I mean, you get the odd spurt, but like you know I've been paying for. I've been paying for a couple of days. Because you tend to sort of not use the [affected] arm or whatever, I started to imagine doing stuff. It's like the brain thinks "ohh there's something there". And so it helps reduce the intensity of the pain if that makes sense.

R: Understood. Say with this pain. Did you have much pain while doing the study?

008: No, I didn't. I find any studies that I've done, any brain training that I've done that I may just get one sort of 'rogue pain' come through, but it's not like you know if the weather's crap. If I'm tired, it's like in the background of this [interview]. This this gripping burning pain, but I got none of it so. There's definitely a correlation between using prosthetics and like the phantom limb pain.

R: Really interesting.

008: I would rather feel that then be exhausted from that gripping pain all the time because it gets bad and adds to my frustration. I'm incredibly lucky I don't get depressed. I'm not anxious, just get mad. Which probably isn't a good thing, but that's why I use sport. But then by distracting myself and doing another thing that helps. So, I think all of these is not just one thing. It's not "I'll get a prosthesis and all of your all of answers [are there]", there needs to be a complete package. There's these things that go on [beyond fitting] you know, you need to cater for these things and needs to be a whole programme of understanding from professionals, researchers like yourself, and patients to say "okay, these are what you feeling. This is totally normal" and just being able to manage and know what to expect really because everybody's different. I reckon that everybody will at some point experience some of these feelings and they will be able to tick at least a handful of those boxes. There's, you know, there's a similarity with everybody.

R: It's lovely to hear to hear your thoughts. I'm going to ask you, this is touching on some of your previous experience with prosthetics. Particularly [that] you've tried a few myoelectric hands and I'm interested to hear your comparison or your thoughts on what we did versus other hands that you've used.

008: So, uh, I have done a trial of the i-Limb. I've also tried the bebionic. With both of those, they are quite – so that i-Limb is, I mean, it's lovely. It looks good, it moves quite well, but it's still very robotic. It doesn't flow, and it was quite fussy. It's nice having the app, but I felt like it was more of a gimmick. It's not something I would put on and [think] "okay, I'm going to do this task; I'm cooking a meal", it's not something I would be able to pick up. I want it to be more basic and have the important things like the infrastructure there. It's also quite heavy. The bebionic hand feels more sturdy. It's a lot more user friendly as it was a lot more simple, but still really very heavy. The hand that you had created. And I felt that was a little was a lot lighter, but it would be because it was made out of 3D plastic, but I suppose, you know? I don't know what [material] you would use if it did go into manufacture. You have to use metal, or you know that would be something that you would have to do. But that it was a little bit more… it was less jerky. I have that precision. I could pick up the blocks when I'm using the i-Limb. I was doing it, but it was it wasn't as easy as I as the one that we tried last week.

R: I'm interested – at the end there you said that you found it a bit easier to use the one last week. What in particular you might mean by that?

008: So when it came to the finer motion. The picking the pennies up. Because the action felt smoother, I felt I could control it more than if was jerky. When I had the other 2 limbs, I had them for a week or so to practise with and you would do a little bit of practise, whereas when I did your one we had what just a few hours and I was able to move that. And you know progress on to moving smaller items because I felt that the action was a lot smoother. And me thinking of that action was sort of transported or moved into the hand in a smoother movement, whereas if it was jerky, I would never have been able to do it. I think that because of 1) the weight of the bebionic and i-Limb, and 2) the jerkiness and the very stiff movements. That's the reason why it was easier to move your hand. More fluid. That's the word, 'fluid'.

R: I just want to touch on one more statement before we head towards the end of this interview. It's this statement that says "the movement of the prosthesis feels like an actual movement". For all of the tasks where you were using it, you said

agree. With the blindfold [stage 2], it was somewhat agree, but still positive. I just wanted to understand a bit more about what, what an "actual movement" feels like to you?

008: Well, it's a lot smoother. It's not a jerky movement. So when I would control it instead of it going like that [imitates a jerky movement], it was [imitates a smooth motion]. In the real world that makes sense, which is what you need, you don't wanna be able to crush something to death. And like when I was. With some of the hands that I was [trialling previously] with [the prosthetist], I don't know why I did, but I put it to my neck and the hand actually just closed on it. And then I'm thinking "****". You know, it's probably [beneficial] to idiots like me to have something a little bit smoother so that you can sort of get out of it instead of a jerky movement. So yeah, that's the only way I can explain that. I felt that had I put hands around my neck I would have been safer. Not that I would [put the hand around my neck]. And it's not a jerky movement. It felt a lot smoother. And so yeah, you feel more of an association of and that moves a little bit more like your own hand.

R: Is [there a] relationship between it being smooth and it feeling like an actual movement?

008: Yeah.

R: What exactly do you mean by smooth motion? Relating it to your own hand?

008: Well. If you had a jerky [movement]. You wouldn't be able to control it as well. So, like your own hands, sometimes you need to be gentle with things in order to pick them up, and to know whether it's a glass or know whether it's something soft or holding like human's hand. It's how you're delicate. So if the hand is smoother you have more of a connection with that hand, because it feels more human. You would be able to relate more to it. Does that make sense? If it was jerky and like sort of [C3PO – a fictional robot], you don't feel connected to it, whereas if it's a little bit smoother and mimics more like a human hand, you feel more of a connection to it.

R: Just to clarify: smoother as a word could mean just moving between jerky positions in a really smooth way.

008: It's more of 1 [continuous] movement as opposed to lots of 1234 [fixed movements]. You can go from one action to another action. When I was picking the pennies up I was able to sort of move the hand like that [smoothly] and I feel that with the other hands I've tried, it's more open, close, open, close. More fluid, maybe more, more organic, it felt more organic.

R: Okay, the so the way we describe moving between these fixed positions is called discrete control.

008: OK.

R: And the way we have this continuous motion is called continuous control. Is that what you're referencing, this sort of ability to have this continuous motion?

008: Yeah, because you're not having to switch. I'm not having to switch from right move this bit [of the hand] to move [this bit of the hand sequentially]. I have to think of 2 different actions in order to move the hand the way that I wanted it to, but where you've got that continuous [motion] it's easier to control the hand than being in jerky movements.

R: Thank you very much. My final question to you is an open question. Is there anything that we haven't talked about yet that you would like to bring up or talk about?

008: No, I don't think so. I'm trying to be part of [research] and to see what's going on in the background cause [as an amputee] you don't see what's happening. I find it's just reassuring to know that maybe not in my lifetime, but maybe in someone else's it could be possible that actually, you know you lost your limb. Yeah, it's ****. But you know, we've got one which you'll be able to be as independent as possible. I've just really enjoyed being part of it. It's nice to sort of help out and give my feedback.

S9 Participant 009

R: The first one I want to ask you about is the statement which was "the movement of the prosthesis feels like an actual movement". At the beginning, that was strongly disagree, before you'd used it. Then after you've done the position matching, moving the hands to set position shown by those green bars, that went up to somewhat agree. Then after we did the force matching, so grabbing hold of that can with a specific force, then putting a blindfold on and identifying objects that went up to strongly agree. After the final stage where you wore the hand and you did some pick and place tasks, it stayed at strongly agree. So what I wanted to ask you about is what was in your mind, what was happening during those stages to make it feel like an actual movement or not?

009: In the first part I think I was getting used to it but by the end I could actually feel the muscles on my arm moving. So every time I felt the muscles in my arms like hiding in certain muscles, I can actually feel the [prosthesis] moving. So in a way it did feel like it's part of me, like I can control it with my arm with the same movement in my arms. I could just control it. So, to me, it started getting closer to actually feeling like the movement in my arm and feeling like part of me. I can control it and it takes a lot of focus as well, it took a lot of energy mentally to move the hand so it did feel a lot closer, like I can control it so it kind of felt a lot more like a part of me.

R: You were saying there that it feels closer to you. What do you mean by that?

009: It's like… I've got a [different] prosthetic hand and can't move it, but with this one I can move it and therefore it, as I said, it takes a lot of energy. So I have to learn to control the different types of [movements], and at the same time, the muscle is just learning to find out how much pressure and how much force to put into my arm to control the fingers. It did take a lot of energy, so it did feel more like [part of me]. It's partly because I had to try and control the fingers and eventually we could feel the fingers as well. But yeah, I felt closer [to the prosthesis] because I had to control it.

R: Okay, and what about that made it feel like an actual movement?

009: To have to use my movement and then I can control this [prosthesis]. It just felt closer to that experience. It wasn't too… it's not like obviously I can do this [moves individual fingers of their right hand]. It's more [like the relationship between] more force and more tension and more focused to do it. It's different, but it's a new experience that kind of also feels a lot like [my right hand]. [It's like if] you were picking up a weight and you lifting a heavy weight. You get that force and you're lifting it but you can that feel as the muscle straining. That's what it felt like when using [the prosthetic hand]. I felt like I was building muscle. Yeah, it felt more like [an actual movement] because you can [relate it to] when you're doing weight [training] you can lift something the same way. That's what it felt like; my arms training the muscles. The more tense you get, the more it moved, so I had to try and figure out how much force to put in it. Like you would do weights, it's a new type of weight [in a] way. The more you want to close the fingers to pick up something, the more pressure, the more strength you have to use and it felt like it's gonna build the muscle like in the gym, and that's why it felt like me. That's why it's felt like closer to being an arm, because when you're picking up the weights and you lifting it and you can feel this training, that's what I found with my arm like it felt like I'm building the muscles. The more I have to focus to be able to use the arms. What because the muscles that are in my arms [were] starting to tire out, so I had to learn to control it at different times. At the beginning my arm was relaxed – it's chill – and then at the end it's like tired and exhausted and you get this lactic in the arm as well. So you it feels like the gym kind of picking up something in the gym.

R: I'm OK, the next one I'm going ask you about is that statement that says "I am in control of the prosthesis". After stage one, so after the position matching, you said somewhat agree. After stage two, so after you were grasping the can with some force and then you put a blindfold on with the object detection, you said you strongly agree. But then after stage three, where you had worn the arm and you'd done these pick and place tasks, it went down to somewhat agree again. I'm really interested in that [change]. What happened between stages 2and 3 there?

009: Stage 2 okay, I felt I enjoyed that because I can actually feel what pressure I'm placing on the certain objects and it had the feeling of when you closing it, different parts of your arms had hard pressure on it so it didn't take me long to figure out which object was [associated] with which amount of strength and which pressure. I could identify [objects] like… this object is more pressure on my left part of my arm or my right part of my arm, or it connects to all around my arm. I think it was the can or something. The screwdriver it had a sharp pressure around my arm. It wasn't too discomforting it's like just like a shock to let you know that it's picking up an object that's smaller, but if you require more strength [it applies] more pressure on like my arm. So after a while I could identify which object I'm holding because of the way it triggered in different parts of my arm. Like after that it did feel a lot like I was in control of it and it did feel part of me as well because I could identify which I'm object I'm using. What exactly was the question?

R: So you said you strongly agree to "you're in control of the prosthesis". When you then went on to the next part where you wore the prosthetic hand and then you did the picking up the small objects, stacking the checkers, doing the blocks in the

boxes, it went down to somewhat agree.

009: Yeah, that one is because even though I picked something up, I had tensed my muscles, once I tensed it I sometimes let go and I don't realise I let them go. In my arm I [paid less] attention [to the muscles] and then I might just slightly open up [the prosthesis]. And then once I did that it drops the object. So, in that way it did feel like I wasn't so much in control, but I had to learn how to maintain pressure I'm using in my arm and holding that pressure even though I was tired. I did it for a long day and so the tiredness would made it so that I couldn't control how much pressure exactly what I'm putting in and therefore I wasn't totally in control of it. I think it didn't feel as much like I had control of it or not enough experiment.

R: Understood. The next statement is "the prosthesis is in the location where you would expect your hand to be". And you said in stage 3, so when you wore it, you said you strongly agree. And yeah, I was just wondering what did you think about how the prosthesis lined up to where you would expect your hand to be?

009: I expect my other hand to be at the same place. I haven't actually put it together to find out if they actually same length. Yeah it may be slightly longer. I felt like it if I had a second hand, it would be around the same place.

R: Okay. I'm interested in this statement that says "my body feels complete". Before the study, you said strongly agree. Then, for the rest of the study, so once you started using the prosthetic hand, you said disagree.

009: Now, my body feels complete like this way. I do feel that prosthetic arm, if I had it, if it gets more advantages, it would be something that I would use for daily use. It is something that can help if I were to pick up certain objects, see if I held something small in [my right hand], and then I need to hold something else, that would be something it would be useful [for]. But yeah, I would say I feel complete now. However, with the prosthetic hand that would be something that could advance certain aspects of my life. I would say it will help build the muscle in my left arm, which would be awesome for the gym, you know.

R: So during the study you said that when you were using it, your body didn't feel complete, so you disagreed.

009: That would be because, even though I had control of it, I didn't have total control of it. It's like there were still aspects that I couldn't control, and the experimentation is good, it's great, but I just didn't have so much control of the arm as I would like it to have. Like, imagine if I can move, the [individual] fingers [that] would be cool. At the moment I can control it, but I didn't feel like I was [complete]. I don't need it [to] feel complete.

R: Just then you said you didn't have quite as much control over it as you would have liked and you would have liked to have moved the individual fingers. What about that would have made you feel complete?

009: With a left hand, I find maybe I don't need to use the fingers. We did use the opening and closing, that was good when picking up certain objects which would be good if I was to hold something and pick up something else. If I was to move the fingers and had that kind of control, that would be awesome, but I don't know how that would be possible yet. At the moment there's a stage where we were picking up objects I felt. Yeah, I could pick up some things, but I don't know how, if I was to use it, would I be able to pick up something that's heavier? Where [a prosthetic hand] would completely help me, or whether, actually, will I be doing as I am now? I am so used [life without a prosthesis]. I can do what I can do now, and I can do more stuff with my arm now [than with a prosthesis]. Like it's natural without with the other arm is not so natural in as well.

R: Okay, the next statement is "the prosthesis is my hand". In the beginning, you said strongly disagree; you hadn't used it yet. After the position matching, you said strongly disagree. After [stage 2], you said strongly disagree. Then after you wore the hand in the final stage and used it, it went up to neutral.

009: That's because, over time, I used it and it became… because I'm using the muscles again and I felt like the weights again, it did become closer to being my hand, like I had more control of it. And, to some extent, I felt like it was part of my arm because the amount of exercise in my arm. So therefore it felt like part of my arm, I have control of it. So yeah, it kind of felt like in the middle of [the study] yes, I did. I still didn't feel like sensation of the arm where I can touch it or and I feel the movement exactly but I can feel the movement in my arm when I tense in my arm itself. It did feel like my arm and I can feel the [prosthesis] on my arm, so it did feel it might not be exactly my arm [that can be] touched and I can feel the sensation but I did feel the [prosthesis] via the muscle movement in my arm if that makes sense.

R: Okay, so you when you were wearing it, you could feel it on your arm?

009: I could feel it on my arm and I can feel the muscles moving, so therefore I had control of it but as I think of [my right arm] now you can touch it and sense that and I can move it I can feel connected to it. With the prosthesis I can only feel what's on my arm at that moment, other than that I can't do anything about it. I have no sensation on the [prosthesis] so I can't feel the arm itself. I can feel the [prosthesis] being on my arm so I can feel part of it. The prosthetic hand being

on my arm and I can feel the muscles being moved and controlling the hand, but I can't feel the actual hand itself as in touching it.

R: Ah okay, and a touch sensation would be quite important?

009: If you wanted to make it more like a hand, you would feel it. So say if you can touch your hand now, you can feel the sensation, then you know it's your hand. At this stage it's like I can feel that the prosthesis is on my arm, but other than that you can't feel the external sensation of the hand. You only know your it's connected to you because you're controlling the fingers by the muscles [in my arm]. If you can have the touch like, say if you can touch it, I would say that will grow to become more like you, become closer to your hand.

R: Okay, and what about the haptic armband, what did you think about that?

009: It helped. The speed and the amount of pressure. You can control the muscles in the arm, but once got that around you and you can feel like I was saying about the pick up the cans and certain objects. You can sense what objects you're picking up even when you have the blindfold on. I could feel which object I'm picking because it is triggering different parts of the armband, so different parts of the band had some sensation where there's a poking here on the right or there's a poking on the left, or maybe it's all of the arm, or maybe it's like a clenching kind of feeling where you can something's holding on to your arm and then a poking. At some points it could be the poking on the left or the front so you get different associations, so you kind of know which objects you're picking up but. Yeah, I like that experiment. It's only when you tricked me when you had the same object three times in a row.

R: It's a randomly generated order. I'm gonna ask you about how mentally demanding? So the first stage, the position matching, you said it was 5% mentally demanding. The second stage you said 0%; not mentally demanding at all, and then the third stage where you were picking up the small objects, you said 0% again. I was wondering specifically about that middle stage with the blindfold. What was it about that that made it less mentally demanding?

009: First one, the positioning, didn't feel mentally demanding. Second parts of that when you talk about now, that is because it I could sense the object. And the hand was clenched on the object. I wasn't turning the hand, it was just clenching down. I use that muscle all the time, that clenching muscle, I use that myself quite often. If I was to pick up a bag of shopping, I use it to pick that up so that muscle didn't really take much energy or demand for me. Because I'm just picking up the object and just clenching down until I can feel the object and then I can feel the sensation on my arm with that band. So it didn't take much; it wasn't mentally demanding at that stage. The last stage, that's when I got tired, which I think the certain objects [that I picked up] were smaller and that is a lot of… it was hard. Picking [objects up off] the table is really difficult, you have to be so focused to pick up each object, that takes so much energy.

R: When you were talking just then, you said that the first stage wasn't that mentally demanding?

009: It wasn't when we had to learn to control to close and then turn it. Yeah, that that that stage was just practising the different levels of pressure I had to use. It didn't feel so demanding. It's more exciting.

R: You actually rated that one as the most mentally demanding.

009: I'm sure it wasn't that demanding. I would have thought [in hindsight] that the last one would be more demanding. At the end, yeah, picking up tiny things.

R: So mental demand, you said that you don't remember the first stage as being very mentally demanding, but at the time you said it was quite mentally demanding.

009: Maybe because I was not used to it. It might have been taking a lot more out to me from figuring out how much pressure, how much of that muscle I need to use. Maybe that's why I said it.

R: OK, we're going to look at physical demand now. You rated the first stage as pretty high, so that one you rated as 90 out of 100. The second stage was a lot lower at 20, and then the third stage you also rated low as well at 20.

009: The third stage, that was the most [physically] demanding. I think it would have been but I said no… why would I say no? Maybe I got used to the arm and that I can control it. I knew how much pressure [to apply with my muscles], that would make sense as to how it wasn't so demanding. The only thing is I used it for a couple of hours, and picking up objects [was difficult] too. I think we picked up the coins, and the cans – the cans were easier. I found that easier but the coins and the bottle caps I love. The bottle caps were easy to pick, that was awesome. I enjoyed that one.

R: Why was that awesome?

009: I don't know, it's like the hand just felt like… it was effortless. I could just pick [them] up. It felt like my arm is actually my arm and at that point it just felt like I knew what to do. It was like a sense of "I'll pick [the object] up and put it [in the can]". It was easy, but then the other thing was that the coins were more difficult. I had to change the hand angle then

learn [that technique] too. If you had to pick off the table to move it, and then how much pressure you use, and then to not lose the pressure you put on to it, because if you do that then you drop the coin and then you have to go back and do it all again. I think some parts wouldn't be demanding, but that coin was a little bit demanding, would say then overall it's not demanding. I felt like a lot like the arm was part of me. It was effortless. The coins... I enjoyed it because I couldn't do it. I wanted to do it again until I found out how to do it. It was fun. It was definitely fun, because it was the only experiment that day, I think, that challenged me more. And because it challenged me, I thought "okay, I want to do this task", just to make sure I do it better than I did.

R: The ohh I've got two more that I want to ask you about. The first one is about performance. So this is what you thought about your performance, not the actual numbers themselves. So in the first stage you said 80 out of 100 – that's quite high. The second stage, after the blindfold, you rated as 45 out of 100. Then the third stage you rated as 75 out of 100n.

009: The second stage, that is because I wasn't sure if I had it correct. I didn't know until afterwards. [Afterwards] I thought "ohh, that makes sense" because I can feel the motion and the pressure on my arm so I know which object I'm picking up, but at the time I was kind of confused. Especially when there were three [of the same] object in a row, I couldn't believe it. My expectation was that I didn't do as well for stage two. For [stage] one, I felt like I did well. I [could] checking easily, see what I'm doing and I was aware [that] the pressure [in my muscles] this rotated to this side or this much pressure to clench this side. That's why I felt I had a higher performance there and the third [stage], which was picking up some things, that was high. I was able to learn to figure how precisely I wanted to move the arm and when to grasp and learn to actually control it. It took a lot of attention to do it; a lot of effort to pick up something. I enjoyed it because it's like training; it's making my arms "jacked" and you get my arms tired. I felt better on that one because I [like] more of a challenge and I just kept wanting to do more to see how fast I can get it.

R: Okay, you could see yourself learning how to do these?

009: Think I could do it all effortlessly in the future. Especially if I could get a [prosthetic] arm, I would think everything would be easier, it would be automatic eventually knowing how much pressure I need to put in my arm and how much to let go at some points when I'm picking to unlock and drop something.

R: The last question I'm going to ask you about is frustration. You rated it as low as possible for the entire study.

009: Yeah, it wasn't frustrating. It's mentally demanding sometimes, but it wasn't frustrating. I enjoyed it – this is fun for me. It was never frustrating at all.

S10 Participant 010

R: As a quick refresher of what we did, there were 3 stages to the study. The first one, stage 1 was matching positions with the prosthetic hand that was on the screen. We had the green bars and then we had some red bars that we were controlling them to get up to that level. Stage 2 was doing the same thing, but for a target force, so it was squeezing hold of a can and trying to match the force. Then putting a blindfold on and identifying objects in the hand. Stage 3 was wearing the hand and doing some tasks like moving the cubes from one box to another and picking and placing objects. Select picking up paper clips, the cans and the full cans, the heavy ones. We didn't do the paper clips because of the elbow of the socket not being present, so wouldn't have been able to get a suitable angle to make that task possible. But we did do stacking checkers. We did do flipping over pieces of paper and stuff like that. I'm going to ask you about a few of the things we talked about in the questionnaire that we filled in after each stage. We're going to start off with mental demand. For stage 1, with the position matching experiment you had that down as 15 out of 100. After stage 2, with the force matching in the blindfold that went up to 40 out of 100. Then for stage 3, that went up to 70 out of 100. I'm interested to know what you can remember about the mental demand side of using the prosthetic hand for each of these stages.

010: I think for the first one, I think it's because I haven't used any of the muscles or any of the impulses in that arm at all. Now I've got this [static prosthesis] on, I've been doing something outside with this [prosthesis] on and it is quite useful to me. But, I have to do everything [manually] with it. With [the myoelectric hand], I moved my arm and it moves. But when I'm asked to like grip something, I've got to think about it and that's where the mental demand comes in. To match the red bars to the green bars - I've actually got to think hard about it. For a few days afterwards [I felt] quite stiff because I've never like done anything like that for 3 1/2 years, and to do all that in one day was quite mentally taxing, physically taxing.

R: So having to think about moving the muscles in your arm to make the hand move was quite mentally demanding? On stage 3 where you were doing it with task when you were trying to complete tasks, did that make it more mentally demanding?

010: If we separated it into three separate days, with a bit of a break in between. Maybe it wouldn't have been so bad, but because we aimed to get it all done in one day, it was quite taxing. I mean, just not just physically, but just the thought of it just thinking about what I've done. Technically speaking, all I'd done is sit down all day and thought about what my arm's doing and making me hand do what I wanted it to do. I was tired. If I was using muscles on a daily basis they'd probably get quicker or looser. I can still [move my muscles] now; I can still think about it and do it now, but it feels sluggish because [I don't use them]. I don't know whether they'd loosen up overtime or don't know. At the moment I can still move my thumb and my two index fingers… I can still move them quite freely but my ring finger and little finger are slower. Whether a more sensitive [electrode] could pick that up and activate a hand with all the nerves and the muscles.

R: Okay the next one I wanted to talk about is performance; how well you think you did at the task. For the position matching stage, you said 70 out of 100.

010: Is that is that the green bars and the red bars?

R: Yeah, that was the green bars and the red bars.

010: I think that doing it on that day cold. I don't think that I, personally, did too badly. I was getting the hang of it. With more use, I'd probably get better.

R: Then when we did the blindfold task with the objects, and performance was down at 35 out of 100.

010: I don't think it did that well on that one because everything happened on the one day. If I got the hand and took it home and got [used to] the feedback. But trying to it [with less practice] was a bit confusing for me. It was hard enough to get the that the hand to work and do what you wanted. Recognising like a can and getting the feedback and recognising how much feedback you get off that as opposed to the smaller objects like the squishy brick, that was tough for me.

R: Okay so it was made harder because you had to concentrate on how to move the hand and then it got more complicated because you were trying to figure out the feedback at the same time?

010: Yeah, it was hard enough. I mean, I was getting used to it. The more we did it, the more I got used to actually operating the arm. When we took a reference of the two fingers and the thumb, and then [another two references] moving the wrist up and down. Getting that to work with the hand… I was concentrating on doing that and then trying to recognise what feedback I get from individual items. That was the hard thing for me. If I could take the hand home use it a lot, I'd get used to what feedback there was. But on that particular day because we did it all in one go, it was a bit confusing. I think I didn't fail, but I just I didn't do as well as I probably could have. In time, I probably would get better.

R: That makes sense. Okay and then for the third stage, when you were wearing the hand and doing tasks with it, you had it down at 90 out of 100. So very high performance.

010: I think that was because we eased into it. When I was picking the paper up you coached me a little bit.

R: Yeah, we had practise beforehand.

010: I mean a lot after that is because of the orientation of the hand. It's not an ideal hand, you've got to manoeuvre it. But the more practise you get with it, the better you get at it.

R: The next one I'd like to talk about is frustration. On stage 1, you said it was very low, it was 5 out of 100. In stage 2 that went up to 30 out of 100. Then stage 3 was 30 out of 100 again. Do you remember what it was that contributed to feeling frustrated or not frustrated?

010: Frustration… I do still get frustrated now trying to do things, when I know I've got to do things or I'm trying to do things. With a lot of communicating with the hand and making the hand do things that I want it to do. It's all foreign equipment to me. I wanted to do better – when I'm trying to do something and it doesn't work, that's when I get frustrated. I get frustrated most days, but it's just because I'm expecting to do things, and it working. When it doesn't work that's where the frustration comes in. It's the same with the hand. Is that the about the communication between like my brain and the [electrodes]? It's the time thing: I'm trying to make it do something that I want you to do, but I'm waiting for the system catch up with what my brain's trying to do, and that's where the frustration comes in.

R: Okay. And throughout the study, though, your frustration was you when we asked you about it, you always said it was quite low. Do you have any idea why it was so low?

010: I think part of it is because I think part of me part of me knows that it still works and given the right sort of equipment, it will probably work better. So I'm not as frustrated as I could have been. Knowing that the stuff that you've made does actually work and then me getting it to work as it should do. I could have got more frustrated but I I'm not a frustration kind of person. I've got more frustrated that have lost my arm but I'm pragmatic. If there's something to be frustrated about, I will. But if there isn't, I don't. My wife will disagree – she said I've got more frustrated and more angry since I've lost my arm. I don't think I am. We'll agree to disagree.

R: Okay, out of interest – why would your wife say that you've got more frustrated since losing your arm?

010: [She'd say that] my frustration levels of gone up since I've lost my arm. I don't think they have. I get frustrated. I don't get frustrated with her. I get frustrated with myself when I'm on my own and I'm trying to do something. For instance, if I can't open a jar and I'll use one of my tools to try and open it and still can't open it. I think that's natural because but I've never [struggled to do] that before. I didn't [struggle] to do it before. I do get frustrated a lot more. I don't get angry, just frustration.

R: Okay, so when you can't do something that you could have done before – is that a frustrating feeling?

010: I could get overly frustrated about, like, putting on shoes. Nice shoes have laces, and I can't do the laces, but I bought elasticated laces or slip on shoes now so that that's a frustration that I haven't got anymore. I could get frustrated, but I don't.

R: This independence?

010: I don't like to ask anybody for anything. I don't ask anybody for help. I just want to do everything myself. Knowing full well that it takes that much longer to do it. If I ask for help, obviously the help is there. But like, for instance, if I needed to go out before I got my car back and everything, and if I wanted to go into town, I'd rely on my wife to take in town, knowing that I could have asked other family to help. Or I could have asked my mate or anybody to help. I just wouldn't. I'd rather either walk, get the bus or rely on my wife. It's an independence thing. When you've always had your independence and it's taken away from you. I draw a line if there's something I cannot physically do, then I will ask. I'm a proud man. But if there's something I cannot do physically, then I'll ask. I'm not stupid, but I prefer to be able to do things myself. Like right now I'm retired, so if there's something I need to do, I'll take that much more time. I need [more time], but I'll get it done. It's just part and parcel of an amputation. I will get things done, because I'm determined to get them done. Like today, I've been chopping logs and I've used this [static prosthesis] for the first time because it's easier to sweep up. I [usually] do it one handed, and it tends to like wear this arm out at my shoulder. Doing it today with [the static prosthesis] – so much easier. I've got something to actually brace the brush against. I shall use it more, for cutting the hedges and stuff like that. I'll use it more. I do get frustrated but if I can find a way around something, I'll find a way around it. On the day that you came because all the equipment was new to me and stuff like that, I was a little frustrated. I wanted it to be better, but because the equipment was new to me and everything, it was mildly frustrating, but not overly so. The equipment's new to me. You're out in the field doing research, so things don't necessarily work. So this first the levels of frustration, depending on what happens. It would have been lovely for you to come and everything worked perfectly, but it didn't. It would have been lovely for me to put the arm on, put the

[electrodes] on and get the feedback and everything to have worked perfectly but doesn't work that way. That's that's life.

R: Yeah. I'm going to move on to the [embodiment statements]. The first one I would like to ask you about is the statement that said "the prosthesis is my hand". Before we started, before you'd used it at all, you already said you somewhat agree. Then after you'd used it, and it stayed this way for the rest of the study, you said agree. I was wondering what it was that made it feel like it was your hand.

010: I think initially it's somewhat agree in that it felt part of my arm, but only to a small degree because I've never used it before. Having something there that I can actually manipulate, move. The more that I operated it the more it felt like it actually it was or it could be my hand in the fullness of time, when technology gets better and money's more available. I can go to America now if I've got like 100 grand or 250 grand and I could get one fitted perfectly and it will work perfectly. But until then, it's just luck. That would feel like a proper arm. I mean I look at this [static prosthesis] now, it's practical – it doesn't look like me all. The more you get to used something, though. I'd like a hand and arm, just so that I can use like a pair of hands again like instead of one. I've noticed different things when we've been away on holiday and things like that. [Myself and my wife] think that people don't come up to us now because I'm obviously not wearing an arm. They don't come talking to us now or interact with us because I'm disabled, we get that feeling as if it's because I've got no arm. Whereas we went with another couple and people were talking to them a lot, weren't they? But they wouldn't interact with us. It's like because you've got no arm. People tend to like, stay away from me. As if I've got the plague or something. I [know a person], if he's wearing like a suit, something like that, he's got this [prosthetic] hand there that's just static. That's just sitting there. I'd rather not do that.

R: You mentioned when you were speaking a minute ago. Were comparing the one you're wearing at the moment, which is a static prosthesis with a… it's like a claw, but it's more like a clamp, right?

010: Yeah.

R: And you were saying that you've got this one, but it doesn't like look like a hand, and the one we used in the study looked more like a hand that you were you moving around? How does the look of it – it having four fingers and a thumb, how does that come into play with it feeling more or less like your hand?

010: Well, because it looked like a hand. The one that you brought down, that is like an active one: it will actually move. This [static prosthesis] doesn't move and it won't move, it just basically [clamps]. Whereas the one that you brought down actually [produced], you know, movement in each [finger].

R: Okay to follow up on that slightly. Let's say that I brought [the prosthesis] and it looked the same as that [static prosthesis] there, but you could move it. I'm trying to understand, I guess, how important is it to you to have, say, fingers and a thumb that move?

010: If I'm going to have a prosthetic hand, I'd like a proper prosthetic arm that actually does work. Whether they will work with my little finger and ring finger [I'm not sure]. [In those fingers] I feel a little movement, but it's very slow, but if I had [a prosthetic hand] with just the three fingers on but I could move them quite well, that'll be that'll be enough. I'll be happy with just three, but I think if there's the possibility of getting all four fingers and a thumb working. If I'm going to have a hand, I'd want all of them to work. But whether they all would or not, I don't know.

R: What I'm trying to understand is, in your mind, the different factors that make up something being your hand. You've mentioned how important it is for it to be able to move. Is that to do with you being in control of it?

010: Well, I suppose so, yeah. Suppose it would feel a bit like normality, wouldn't it?

R: Or is it being able to see it move or is it being able to feel it move or something else?

010: I think, in part, it's all of them. My brain knows it can actually [control a prosthesis] now. I've always known that I could do it. I can physically [move the muscles]… It's a bit of everything really. If I'm going to have [a prosthesis] I want it all to work. If I can get it all to work then it feel more like my hand. This [static prosthesis] just feels like a tool. With the with the [tasks] that I want to do, for 3 1/2 years now, I've done without [a prosthesis] and I can do 85% to 90% of what I could do before. It takes a little bit more time. With this [static prosthesis], or the other [static prosthesis] for the push bike and [playing] pool, I know exactly what they're for. I don't tend to use them for anything else apart from the push bike and pool. I know this has only got a certain amount of light usability if you like. I'm quite happy with what it can do. But having a hand to be able to do 95% to 100% of what I could do before… In time it will come, but at the moment it's a long way off. Knowing that all the impulses are there to do movements like a normal hand. That's given me hope.

R: Okay, I'm going to ask you about the next statement. The next statement is "the prosthesis belongs to me". Before we started, you said strongly disagree. After the green bars, matching the position it went up slightly to disagree instead of strongly disagreed. Then, after the blindfold task that went up again slightly to somewhat disagree. After you'd worn it,

it went up to agree. What made it feel more or less like it belonged to you.

010: I don't know really. With more use it could. In terms of belonging to me, I know it's only a research and development 'tool' if you like. It's never going to belong to me. But if it was developed more? It should [feel like it belongs to me]. I mean, part of it is just wanting a hand again. Wanting it to belong to me. When it's fully working and I can actually use it on a daily basis, then it will feel more like it belongs to me. I know that one's not going to belong to me because, it's not quite finished. You're developing it now, but in five or ten years' time, there are going to be leaps and bounds [of development]. Whether I'm going to be young enough to benefit from that, I don't know. It will come in time; you'll go to the shop and say "ohh I've I've lost my arm". Then you'll just put [a prosthesis] on and it will work. It's like anything in medicine goes in leaps and bounds, so in time I will get one, but whether I'll be too old for it, I don't know. Then it will belong to me.

R: That makes sense. So in stage 3, the other part that I'd like to ask about is: when you wore it, was there anything about putting this prosthetic hand onto your arm that changed the way you felt about it.?

010: It's still early days. The equipment, the feedback actuators. They need more work because they are quite basic and quite bulky. But in years to come, you'll just slip it on and that'll be it or it'll be engineered into your arm.

R: Okay. The next one is "I feel as if I'm looking directly at my own hand rather than that of prosthesis". Now for this one, after stage 1 that went up very slightly to disagree. After stage 2, after the blindfold task, that went slightly up again to the somewhat disagree, then after you've worn it, it went up slightly to neutral. What would it be about a prosthetic hand that would make it feel more or less like it looked like you're looking directly at your own hand?

010: Familiarity with it, to be honest. Everything on that day was all new to me. The more you wear it, and the more you get used to it, and the more you can operate it the way you want to do. By the end of the day, it was good to know that I can actually use it and it worked.

R: I remember you saying at the time while you were filling out [the questionnaire], you said, "well, it's red, this isn't going to look like my own hand".

010: It's just appearance that is, that's just appearance. I mean, I'm never going to have one that's going to look like my own hand again. Without an arm, people are going to stare. With a prosthetic arm that's flesh coloured on people are going to stare. I would rather have something to stare at, that's why I've gone for the carbon fibre and aluminium [static prosthesis]. If I have the option, I'll have a Terminator one. I'm at the point of life now, where I just don't care. People can point at me, stare at me, laugh at me. I really don't care. Doesn't bother me one little bit. I've been out on my bike and the kids are coming out of school. On a sunny day you can see I've only got one arm, but it's like a different arm. I've never had any sort of like bad things happen and never had any sort of bad things said to me. Some people with a disability, like an amputated leg or amputated arm, they wouldn't go out because they feel so self-conscious about it. But I'm not going to live my life [like that] just because I've lost something.

R: Okay, I'm going to ask you about the next statement. This next one is "my body feels complete". At the beginning, when we asked, you said you strongly disagree. Then when we started using the prosthetic hand and for the rest of the study, you said you somewhat agree. I was wondering if you could shed some light on that a little bit about what makes your body feel complete.

010: At this particular moment in time, I know my body is not complete because I can look at [my arm], it's gone. I know it's never going to be complete again. Even with the best prosthetics going, it's never going to be complete because I know that bit is not quite real. As long as it will do what I wanted to do, I'll be happy. I know it's never going to be complete, but I can't do anything about that. I'll get something like a prosthetic arm that actually does what I want it to do when I want it to do it. It's like this [static prosthesis], I've actually got a physically think about [donning] this one to do the job that I want to do. The rest of the time I don't bother wearing anything. I've actually got to physically think about the tasks I'm going to do. When it comes to the time when I don't have to think about that, I'll just get up in the morning, put it on and it work exactly like my arm. Then it'll be more a part of me.

R: This idea that it could do many tasks instead of just one task?

010: Ideally, in time, I will get one that will do everything I wanted to do. Whereas at the moment the ones I've got they do specific tasks. I've got to physically put it in a bag, take it with me, put it on when I get there. When I go out on my bike, I've got to physically put it on. When the day comes when I don't have to think about that. That'll be completely different altogether. They can keep supplying me with all the tools and terminations that will do the things that I wanted to do, that's all well and good. Until the time that I can actually get an arm that I don't have to think about. To be complete with a normal arm that will do everything, that's the goal. At the moment I'm just a man with one arm and some tools.

R: Interesting.

010: I'm used to the versatility that I had before because I was an engineer. I was using both hands everyday. When you lose one of them, it takes a bit of getting used to. Until I get that versatility back, which I don't think I ever will, as long as I can get to 80% of what I could do before. And if that 80% comes with a range of tools, that's fine. I'm never going to be like the man that I was. I'm not going to be complete again, but I accept it.

R: Thank you for that, it was very in depth. The next one I would like to ask about is "the prosthesis moves the way you want it to move". After the position matching task, you said neutral, then after the blindfold task it went up to somewhat agree. Then after you wore it and did tasks with it, it went up to agree. Do you remember how the prosthesis was moving and how it related to how you wanted it to move versus how it was actually moving?

010: I had to move my [phantom] hand in a certain way. Like if I moved my hand downwards, that rotates the [prosthesis]. It's a bit like I'm trying to make [the prosthesis] do one thing, but you've got to make your [phantom] do something different. That's a bit confusing for me, I was expecting to rotate my [phantom] hand. But like you said, because of all the nerves are close together, picking individual [actions] up [is challenging]. It takes a bit of getting used to.

R: Okay and the next one is "I am in control of the prosthesis". For every stage here you said you agree. Do you remember much about feeling the sense of control over the prosthesis?

010: Initially, I didn't think it was going to work at all. But seeing it actually work gave me [a feeling] like watching [something that I can actually control. I felt happy that I could control it from the word go. I didn't feel it was there was a time when I wouldn't, when I couldn't control it. The longer it went on, the less control I'd got because I was physically getting tired. I hadn't used those muscles or anything like that for 3 1/2 years. And doing all that in one go [was tiring], but it was good. It was good to see at the end of the day, that I could control it to a degree.

R: I'm going to ask about the last statement now, which is "the movement of the prosthesis feels like an actual movement". So after the position matching task you said you agree. Then after the blindfold task, it went down slightly to somewhat agree, and it stayed there for the rest of the study. What about it made it feel more or less like an actual movement that you were making?

010: That's because I can actually physically see something moving. Right now I can do all the controls just by thinking about it. But actually being able to physically see with my eyes, that's what made it more believable, more real. I can still do it now, but that's just me thinking about it and imagining my arm there doing it. Being able to actually see [the prosthesis] do it, that's the bit that made it more real.

R: Did being able to see [the prosthesis] do that change how much, say, if felt like part of your body or part belonging to you?

010: Because I could think it and see [the prosthesis] do it. That made it feel more part of my body and more real. Instead of just thinking about it and thinking it's doing it, but seeing [the prosthesis] doing it, that made it all real.

R: Okay. We've gone through all of the statements I wanted to talk about specifically. Before we finish I'd like to ask you if there's anything you would like to talk about that we haven't covered already?

010: No, I don't think so. I enjoyed the day it was informative, and it does give me hope for the future. Knowing that the impulses are still there and I can still do most of what my hand could do. I enjoyed it. It was good.

Supplementary References

72. A. van Boxtel, A.J.W. Boelhouwer, and A.R. Bos. Optimal EMG signal bandwidth and interelectrode distance for the recording of acoustic, electrocutaneous, and photic blink reflexes. *Psychophysiology*, 35(6):690–697, 11 1998.

73. Yossi Rubner, Carlo Tomasi, and Leonidas J Guibas. The earth mover's distance as a metric for image retrieval. *International journal of computer vision*, 40(2):99–121, 2000.

74. D Farina, N Jiang, H Rehbaum, A Holobar, B Graimann, H Dietl, and O C Aszmann. The Extraction of Neural Information from the Surface EMG for the Control of Upper-Limb Prostheses: Emerging Avenues and Challenges. *IEEE Transactions on Neural Systems and Rehabilitation Engineering*, 22(4):797–809, 2014.

75. Jean Feydy, Thibault Séjourné, François-Xavier Vialard, Shun-Ichi Amari, Alain Trouvé, and Gabriel Peyré. Interpolating between Optimal Transport and MMD using Sinkhorn Divergences. In *AISTATS*, pages 2681–2690, 2019.
